# aPKC Inhibition by Par3 CR3 Flanking Regions Controls Substrate Access and Underpins Apical-Junctional Polarization

**DOI:** 10.1016/j.devcel.2016.07.018

**Published:** 2016-08-22

**Authors:** Erika V. Soriano, Marina E. Ivanova, Georgina Fletcher, Philippe Riou, Philip P. Knowles, Karin Barnouin, Andrew Purkiss, Brenda Kostelecky, Peter Saiu, Mark Linch, Ahmed Elbediwy, Svend Kjær, Nicola O’Reilly, Ambrosius P. Snijders, Peter J. Parker, Barry J. Thompson, Neil Q. McDonald

**Affiliations:** 1 Structural Biology, The Francis Crick Institute, 44 Lincoln's Inn Fields, London WC2A 3LY, UK; 2 Epithelial Biology, The Francis Crick Institute, 44 Lincoln's Inn Fields, London WC2A 3LY, UK; 3 Protein Phosphorylation Laboratories, The Francis Crick Institute, 44 Lincoln's Inn Fields, London WC2A 3LY, UK; 4 Protein Analysis, The Francis Crick Institute, 44 Lincoln's Inn Fields, London WC2A 3LY, UK; 5 Peptide Chemistry, The Francis Crick Institute, 44 Lincoln's Inn Fields, London WC2A 3LY, UK; 6 Protein Purification Facilities, The Francis Crick Institute, 44 Lincoln's Inn Fields, London WC2A 3LY, UK; 7 Division of Cancer Studies, King's College London, London SE1 1UL, UK; 8 Institute of Structural and Molecular Biology, School of Biological Science, Birkbeck College, Malet Street, London WC1E 7HX, UK

## Abstract

Atypical protein kinase C (aPKC) is a key apical-basal polarity determinant and Par complex component. It is recruited by Par3/Baz (Bazooka in *Drosophila*) into epithelial apical domains through high-affinity interaction. Paradoxically, aPKC also phosphorylates Par3/Baz, provoking its relocalization to adherens junctions (AJs). We show that Par3 conserved region 3 (CR3) forms a tight inhibitory complex with a primed aPKC kinase domain, blocking substrate access. A CR3 motif flanking its PKC consensus site disrupts the aPKC kinase N lobe, separating P-loop/αB/αC contacts. A second CR3 motif provides a high-affinity anchor. Mutation of either motif switches CR3 to an efficient in vitro substrate by exposing its phospho-acceptor site. In vivo, mutation of either CR3 motif alters Par3/Baz localization from apical to AJs. Our results reveal how Par3/Baz CR3 can antagonize aPKC in stable apical Par complexes and suggests that modulation of CR3 inhibitory arms or opposing aPKC pockets would perturb the interaction, promoting Par3/Baz phosphorylation.

## Highlights

• Sequences flanking the Par3 CR3 consensus PKC site cooperate to inhibit aPKC
• A Par3 CR3 inhibitory arm disrupts aPKC P-loop/αB/αC contacts and αC-helix position
• Mutating either CR3 arm switches Par3 into an efficient aPKC substrate in vitro
• Equivalent Bazooka substitutions alter its apical localization to AJs in vivo

## Graphical Abstract

**Figure:**
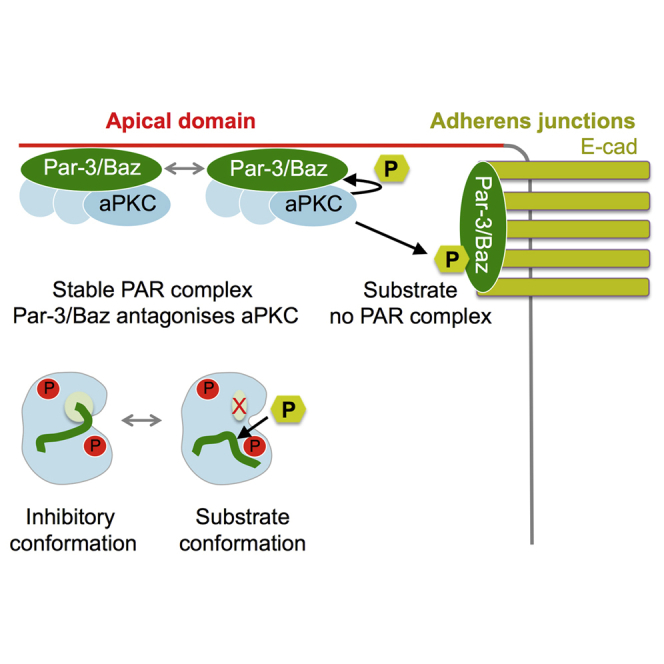

## Teaser

Par3 is required for aPKC membrane recruitment, yet it polarizes to adherens junctions upon phosphorylation. Soriano et al. show that Par3 antagonizes active aPKC kinase by separating crucial N-lobe contacts. Disrupting high-affinity Par3 contacts switches it to an efficient aPKC substrate and polarizes Par3/Bazooka from apical domains to adherens junctions.

## Introduction

Epithelial tissues are composed of sheets of polarized cells that are connected by adherens junctions (AJs) (Laprise and Tepass, 2011, St Johnston and Ahringer, 2010, Suzuki and Ohno, 2006). The plasma membrane of epithelial cells is segregated into apical and basolateral domains, with a prominent belt of AJs located at the interface of these two domains (Figures S1A and S1B). The atypical protein kinase C (aPKC in *Drosophila* or PKCι/PKCζ isozymes in mammals), its binding partner Par6, and the small guanosine triphosphatase Cdc42 are three essential determinants of apical membrane identity in both *Drosophila* and mammals (Fletcher et al., 2012, Harris and Tepass, 2008, Hutterer et al., 2004, Izumi et al., 1998, Joberty et al., 2000, Lin et al., 2000, Wodarz et al., 2000). The aPKC-Par6-Cdc42 assembly can form a larger stable complex with Par3/Baz (Bazooka [Baz] in *Drosophila*) (known as the Par complex) at the apical membrane (Izumi et al., 1998, Joberty et al., 2000, Lin et al., 2000, Wodarz et al., 2000). Association of Par3 with the basolateral membrane is prevented by phosphorylation of its lipid-binding domain by the basolateral kinase Par1 (Benton and St Johnston, 2003b).

Importantly, a distinct pool of Par3/Baz can also segregate away from apical aPKC-Par6-Cdc42 and localize to AJs (Morais-de-Sa et al., 2010, Walther and Pichaud, 2010). The role of Par3/Baz at AJs is thought to be essential as it involves defining the position of AJs during the establishment of epithelial polarity (Wang et al., 2012b) and possibly also remodeling of AJs as tissues undergo morphogenetic change (Walther and Pichaud, 2010). The regulation of this switch of Par3/Baz subcellular localization from the apical membrane to AJs has been shown in *Drosophila* to be dependent on aPKC phosphorylating Par3/Baz on serine 980 in vivo (Morais-de-Sa et al., 2010, Walther and Pichaud, 2010). This site is within a consensus PKC phosphorylation R-X-S-Ψ motif and is equivalent to serine 827 of human Par3 (Figure S1D), both sites map to Par3/Baz conserved region 3 (CR3), a site of regulated protein interaction (Nagai-Tamai et al., 2002). However, how Par3/Baz switches from being a stable binding partner of aPKC in the Par complex to being a substrate of aPKC that segregates away from the Par complex remains unclear. In mammalian cells, a similar conundrum exists whereby Par3 is critical for the recruitment of PKCι to the apical membrane and is known to be an in vivo substrate of PKCι, but loss of Par3 in transformed epithelial cells can lead to PKCι activation and can result in breast tumorigenesis and metastasis (McCaffrey and Macara, 2009, McCaffrey et al., 2012).

One complication in understanding the role of Par3/Baz in *Drosophila* epithelia is the presence of another key apical determinant, Crumbs (Crb) (Tepass, 1996). Like Par3/Baz, Crb can localize apically in a complex with Stardust (Sdt) (Bilder et al., 2003, Roh et al., 2003, Tanentzapf and Tepass, 2003, Tepass, 1996) and aPKC-Par6-Cdc42 (called the Crb complex) (Fletcher et al., 2012, Harris and Tepass, 2008, Morais-de-Sa et al., 2010). Par3/Baz and Crb-Sdt can therefore act in a semi-redundant fashion to specify the apical domain in *Drosophila*, such that either Par3/Baz or Crb-Sdt is usually sufficient to maintain polarity in *Drosophila* (Fletcher et al., 2012, Tanentzapf and Tepass, 2003). Similarly, Willin, a FERM-domain protein, has been implicated in another Par3-independent apical domain recruitment mechanism for Par6-aPKC (Ishiuchi and Takeichi, 2011). The presence of Crb has been shown to promote Par3/Baz localization to AJs (Morais-de-Sa et al., 2010, Walther and Pichaud, 2010). However, in the absence of Crb, some Par3/Baz can still be phosphorylated by aPKC on S980 so that it localizes to AJs (Morais-de-Sa et al., 2010). These findings indicate that individual Par3/Baz molecules can localize either apically or junctionally without requiring any input from Crb. Thus, the paradoxical dual role of Par3/Baz as either a Par complex component or an aPKC substrate appears to be an emergent property of these molecules themselves, although it is still uncertain how this property arises.

aPKC isoforms PKCι and PKCζ have regulatory regions distinct from those of other PKC isozymes, but share a conserved catalytic protein kinase domain (Parker and Murray-Rust, 2004). They are not responsive to diacylglycerol and have less well-defined activators (Limatola et al., 1994). Like many protein kinases, activation of aPKC requires activation-loop phosphorylation and an αC-helix conformation compatible with Lys-Glu salt-bridge formation to bind ATP and serve to align residues within the R spine (Kornev et al., 2008). Functionally validated aPKC substrates include Par3, LLGL2, ROCK1, and MARK2, and the Hippo pathway component Kibra (Betschinger et al., 2005, Buther et al., 2004, Hurov et al., 2004, Ishiuchi and Takeichi, 2011). Sequences flanking the phospho-acceptor site in each aPKC substrate are rich in basic residues consistent with basophilic AGC kinase consensus sites derived from short peptide substrates (4–14 residues) (https://www.kinexus.ca). In these contexts aPKC phosphorylation inactivates substrates with basophilic membrane-binding motifs with embedded phosphorylation sites such that they are displaced from membranes (Bailey and Prehoda, 2015).

Here, we describe how Par3 CR3 recognizes and inhibits a nucleotide-occupied primed PKCι. Two Par3 CR3 motifs flanking its PKC consensus site engage pockets within the PKCι kinase domain, one of which disrupts crucial N-lobe contacts required for catalytic activity. A second contact used by both aPKC inhibitors and substrates provides a high-affinity anchor point through a Phe-X-Arg motif. Together, both motifs cooperate to block aPKC substrate access and prevent phospho-transfer to Par3 CR3. Mutation of either motif switches Par3 from an inhibitor to an efficient substrate in vitro and redistributes equivalent Bazooka mutants to AJs in vivo. These data are consistent with high-affinity inhibitory interactions between Par3/Baz and aPKC preventing Par3/Baz phosphorylation and thereby promoting stable complex formation and apical localization. Modulation of the CR3 inhibitory arm by phosphorylation or engagement of the aPKC pocket by partner proteins would switch Par3/Baz to a more transient type of interaction, consequently enabling efficient phosphorylation of Par3/Baz by aPKC and subsequent relocalization to AJs.

## Results

### The Par3 CR3 Region Inhibits Nucleotide-Bound Primed PKCι Kinase Domain through Two Flanking Arm Contacts

**Figure 1:**
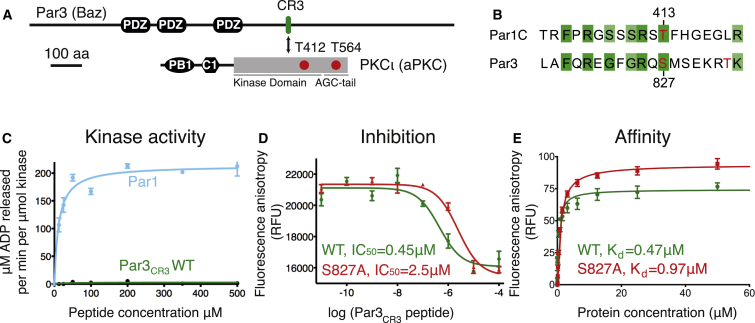
Par3/Baz CR3-Mediated Inhibition of aPKC In Vitro (A) Domain structure of Par3 and PKCι and location of key phosphorylation sites in each. For more detail on aPKC and Par3/Baz subcellular localization and how aPKC phosphorylation of Par3/Baz switches Par3/Baz localization from the apical membrane to AJs, see Figure S1. (B) Sequence alignment of human Par3 CR3 region with Par1 highlighting known phosphorylation sites (red). (C) Par3_CR3_ inhibits PKCι_KD_-2P catalytic activity in an in vitro kinase assay, whereas a Par1-derived peptide is a substrate. (D) The IC_50_ curves for Par3_CR3_. (E) Affinity of fluorescein-labeled Par3_CR3_ for PKCι_KD_-2P measured by fluorescence anisotropy. RFU, relative fluorescence units; WT, wild-type. Data are plotted as mean ± SEM. See also Figure S2 for purification and further characterization of PKCι_KD_-2P.

The human Par3 conserved region 3 (CR3, covering residues 816–834, defined hereafter as Par3_CR3_) is able to bind to PKCι (Nagai-Tamai et al., 2002) and contains a phospho-acceptor site (P site) at residue serine 827 known to be phosphorylated by PKCι (Figures 1A and 1B). To characterize its interaction with PKCι we purified a “primed” active form of the human PKC-iota kinase domain (referred to as PKCι_KD_-2P) and a partially primed low-activity form (referred to as PKCι_KD_-1P), referring to the status of the two “priming” phosphorylation sites at pT412 and pT564 (Figures 1A and S2A–S2C). We then probed how efficiently they were able to phosphorylate Par3_CR3._ Surprisingly, we found that Par3_CR3_ strongly inhibited the catalytic activity of PKCι_KD_-2P in vitro and could competitively block phosphorylation of a model substrate peptide, with an apparent 50% inhibitory concentration (IC_50_) of 0.45 ± 0.18 μM. In contrast, peptides from other known aPKC substrates such as Par1 were efficiently phosphorylated and were unable to inhibit (Figures 1B–1D). Using a fluorescence anisotropy assay, we found that the Par3_CR3_ binds to PKCι_KD_-2P with submicromolar affinity (K_D_ of 0.47 ± 0.09 μM), as does an S827A mutant (K_D_ of 0.97 ± 0.07 μM) (Figure 1E). PKCι_KD_-2P is a good surrogate for an activated Par complex containing Par6-PKCι-Cdc42 complex that exhibits high activity in vitro and is also potently inhibited by Par3_CR3_ (data not shown). In contrast, PKCι_KD_-1P was not inhibited to the same extent and had a much lower affinity for Par3_CR3_ (compare Figures 1D, S2D, and S2E). We conclude that a high-affinity Par3_CR3_ targets PKCι_KD_-2P and inhibits its catalytic activity.

**Figure 2:**
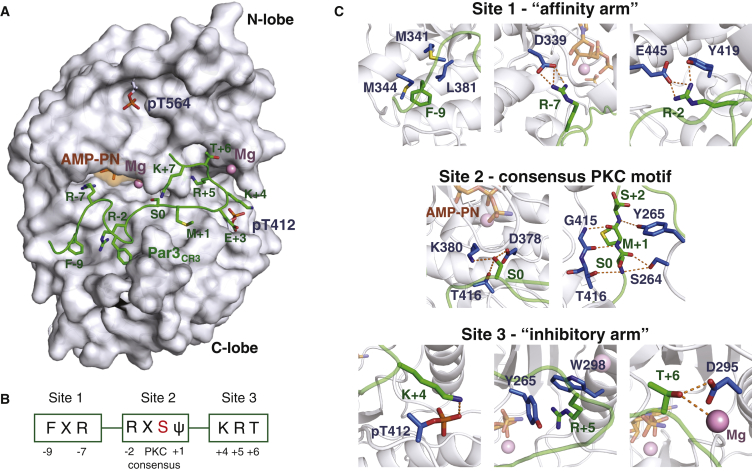
Structural Basis for Par3/Baz CR3-Mediated Inhibition of aPKC (A) Overall structure of PKCι_KD_-2P (gray surface) bound to Par3_CR3_ (green stick) with Mg-AMP-PNP shown as an orange surface and priming sites at T564 and T412 indicated. (B) Schematic representation of three sites of contacts between Par3_CR3_ and PKCι_KD_-2P. The known phospho-acceptor site at serine 0 is shown in red. (C) Close up of the contacts between Par3_CR3_ peptide (green) and PKCι_KD_-2P (residues making contacts shown in blue). Hydrogen bonds between side chains or main-chain atoms are shown as dashed red lines. Pink spheres represent magnesium ions. See also Figures S3A and S3B for refined Par3_CR3_ electron density and intramolecular hydrogen bonds within Par3_CR3_.

To understand how Par3_CR3_ could inhibit PKCι_KD_-2P, we determined the 2.0-Å crystal structure of a longer Par3 peptide (residues 816–841) bound to PKCι_KD_-2P and Mg-AMPPNP (adenylyl imidodiphosphate) (Figures 2A and S3A; Table 1). The Par3_CR3_ peptide is well ordered in this structure and contains seven intramolecular hydrogen bonds (Figure S3B). It engages PKCι_KD_-2P by adopting a “staple”-shaped conformation with two arms that flank the S827^Par3^ phospho-acceptor site. Each arm binds in close proximity to opposite ends of the nucleotide, suggesting that recognition of aPKC is driven by nucleotide occupancy. The relative orientation of N and C lobes indicates a “closed” rather than “open” conformation. Par3_CR3_ contacts extend from a pocket beneath the ribose-binding pocket of PKCι (site 1), across the G helix (site 2) through to the activation loop, αB and αC helices of the PKCι_KD_-2P N lobe (site 3) (Figures 2A and 2B). A total surface area of more than 1,305 Å^2^ is buried within the complex, consistent with a high-affinity inhibitory interaction. The nucleotide cleft is occupied by an Mg-AMPPNP nucleotide (Figure 2C). The conserved nucleotide-coordinating lysine (K283^PKCι^) forms a salt bridge with the conserved αC-helix glutamate (E302^PKCι^) side chain found in many active kinase conformers (Kornev et al., 2008). The terminal γ-phosphate of AMPPNP is not observed in the structure, consistent with AMPPNP being rapidly hydrolyzed under the crystallization conditions (see Experimental Procedures). A magnesium ion, equivalent to Mg2 of PKA, is present, bridging both the α and β phosphates of AMPPNP (Adams and Taylor, 1993, Zheng et al., 1993). The Mg1 ion is not present, as frequently found in ADP-complexed AGC kinase structures.

The amino-terminal part of Par3_CR3_ binds to site 1 (PKCι_KD_-2P kinase C lobe) through an F-X-R motif at positions −9 (F−9) and −7 (R−7) defined relative to the phospho-acceptor (P site) at serine 0 (equivalent to S827 of human Par3). F−9 lies deep within a hydrophobic cleft formed by M341^PKCι^, M344^PKCι^, and L381^PKCι^ beneath the nucleotide pocket (Figure 2C). In addition, the side chain of R−7 forms a salt bridge to D339^PKCι^, just beneath the ribose ring of the AMPPNP, while that of R−2 engages conserved residues Y419^PKCι^ and E445^PKCι^ (Figure 2C). As this motif does not appear to directly perturb aPKC catalytic residues, we refer to this element hereafter as the “affinity arm” of Par3_CR3_ (Figure 2C).

From site 1, the Par3_CR3_ backbone adopts two consecutive type II reverse turns with positive phi-main-chain angles at E−6 and G−3. This leads into site 2, positioned to contact the G helix through residue F−4 that displaces and disorders the aPKC-specific kinase insert (residues 455^PKCι^ to 466^PKCι^). The phospho-acceptor serine-0 hydroxyl hydrogen bonds to side chains of D378^PKCι^, K380^PKCι^, and T416^PKCι^, preventing a catalytically competent orientation for nucleophilic attack on the ATP γ-phosphate. Glycine-rich loop residues S264^PKCι^ and Y265^PKCι^ side chains contact the CR3 main-chain atoms near the P site, as does the activation-loop main chain, to orient the Par3_CR3_ peptide and position the M+1 side chain into the known P+1 AGC kinase hydrophobic pocket (Figure 2C) (Pearce et al., 2010).

**Figure 3:**
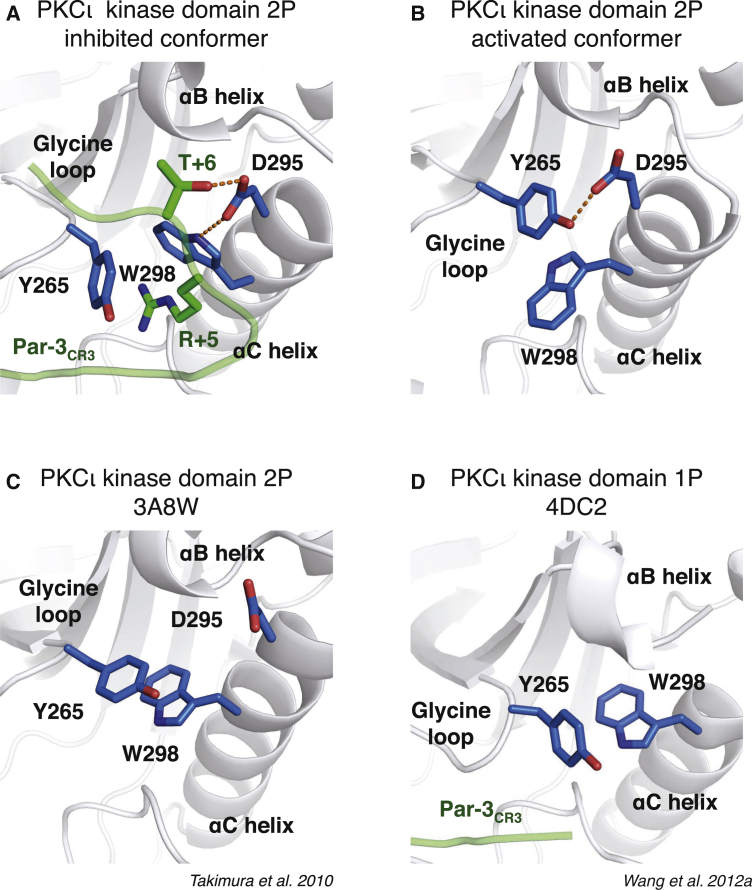
Close-Up View of the Par3 CR3 Inhibitory Arm Pocket Bound to PKCι_KD_-2P with Other PKCι_KD_ Structures (A) Close up of the inhibitory R+5 hook of Par3_CR3_ clamped by side chains Y265^PKCι^ (P loop) and W298^PKCι^ (αC helix) of PKCι_KD_-2P (gray cartoon, major interaction residues are shown as blue sticks). Key structural features of the PKCι_KD_-2P are labeled. Hydrogen bonds between key side chains are shown as dashed red lines. (B) Close up of R+5 pocket in the active conformation of AMPPCP-bound PKCι_KD_-2P structure. Hydrogen bonds between key side chains are shown as dashed red lines. (C) Close up of R+5 pocket in the previously solved ATP-bound PKCι_KD_-2P structure (PDB: 3A8W) (Takimura et al., 2010). (D) Close up of PKCι_KD_-1P K283R mutant within its ATP cleft (PDB: 4DC2) (Wang et al., 2012a). See also Figure S3C for refined nucleotide analog electron density and Figure S3D for a comparison with a chemical inhibitor-induced PKCι_KD_-2P conformer.

Site 3 contains carboxy-terminal flanking residues to the P site stretching from S+2 to T+6. We define this portion of Par3_CR3_ as the “inhibitory arm,” as it directly perturbs an active PKCι_KD_-2P N-lobe conformation (discussed later). Residue K+4 directly contacts pT412^PKCι^ of the activation loop enhancing the recognition of mature, primed PKC ι_KD_-2P, but importantly not a partially primed PKCι_KD_-1P. Crucially, the R+5 side chain is buried within a hydrophobic pocket beneath the regulatory αC helix. The pocket is lined by side chains from Y265^PKCι^ on the glycine loop and W298^PKCι^ of the αC helix, each making π-stacking interactions with the guanidino group of R+5 (Figures 2C and 3A). Both aromatic side chains are unique to aPKC isozymes from *Drosophila* to mammals. Finally, T+6 (equivalent to T833^Par3^, a known ROCK-driven phosphorylation site discussed later) lies adjacent to an acidic patch within the αB helix making side-chain and main-chain contacts to D295^PKCι^ and a Mg ion (Figures 2C and 3A). Overall, the structure reveals that the Par3_CR3_ clamp involves an “inhibitory arm” and an “anchoring arm,” which together recognize and inhibit a nucleotide-bound PKCι_KD_-2P conformer.

### Comparison of Par3_CR3_-Inhibited PKCι Complex with an Active PKCι Conformer Reveals the Basis for Inhibition

To fully understand how Par3_CR3_ inhibits PKCι and disrupts its activated state, we determined the structure of an “active” conformer of PKCι for comparison (Figure 3B). Previous structures of PKCι kinase domain (PDB: 3A8W and 4DC2) (Takimura et al., 2010, Wang et al., 2012a) exhibited either a disordered or displaced αB-αC loop (Figures 3C and 3D). We captured an active mature PKCι conformation bound to the ATP analog 5′-(β,γ-adenylyl methylene)diphosphonate (AMPPCP) at 1.8 Å (Figure S3C and Table 1). This analog was resistant to hydrolysis compared with AMPPNP. The structure has an ordered αB-αC loop and reveals side-chain contacts between Y265^PKCι^ of the P loop and D295^PKCι^ of the αB-αC loop. This interaction stabilizes Y265^PKCι^ side-chain stacking with a rotamer of W298^PKCι^ from the αC helix (Figure 3B). Other PKC isoform structures have a phenylalanine and cysteine, respectively, at these positions (Grodsky et al., 2006, Leonard et al., 2011, Xu et al., 2004). Structural comparisons suggest that Par3_CR3_ inhibitory arm not only separates P-loop contacts with αB-αC loop/αC helix but also hijacks Y265^PKCι^ and S264^PKCι^ side chains to directly form hydrogen bonds with CR3 main-chain atoms. Comparing the Par3_CR3_ inhibitory complex with 1ATP (PKA bound to Mg-ATP and PKI peptide) suggests that the R+5 side-chain guanidine group lies close to the Mg2 ion of an active kinase conformation (Adams and Taylor, 1993, Zheng et al., 1993), indicating another layer of Par3_CR3_ disruption of an active PKCι conformation. Furthermore, T+6 (equivalent to T833^Par3^), which makes direct contact with D295^PKCι^, is a phospho-acceptor site targeted by the ROCK kinase, leading to a disruption of PKCι interaction with Par3 (Nakayama et al., 2008). This would predict, based on our structural comparison, that modulation of the “inhibitory arm” of Par3_CR3_ by ROCK kinase phosphorylation, or inaccessibility of the pocket to which it binds, could influence whether Par3 can inhibit PKCι or engages it as a substrate.

### A Shared High-Affinity Anchor Motif Used by aPKC Substrates and Inhibitors

**Figure 4:**
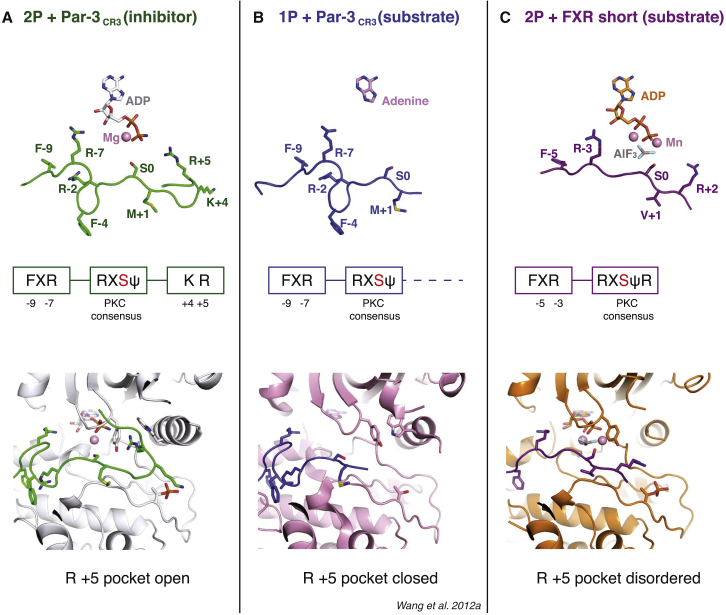
Structural Comparison of aPKC-Substrate and aPKC-Inhibitor Interactions (A) Top panel: cartoon depiction of the Par3_CR3_ (green) bound to PKCι_KD_-2P (omitted for clarity) highlighting the position of inhibitory −7 and +5 arginine residues flanking the nucleotide (gray sticks). Middle panel: schematic representation of the interaction sites of Par3_CR3_ with PKCι_KD_-2P. Bottom panel: close up of the R+5 pocket occupied by Par3_CR3_ and the Y265 and W298 clamp residues of PKCι_KD_-2P (gray). The known phospho-acceptor site at serine 0 is shown in red for all panels. (B) Top panel: cartoon depiction of the Par3_CR3_ (blue) bound to PKCι_KD_-1P K283R mutant (omitted for clarity) from Wang et al. (2012a) lacking inhibitory site 3 contacts, possibly reflecting a lower-affinity substrate-type interaction. Middle panel: schematic representation of the interaction sites of Par3_CR3_ with PKCι_KD_-1P K283R mutant. Bottom panel: close up of the “closed” R+5 pocket in which the clamp residues Y265 and W298 make direct contact (PKCι_KD_-1P shown in pink). (C) Top panel: cartoon depiction of the FXR_short_ (purple) bound to PKCι_KD_-2P, Mn-ADP, and AlF_3_. This artificial substrate lacks inhibitory site 3 contacts but shares site 1 FXR motif. Middle panel: schematic representation of the interaction sites of FXR_short_ with PKCι_KD_-2P. Bottom panel: close up of the ordered portion of the R+5 pocket including the glycine loop and Y265 but not the disordered W298 from the αC helix (PKCι_KD_-1P shown in orange). See also Figures S4A–S4D for the design and characterization of FXR_short_ peptide. See Figures S4E and S4F for refined FXR_short_ peptide electron density and a superposition of the Par3_CR3_ and FXR_short_ peptides bound to PKCι_KD_-2P.

Our Par3_CR3_-PKCι_KD_-2P inhibitory complex differs significantly from a previous structure of an ATP-binding deficient and partially primed PKCι K283R mutant (PKCι_KD_-1P, PDB: 4DC2) bound to Par3_CR3_ (Wang et al., 2012a). In the absence of nucleotide, Par3_CR3_ residues +3 to +7 were disordered and, therefore, the CR3 region is lacking inhibitory site 3 contacts (Wang et al., 2012a). Consistent with this, Par3_CR3_ is unable to potently inhibit the partially primed PKCι_KD_-1P or bind with high affinity (Figures S2D, S2E, and S4B). We present evidence that our Par3_CR3_-PKCι_KD_-2P structure represents a Par3_CR3_-mediated inhibitory complex of mature PKCι. However, the structure reported by Wang et al. (2012a) most likely resembles a weaker and transient Par3-PKCι interaction relevant to a protein kinase-substrate interaction (Figure 4).

To explore and capture a substrate peptide bound to PKCι_KD_-2P, we used an artificial substrate (FKRQGSVRRR, referred to hereafter as F-X-R_short_ peptide) (Figures S4A–S4E). This efficient PKCι substrate closely resembles the aPKC consensus motif identified from screening randomly oriented peptide libraries by Cantley and co-workers (Nishikawa et al., 1997). We therefore determined a crystal structure for F-X-R_short_ bound to PKCι_KD_-2P in the presence of Mn-ADP and AlF_3_, a transition-state analog (Figure S4E). Manganese ions corresponding to Mg1 and Mg2 ions are present in the structure, and the AlF_3_ is positioned as expected to mimic the transition state for the γ-phosphate. Surprisingly, the structure revealed that the F-X-R motif at F−5 and R−3 engages precisely the same site 1 residue contacts (M341^PKCι^, M344^PKCι^ and L381^PKCι^, and D339^PKCι^) as the F−9 and R−7 contacts used by Par3_CR3_, despite their different position in the primary sequence (Figure S4F). Moreover, the R−3 side chain directly makes a hydrogen bond with the ribose hydroxyl, perhaps sensing nucleotide occupancy. We also observe an R+2 side-chain bridging contact between phospho-T412^PKCι^ and G398^PKCι^ main-chain carbonyl, making two key hydrogen bonds with these groups. We note that many aPKC substrates have an R+2 side chain, suggesting that direct contact with a phosphorylated activation loop may reflect a common interaction made by aPKC substrates.

From the Par3_CR3_ structure, it is evident that the F-X-R_short_ peptide does not inhibit PKCι because it lacks a C-terminal inhibitory motif (Figures 4 and S5). Consistent with this, the Par1 peptide characterized as a good aPKC substrate also has an F-X-R anchor and a validated aPKC phosphorylation site, but lacks an obvious inhibitory motif (Figure 1C) (Hurov et al., 2004). In contrast, a Kibra-derived peptide (residues 919–978) containing a validated aPKC phosphorylation site has both an F-X-R motif anchor and a K-R inhibitory motif. As such it is able to potently inhibit PKCι in vitro (Figures S5A–S5C), consistent with reports of Kibra inhibiting aPKC kinase activity in epithelial cells (Yoshihama et al., 2011). Indeed a related peptide from WWC2 protein, a poorly characterized Kibra homolog, also inhibits PKCι in vitro (Figures S5A–S5C). Taken together, these data indicate that an F-X-R motif anchor amino-terminal to an aPKC phosphorylation site can be found in both aPKC substrates and inhibitors at variable lengths in their primary sequence from the phospho-acceptor site. Furthermore, the C-terminal inhibitory arm bearing a K-R-T motif is unique to aPKC protein inhibitors such as Par3 and Kibra, and can be predictive of an inhibitory function (WWC2).

### Manipulating Par3 CR3 Flanking Arms In Vitro Switches Par3 from an Inhibitor to an Efficient PKCι Substrate

**Figure 5:**
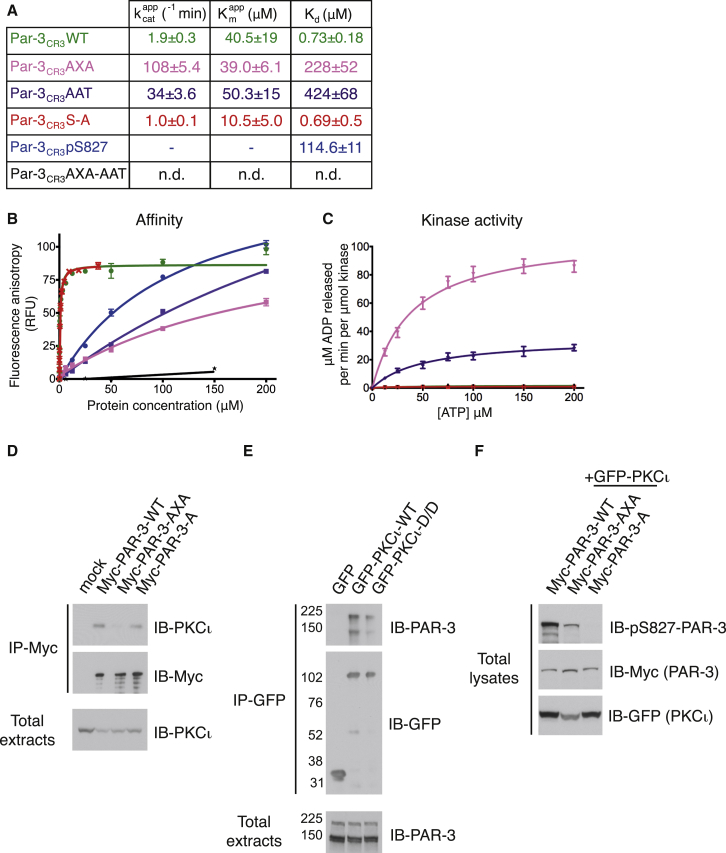
Reducing the Par3_CR3_ Affinity for PKCι_KD_-2P Promotes Efficient CR3 Phosphorylation In Vitro (A) Summary table of k_cat_, K_D_, and K_M_ constants between various Par3_CR3_ mutants and PKCι_KD_-2P. Data are presented as mean ± SEM. n.d., not determined. (B) Binding curves for Par3_CR3_ and various Par3_CR3_ mutants determined by fluorescence polarization (color coded as in A). (C) PKCι_KD_-2P catalytic activity kinetic rate constants for Par3_CR3_ and various Par3_CR3_ mutants (color coded as in A). For further details of other inhibitory peptides similar to Par3_CR3_, see Figure S5. (D) Co-immunoprecipitation (IP) of full-length Myc-Par3 or mutants (Par3-A-X-A and Par3-A (S827A)) and GFP-PKCι from HCT-116 cells shows that the F-X-R to A-X-A mutation dramatically reduces the interaction. (E) Co-immunoprecipitation of GFP-PKCι or GFP-PKCι-D/D with Myc-Par3 also severely impairs the interaction. GFP-PKCι-D/D is a mutant replacing residues D330/D373 that interact with the F-X-R motif by alanine. (F) Immunoblot (IB) using a phospho-S827-specific antibody indicates that Par3 and Par3 A-X-A mutant (but not Par3-A) are phosphorylated in HCT-116 cells. For details showing evidence of phosphorylation of A-X-A Baz mutant, see Figure S6.

Our results suggested that Par3 CR3 arms flanking the consensus PKC phosphorylation site cooperate to inhibit PKCι. To probe the individual contributions of each arm, we characterized Par3_CR3_ substitutions at critical contact residues in the affinity arm and the inhibitory arm for their impact on Par3_CR3_ affinity for PKCι and ability inhibit kinase activity. Two mutants were prepared: first, substitution of F-Q-R to A-Q-A in the site 1 affinity arm, referred to as A-X-A hereafter; and second, substitution of K-R-T to A-A-T of the site 3 inhibitory arm, referred to as A-A-T. Consistent with our crystal structure, either A-X-A or A-A-T mutation within Par3_CR3_ markedly reduce the CR3-binding affinity for PKCι_KD_-2P, but without abolishing the interaction entirely (Figures 5A and 5B). A phospho-S827^Par3^ peptide representing the PKCι reaction product bound poorly, with affinity two orders of magnitude lower than in Par3_CR3_ (Figures 5A and 5B).

Surprisingly, the in vitro kinase assay demonstrated that either an A-X-A or A-A-T mutation gave a substantial increase in Par3_CR3_ phosphorylation by PKCι_KD_-2P, greatly enhancing the apparent k_cat_ values (Figures 5A and 5C). The large effects observed for each mutant (57-fold for A-X-A Par3_CR3_ versus 18-fold A-A-T Par3_CR3_) suggest that these substitutions uncouple the ability of Par3_CR3_ to inhibit PKCι_KD_-2P, resulting in access to the PKC consensus site at S827^Par3^ and efficient phosphorylation by PKCι_KD_-2P. The magnitude of the increased k_cat_ values allowed the measurement of a K_M_ for the A-X-A Par3_CR3_ substrate (K_M_ of 39 μM), which was not possible for wild-type Par3_CR3_. Combining both site 1 and site 3 mutations (A-X-A + A-A-T) within Par3_CR3_ generated a very poor substrate that was not detectably phosphorylated and had no measurable interaction with PKCι_KD_-2P (data not shown). These data indicate that while mutating either the anchoring arm or inhibitory arm switches Par3_CR3_ to an efficient aPKC substrate, the remaining arm must contribute sufficient binding affinity (both are basophilic) as mutating both arms generates a Par3_CR3_ that is neither a substrate nor an inhibitor. These striking results are also consistent with the notion that tight inhibitory binding of Par3_CR3_ to PKCι_KD_-2P must prevent its phosphorylation while weaker binding without the inhibitory interactions exposes its PKC site, switching it to a highly efficient in vitro PKCι substrate.

To validate some aspects of these results, using full-length PKCι and Par3 in cells we undertook co-immunoprecipitation experiments of differentially tagged full-length forms of PKCι and Par3 expressed in transiently transfected HCT-116 cells. Endogenous PKCι was efficiently immunoprecipitated through exogenous wild-type Par3, while a full-length human Par3 bearing the site 1 A-X-A mutation showed substantially reduced interaction with PKCι (Figure 5D), consistent with in vitro data for the isolated CR3 domain. Note that endogenous PKCι retains binding to the non-phosphorylatable Par3-S827A (Myc-PAR-3-A) similarly to the wild-type but is unable to be turned over and remains tightly associated with PKCι. Reciprocal co-immunoprecipitation of overexpressed exogenous wild-type GFP-PKCι efficiently pulled down endogenous Par3, whereas mutating residues D339^PKCι^/D382^PKCι^ (GFP-PKCι-D/D) that directly contact the R−7 side chain of Par3 also markedly reduced the Par3-PKCι interaction (Figure 5E). Arm contacts identified from the crystal structure are therefore necessary for Par3 interaction with PKCι. We developed a specific phospho-Par3 antibody to probe whether mutation of the A-X-A arm abolished interaction with PKCι completely as well as PKCι-mediated phosphorylation. While the A-X-A is less phosphorylated compared with wild-type (Figure 5F), we noted a large increase in ubiquitinylated Par3 (under conditions of proteasome inhibition), a likely consequence of Par3 phosphorylation in non-polarized cells (data not shown). Taken together, immunocomplex recovery from HCT-116 cells confirmed that (1) the contacts observed structurally indeed influence interaction in cells as predicted, and (2) “weakening” the strength of the aPKCι-Par3 interaction through site-specific mutation prevents Par3 inhibition, leading instead to Par3 phosphorylation.

### Apical-Junctional Polarization of Par3/Baz In Vivo Is a Consequence of Switching between Inhibitory and Substrate-Binding Modes

**Figure 6:**
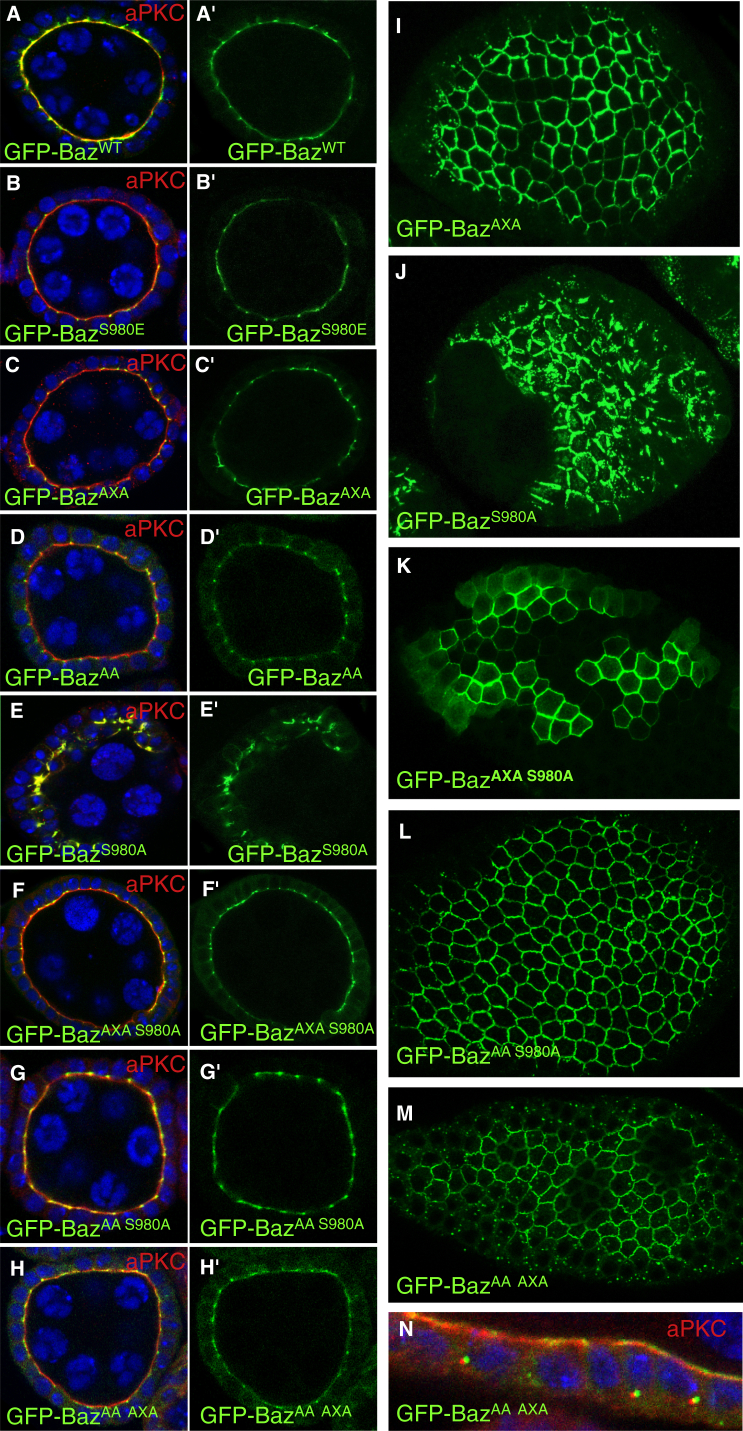
Switching Par3/Baz from Apical to Junctional In Vivo (A) GFP-tagged Par3/Baz (green) localizes to the apical domain (marked by aPKC in red) and also to AJs. (B) Phosphomimic GFP-tagged Par3/Baz S980E (green) is largely excluded from the apical domain (marked by aPKC in red) and localizes to AJs. (C) Low-affinity GFP-tagged Par3/Baz F-X-R to A-X-A mutant (green) is largely excluded from the apical domain (marked by aPKC in red) and localizes to AJs. (D) Low-affinity GFP-tagged Par3/Baz K-H to A-A mutant (green) is largely excluded from the apical domain (marked by aPKC in red) and localizes to AJs. (E) Phospho-mutant GFP-tagged Par3/Baz S980A mutant (green) co-localizes apically with aPKC (red) and also partially disrupts cell polarity, consistent with its inhibitory function. See also Figure S7 for evidence that Baz co-localizes with aPKC in the absence of kinase activity. (F) Phospho-mutant GFP-tagged Par3/Baz S980A that also carries the F-X-R to A-X-A mutation (green) fails to co-localize with aPKC (red) and instead localizes to AJs. (G) Phospho-mutant GFP-tagged Par3/Baz S980A that also carries the K-H to A-A mutation (green) fails to co-localize with aPKC (red) and instead localizes to AJs. (H) The double mutant K-H to A-A and F-X-R to A-X-A localizes primarily to AJs. (I) Apical section of GFP-BazAXA expressing follicle cell epithelium, showing junctional localization. (J–M) Apical section of GFP-BazS980A (J) expressing follicle cell epithelium, showing mislocalization to the apical surface. Apical sections of (K) GFP-BazAXA S980A-, (L) GFP-BazAA S980A-, and (M) GFP-BazAA AXA-expressing follicle cell epithelium, showing restoration of junctional localization. (N) Non-overlapping punctate localization of GFP-BazAXA AA (green) with aPKC (red). DAPI staining is shown in blue in (A)–(H) and (N). GFP-tagged Par3/Baz is shown in (A′)–(H′).

If the affinity of the Par3/Baz-aPKC interaction essentially determines the localization of Par3/Baz in epithelial cells, then the Par3_CR3_ substitutions within each arm (A-X-A or A-A-T mutants) characterized in vitro should affect the localization of Par3/Baz in vivo. To test this prediction, we mutated the CR3 region of full-length GFP-tagged *Drosophila* Baz in the F-X-R motif to A-X-A or the K-H-T motif to A-A-T and examined their apical domain or AJ localization in vivo. In the follicular epithelium, GFP-tagged wild-type Baz (GFP-Baz) co-localizes with aPKC at the apical membrane and also localizes to AJs (Figure 6A). Phospho-Baz is known to localize to AJs (Morais-de-Sa et al., 2010), and a GFP-tagged phosphomimic version of Baz (GFP-Baz S980E) expectedly fails to co-localize with aPKC at the apical membrane but instead localizes to AJs (Figure 6B) (Morais-de-Sa et al., 2010, Walther and Pichaud, 2010). Both the GFP-Baz A-X-A and A-A-T mutant localize similarly to the phospho-mimetic (Figures 6C, 6D, and 6I), consistent with the view that lowering affinity (as observed in cells; Figure 5B) and/or removing inhibitory elements from CR3 induces phosphorylation of Baz (as observed in vitro; Figure 5) and therefore results in its localization to AJs rather than stable Par complex formation at the apical membrane.

It was important to distinguish between whether the relocalization of the Baz A-X-A or A-A-T mutants was due to exposure of the S980 site and phosphorylation as shown in vitro for Par3_CR3_, or simply due to a lack of interaction with aPKC. A combined A-X-A + A-A-T site mutation in vitro showed a complete loss of interaction of CR3 with PKCι and no phosphorylation of serine 827 (Figure 5A). An equivalent GFP-Baz A-X-A + A-A-T mutant also localized to AJs (Figures 6H, 6H′, and 6M). Interestingly, this combined mutant showed distinct intracellular puncta in which the Baz-GFP mutant no longer overlapped with aPKC, suggesting that both proteins are mutually exclusive on the same membrane (Figure 6N).

We then sought to verify whether the A-X-A Baz mutant was indeed phosphorylated in *Drosophila* cells. Available phospho-antibodies against Par3 S827 and Baz S980 were previously raised against an epitope that included the F-X-R motif and therefore could not detect the Baz A-X-A mutant or its phosphorylation status (data not shown). Efforts to raise a Baz phospho-antibody against S980 peptides excluding the F-X-R motif were not successful. Therefore, we verified that the A-X-A Baz mutant was phosphorylated in *Drosophila* cells, by preparing transfected S2 cell extracts containing wild-type or A-X-A mutant Baz and probed S980^Baz^ phosphorylation status using dimethyl labeling and mass spectrometry. Previous efforts to identify the Baz_CR3_ phospho-site in wild-type and A-X-A mutant contexts using trypsin digest were unsuccessful due to cleavage at R979 and K984, yielding very short peptides. Therefore, Baz_CR3_ samples were first treated by in-gel reductive dimethylation, to generate the Baz_CR3_ peptide spanning the sequence (phospho)SISE(me2K)HHAALDAR. The dimethylation reaction modifies lysine ɛ-amino groups, thereby greatly reducing the ability of trypsin to cleave after lysines. This allowed capture of the phospho-Baz_CR3_ peptide, facilitated quantification of chromatographic peak areas, and identified phospho-peptides. The forward sample reaction used heavy (CD_2_O with wild-type Baz mutant) or light (CH_2_O with A-X-A Baz) reagents, resulting in a mass difference of 12 Da and an *m*/*z* difference of 4 for the triply charged target peptide (Figures S6A and S6B). The reverse sample used heavy (CD_2_O with A-X-A Baz mutant) or light (CH_2_O with wild-type Baz) reagents, and two control peptides were also used to assess any differences in peptide recovery from the SDS-PAGE gel (Figures S6A and S6C). Recovery was poorer for all heavy-labeled reverse samples including both controls, although the data clearly showed that both wild-type and A-X-A Baz proteins were phosphorylated at S980^Baz^ (Figures S6C and S6D). Taken together, our data suggest that the A-X-A mutant can be phosphorylated by aPKC in vitro in HCT-116 and S2 cells. Moreover, it can be distinguished from the A-X-A + A-A-T combined mutant that is no longer a substrate for aPKC and fails to interact with it both in vitro and in vivo.

We next tested the idea that phosphorylation of Par3/Baz controls its localization simply by feeding back to block its binding to aPKC (as observed for phospho-Par3_CR3_ in vitro; Figure 5). A GFP-tagged phospho-mutant form of Baz (GFP-Baz S980A) is known to fail to localize to junctions and instead co-localizes perfectly with aPKC (Figures 6E, 6J, and S7) (Morais-de-Sa et al., 2010, Walther and Pichaud, 2010). We find that expression of this construct also disrupts cellular morphology, consistent with previously reported data (Morais-de-Sa et al., 2010). If the localization and morphology phenotypes of GFP-Baz S980A are caused by tight inhibitory binding to aPKC, it should be possible to reverse these phenotypes by introducing either the F-X-R or K-H-T site mutation to lower the affinity of this interaction. Accordingly, we find that GFP-Baz A-X-A or A-A-T S980A double mutants fail to co-localize with aPKC and instead localize to AJs and do not show polarity defects (Figures 6F–6L). These results strongly support the view that phosphorylation of Par3/Baz controls its localization through lowering its binding affinity for aPKC, because the phenotypic consequence of loss of phosphorylation can be reversed by mutations that reduce affinity. Consistent with our in vitro data, we propose that access to the phosphorylation site within Par3/Baz is in turn controlled by modulation of the high-affinity and inhibitory arms of the CR3 region.

## Discussion

**Figure 7:**
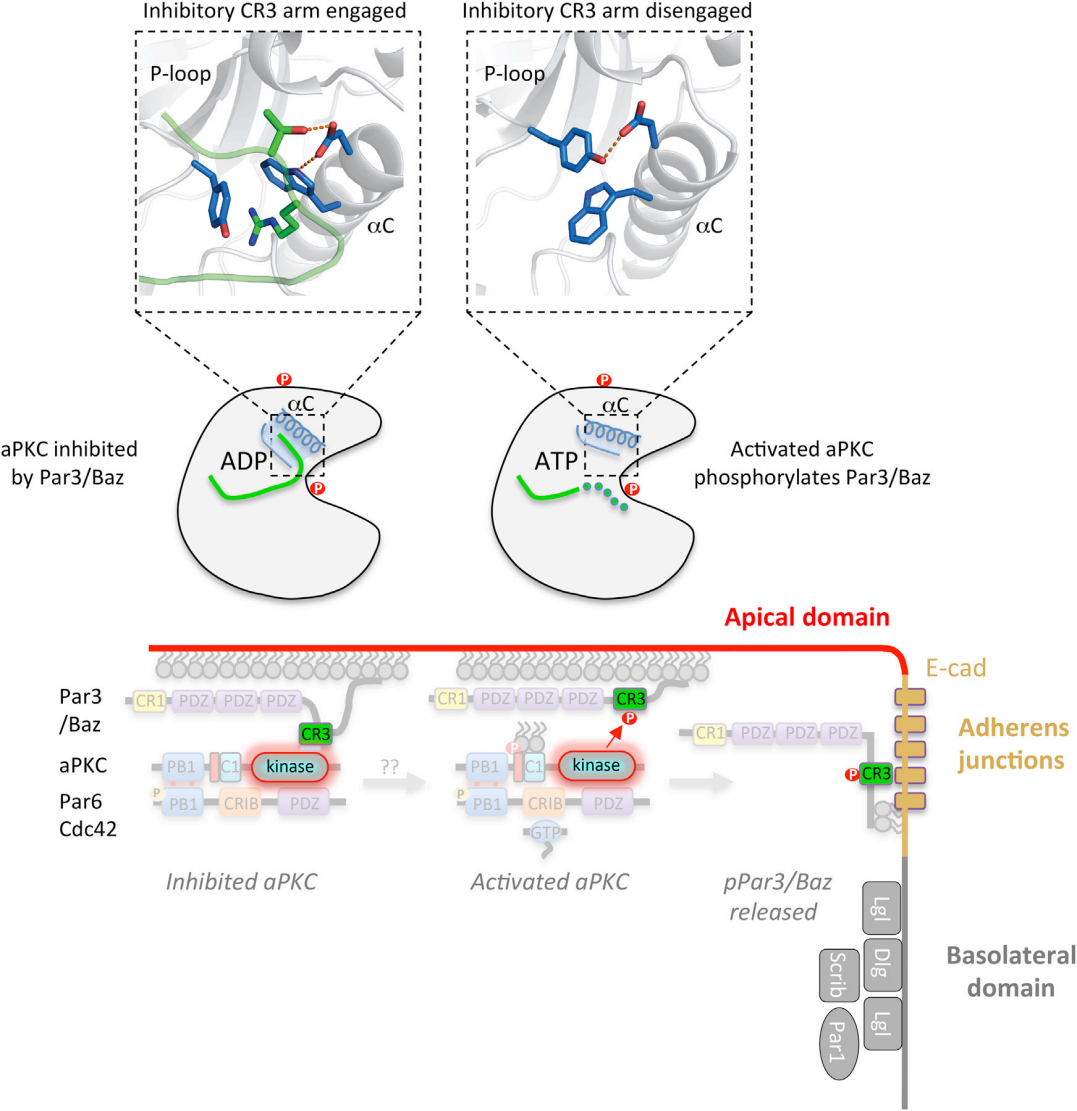
Proposed Model for Par3/Baz Antagonism and Polarization We propose two states for PKC-Par3/Baz interaction driven by aPKC kinase domain and Par3/Baz CR3 region. A high-affinity interaction is inhibitory and requires both arms flanking the PKC consensus motif of the CR3 region. By engaging pockets within both the N and C lobes of aPKC kinase domain, Par3/Baz phosphorylation is prevented but Par6-aPKC is recruited to the apical membrane. In an activated state, aPKC is resistant to CR3 antagonism due either to an inaccessible αC helix pocket or a CR3 interaction being destabilized by phosphorylation of T833^Par3^ or by aPKC lacking a PDK1-driven T412^PKCι^ phosphorylation. Either of these possibilities could result in Par3/Baz binding as a substrate exposing its PKC consensus site R-X-S-Ψ to phospho-transfer. Phosphorylated Par3/Baz is then excluded from the Par complex and thus from the apical membrane domain, and relocalizes to AJs.

Our results reveal the molecular basis for Par3 antagonism of aPKC through high-affinity inhibitory CR3 arm interactions that span both N and C lobes of the PKCι kinase domain. Our structural and biochemical data provide a model that supports a mechanism explaining apical-junctional polarization of Par3/Baz in epithelial cells (Figure 7). Previous work has shown that apical localization of Par3/Baz depends on it being part of the Par complex, where Par3/Baz is not phosphorylated by aPKC, while junctional localization of Par3/Baz occurs when it is phosphorylated by aPKC (Morais-de-Sa et al., 2010, Walther and Pichaud, 2010). Formation of the Par complex with Par3/Baz is known to be crucial for apical membrane recruitment of aPKC-Par6 (Gao et al., 2002).

Here we provide an explanation for why Par3/Baz is not phosphorylated while engaged within the Par complex even though it can be phosphorylated when it separates from the Par complex. Our crystal structure of the Par3 CR3–PKCι kinase domain interaction reveals the basis for high-affinity Par3_CR3_ contacts through the coordinated action of two short motifs flanking the PKC consensus motif R-X-S827-Ψ. Together these motifs cooperate to inhibit aPKC, one bringing high affinity and the other enabling inhibitory contacts. The observation that the same N-lobe pocket is closed in structures of partially primed PKCι_KD_-1P with a peptide resembling an aPKC-substrate interaction supports a second mode of engagement of Par3. Access to this N-lobe pocket may dictate whether aPKC-interacting proteins with an R+5 hook can inhibit aPKC or are phosphorylated as substrates. The location of the pocket adjacent to the αC helix and the aPKC activation loop suggests a potential mechanism to regulate the decision to engage and phosphorylate or to be sensitized to Par3 CR3 inhibition in the case of a fully primed active aPKC conformer.

The precise mechanism determining this switch requires further study and is beyond the scope of these investigations. Potential regulatory influences could include the availability of Par3 inhibitory arms, competition with other aPKC substrates, the presence of binding partners adjacent to the aPKC αC helix, post-translational modifications of the Par3_CR3_ region (such as T833^Par3^ phosphorylation by the ROCK kinase), or even regulation of the aPKC activation loop (by PDK1 or by dephosphorylation by an unknown A-loop phosphatase). One or more of these factors, depending on the physiopathological status of the epithelium, could affect the Par3-binding mode. We do note that Kibra (and its homolog WWC2) contains an F-X-R motif and a K-R-T hook flanking its known aPKC phosphorylation site (between residues 911 and 978), suggesting that it too could act as dual-action inhibitor/substrate, consistent with biochemical data (Figure S5) (Yoshihama et al., 2011). Similarly, many known aPKC substrates have an adjacent F-X-R motif, for example, Par1, Par2, and ROCK kinase, suggesting that the F-X-R may provide specificity and an affinity boost to these validated aPKC substrates (Hurov et al., 2004, Ishiuchi and Takeichi, 2011, Motegi et al., 2011, Suzuki et al., 2004).

Our evidence indicates that engineered lower-affinity interactions between the Par3/Baz CR3 domain and the aPKC kinase domain result in Par3/Baz CR3 phosphorylation (Figures 5A–5C). Mutation of either the F-X-R or K-H-T site that our structure shows are important for a high-affinity inhibitory interaction leads to a relocalization of Par3/Baz away from the apical domain (where the Par complex resides) to AJs, similar to a phospho-mimetic S980E mutant in Par3/Baz (Figures 6A–6D). Combining both mutations further lowers the affinity, leading to a form of Par3/Baz unable to engage aPKC that cannot be phosphorylated by it. Such a mutant Par3/Baz also relocalizes to AJs. Thus, Par3/Baz that fails to form a stable inhibitory Par complex will localize to AJs either through aPKC-mediated phosphorylation or through a loss of interaction.

Why does phosphorylation of Par3/Baz cause its localization to AJs? Our findings show that phospho-Par3/Baz dramatically reduces its affinity for aPKC and thus the phosphorylation event precludes it from joining the Par complex. Phosphomimic S980E Par3/Baz is known to localize to AJs, just like the affinity-lowering A-X-A or A-A-T mutants of Par3/Baz (Figure 6). Furthermore, the behavior of phospho-resistant mutant S980A Par3/Baz (which only localizes with aPKC) can be reversed in A-X-A S980A or A-A-T S980A double mutant Par3/Baz (which only localizes to AJs) (Figure 6). Previous studies have proposed that the AJ localization of Par3/Baz results from exclusion from the apical domain upon aPKC phosphorylation combined with exclusion from the basolateral domain upon Par1 phosphorylation (Tepass, 2012). Taken together, our data stimulate a model in which aPKC-driven phosphorylation of Par3/Baz can be recapitulated simply by weakening the Par3/Baz interaction affinity by manipulating the sequences flanking the consensus PKC phosphorylation site (Figure 7).

Our findings implicate both Par3/Baz and Kibra as aPKC inhibitors that are also known substrates. An analogous situation arises for LGL, a known inhibitor of aPKC that is also phosphorylated at multiple serine sites (Bailey and Prehoda, 2015). There are also precedents for protein kinase dual-action inhibitor/substrates. The protein kinase A (PKA) regulatory subunit RIIβ has an RRXS motif that is phosphorylated by PKA, leading to its stable association with and inhibition of PKA (Zhang et al., 2015). In this context the modification functions as part of a single-turnover phosphoryl transfer reaction. For Par3 and other F-X-R-containing proteins, a different role is likely whereby phosphorylation by aPKC provokes Par3/Baz dissociation, as shown in vitro using CR3 peptides and in vivo using phospho-mimetics. Another example is the cyclin-dependent kinase inhibitors p21/p27/KIP, which are able to both inhibit cyclin-dependent kinases as well as being efficient substrates (Russo et al., 1996).

Our findings suggest that aPKC is inhibited by Par3/Baz within the Par complex, yet it is known that the Par complex contains active aPKC kinase and can phosphorylate many substrates (such as Lgl and Par1 in *Drosophila* epithelia and *Caenorhabditis elegans* zygotes, and Miranda in *Drosophila* neuroblasts). One possible explanation for this open issue is that discrete functional states of the Par complex (Par6-aPKC-Cdc42-Par3) may exist. Par3-dependent recruitment of aPKC to apical membranes may evoke a higher-order oligomer consistent with known Par3 CR1-dependent oligomers (Benton and St Johnston, 2003a). Conversely, phosphorylation of T833^Par3^ by ROCK kinase or a lack of T412^PKCι^ phosphorylation by PDK1 would generate functionally distinct forms of the Par complex, unable to be inhibited by Par3 CR3 as discussed earlier. Equally, association of partner proteins close to the aPKC αC helix could also block the formation of an R+5 pocket and prevent CR3-mediated inhibition. Therefore, further experiments are required to characterize precisely which polarity signal(s) provoke Par3 phosphorylation by overcoming CR3 antagonism.

In conclusion, our findings provide a molecular basis for Par3-mediated antagonism of aPKC that affects apical versus junctional polarization of Par3/Baz in epithelia.

## Experimental Procedures

### Protein Construct Design, Expression, and Purification

Mammalian plasmids pEGFP-PKCι-WT, pEGFP-PKCι-DD/AA (D339A/D382A), pK-myc-Par3-WT (Addgene, plasmid 19388), pK-myc-Par3-AXA (F818A/R820A), and pK-myc-Par3-A (S827A) included human PKCι and Par3 cDNAs. Mutagenesis of PKCι and Par3 was performed using QuikChange (Stratagene). Recombinant human PKC-iota kinase domain (PKCι_KD_) was prepared using a baculovirus encoding residues 248–596 (GenBank: NM_002740.5), fused to a glutathione S-transferase (GST) tag as described previously (Kjaer et al., 2013). See Supplemental Experimental Procedures for a detailed description. In brief, the protein was expressed in Hi5 cells by co-infection with the above virus and a PDK-1 virus using standard protocols (Oxford Expression Technology). The GST tag was used for affinity purification and removed by 3C protease cleavage using standard protocols. Two distinct phospho-species PKCι_KD_-2P and PKCι_KD_-1P were separated by ion-exchange chromatography (Hi-Trap Q column, GE Healthcare).

### Enzymatic Assay and Fluorescence Anisotropy Binding Assay

The ADP Quest kit (DiscoveRx) was used to determine the k_cat app_ and K_M app_ values for ATP against the PKCι_KD_-1P and PKCι_KD_-2P using a series of synthetic peptide substrates as described by Kjaer et al. (2013). The reactions were measured every 2 min for 30 min in a 384-well plate using a Safire^2^ plate reader (Tecan). The kinetic constants were determined by fitting the data to the Michaelis-Menten equation. Data are represented as mean ± SEM. Fluorescence anisotropy assays were performed to determine the K_D_ for each peptide labeled with a fluorescein tag following standard protocols using a Safire^2^ plate reader (Tecan). The anisotropy values were normalized and the K_D_ was determined using non-linear regression. All experiments were performed in triplicate and for at least three independent protein preparations.

### Structure Determination of Nucleotide-Bound PKCι_KD_-2P Complexes

PKCι_KD_-2P was incubated with a 3-molar excess of nucleotide or analog with either Mg^2+^ or Mn^2+^ (see Table 1) and a 3-molar excess of peptide. Crystallization was performed using the hanging-drops method with 1:1 ratio of protein to precipitant at 20°C. X-ray data were collected at synchrotrons as specified by Table 1 and data were processed using either XDS (Kabsch, 2010b) and Xscale (Kabsch, 2010a) or D^∗^Trek (Pflugrath, 1999) and Scala/Pointless (Collaborative Computational Project-Number 4, 1994). Structures were determined by molecular replacement performed using Phaser (McCoy et al., 2007) using a previous PKC ι-2P structure as a search model (PDB: 3A8W). Refinement was carried out in Phenix (Adams et al., 2010) with cycles of model building in Coot (Emsley and Cowtan, 2004).

### Cell Culture and Transfection

HCT-116 cells grown in McCoy's 5A medium containing 10% bovine fetal calf serum and penicillin/streptomycin (Invitrogen) were transfected (10-μg portion of DNA or 5-μg + 5-μg portions of DNA for co-transfections) using Fugene HD (Roche) according to the manufacturer's instructions. The cells were then grown in normal medium for 36 hr.

### Antibodies, Immunoprecipitation, and Immunoblotting

The following antibodies were used for immunoblotting: mouse monoclonal anti-PKCι (recognition for human PKCι), mouse monoclonal anti-Myc (9 × 10^10^), rabbit polyclonal anti-Par3 (Millipore), and rabbit polyclonal anti-GFP antibody (Santa Cruz Biotechnology). Anti-phospho-S827 Par3 antibody was raised in-house using an antigen lacking the F-X-R site of Par3_CR3_. Immunoprecipitation and immunoblotting was carried out as described in Supplemental Experimental Procedures.

### Dimethyl Labeling and Quantification of Bazooka Wild-Type and Mutant S980 Phosphorylation in S2 Cell Extracts

After SDS-PAGE, in-gel stable isotope dimethyl labeling was performed according to published protocols. The heavy reaction was performed using ^13^CD_2_O formaldehyde creating a mass difference of 6 Da per primary amine group between heavy and light dimethylated peptides. After extensive washing of gel pieces, the in-gel dimethylated proteins were then subjected to overnight in-gel trypsin digestion at 37°C. The following day peptides were extracted and subjected to another round of reductive dimethylation reactions aimed at methylating peptide N termini. Peptide mixtures were acidified and prepared for liquid chromatography-mass spectrometry analysis using an Ulimate3000 high-performance liquid chromatograph coupled to a Q-Exactive mass spectrometer (Thermo Fisher). A targeted scan was performed for the S980-containing peptides and this was alternated with a top-10 data-dependent acquisition scan. Mascot-generated DAT files were converted to Skyline-compatible biblio.spec libraries, and heavy and light peak areas were extracted by Skyline software version 2.5.0.6079 (MacLean et al., 2010).

### *Drosophila* Genetics and Oligonucleotides

Expression of *UAS*-driven transgenes in follicle cells was achieved with the GR1.Gal4 line. *UAS.GFP-Baz* lines were constructed by mutagenizing the full-length Baz cDNA in pDONR, followed by transfer to the pPGW (pUASP-EGFP-Gateway) vector for transgenesis (Bestgene). The *UAS.GFP-BazS980E* line was a gift from F. Pichaud. Primers used for mutagenesis are described in Supplemental Experimental Procedures.

### *Drosophila* Antibodies and Immunohistochemistry

Ovaries were dissected in PBS, fixed for 20 min in 4% paraformaldehyde in PBS, washed for 30 min in PBS/0.1% Triton X-100 (PBST), and blocked for 15 min in 5% normal goat serum/PBST (PBST/NGS). Primary antibodies were diluted in PBST/NGS and samples were incubated overnight at 4°C. Either optical cross-sections through the middle of egg chambers or apical sections of the follicular epithelium are shown. Primary antibodies used are described in Supplemental Experimental Procedures.

## Author Contributions

E.V.S. performed all stages of the Mg-AMPPNP-Par3_CR3_-PKCι_KD_-2P peptide structure determination and refinement. A.P. refined the Mn-ADP-PKCι_KD_-FXR_short_ peptide structure. M.I. purified, crystallized, and refined the AMPPCP-complexed PKCι_KD_-2P structure. E.V.S. and M.I. carried out kinetic assays and fluorescence anisotropy measurements. P.K. and S.K. assisted with protein production and virus preparation. A.E. prepared S2 cell extracts containing wild-type and mutant Baz protein. K.B. and B.S. performed the dimethyl-labeling and mass spectrometry analyses. B.K. prepared and characterized recombinant PKCι_KD_ and PKCζ_KD_ proteins. P.S. prepared constructs for full-length human Par3 and mutants. N.O’R. purified all peptides used in this study. P.R. conducted co-immunoprecipitations using full-length Par3 with full-length PKCι and raised the anti-phospho-Par3 antibody. M.L. prepared the PKCι D/D mutant. G.F. and B.J.T. carried out all the *Drosophila* experiments. M.I. prepared the figures. N.Q.M. and B.J.T. planned the project, designed experiments, and wrote the paper.

## Figures and Tables

**Table 1:** Data Collection and Refinement Statistics

	PKCι_KD_-2P/Par3 CR3 Peptide/Mg-AMPPNP	PKCι_KD_-2P/AMPPCP	PKCι_KD_-2P/Mn-ADP/AlF_3_/FXR-Short Peptide
**Data Collection**			

Space group	*P*3_1_21	*P*2_1_2_1_2_1_	*P*2_1_2_1_2_1_
Cell Dimensions			
*a*, *b*, *c* (Å)	82.0, 82.0, 90.8	61.1, 65.1, 87.4	79.0, 84.2, 111.8
α, β, γ (°)	90, 90, 120	90, 90, 90	90, 90, 90
Resolution (Å)	45.45–1.95 (2.06–1.95)	52.23–1.79 (1.84–1.79)	67.28–3.25 (3.43–3.25)
Completeness (%)	99.6 (997.5)	100 (100)	99.6 (99.9)
Multiplicity	8.2 (7.0)	9.5 (9.6)	3.8 (3.6)
*R*_meas_ (%)_pim_	9.0 (64.0)	17.3 (200)	20.0 (55.1)
*R*_p.i.m._ (%)_pim_	3.1 (23.3)	5.6 (63.4)	9.9 (27.5)
<*I*>/<σ*I*>	15.0 (3.0)	8.2 (1.4)	6.3 (2.5)
Total no. of observations	216,286 (25,839)	323,684 (23,802)	46,484 (6,806)
Total no. unique	26,242 (3,689)	33,998 (2,474)	1,200 (1,753)

**Structure Refinement**			

*Z*_a_	1	1	2
Reflections	25,607	33,825	12,145
*R*_work_ (%)	15.0	18.8	25.66
*R*_free_ (%)	21.7	23.1	28.36
No. of protein atoms	A = 2,719	A = 2,701	A = 2,527, B = 2,489, F = 100, G = 76
No. of ligand atoms	B = 154, D = 66	B = 48	C = 27, D = 27, other = 27
No. of solvent atoms	C = 2, E = 170, F = 14	C = 21, D = 244, E = 5, F = 8, G = 18, I = 8	E = 25
Mean *B* Factor			
Protein	A = 26.1	A = 23.7	(A, B, F, G) = 44.00
Ligand	B = 35.0, D = 29.5	B = 28.0	all (non-water) = 41.7
Solvent	C = 45.6, E = 38.1, F = 45.5	C = 61.3, D = 35.7, E = 65.3, F = 58.4, G = 46.1, I = 52.9	E = 32.0
RMSD bonds (Å), angles (°)	0.008, 1.100	0.004, 0.765	0.003, 0.733
Ramachandran Plot (%)			
Favored	98.0	97.6	94.5
Allowed	2.0	2.1	5.4
Outliers	0.0	0.3	0.15
	where A = protein B = peptide C = K^+^ ion D = AMPPNP E = water F = glycerol	where A = protein B = AMPPCP C = formate D = water E = imidazole F = MPD G = PEG I = acetate	where A, B = protein F, G = peptide C, D = ADP E = water

RMSD, root-mean-square deviation; PEG, polyethylene glycol; MPD, 2-methyl-2,4-pentanediol.

## Accession Numbers

Coordinates and structure factors for PKCι_KD_-2P-Par3_CR3_, PKCι_KD_-2P-FXR_short_ peptide, and nucleotide-bound PKCι_KD_-2P have been deposited in the PDB with the accession numbers PDB: 5LI1, 5LIH, and 5LI9, respectively.

## Supplemental Information

Document S1. Supplemental Experimental Procedures and Figures S1–S7

Document S2. Article plus Supplemental Information
