# Supplementary material for: aPKC Inhibition by Par3 CR3 Flanking Regions Controls Substrate Access and Underpins Apical-Junctional Polarization
Source: Dev Cell. 2016 Aug 22;38(4):384–98. doi: 10.1016/j.devcel.2016.07.018 (PMC4998004; doi:10.1016/j.devcel.2016.07.018)
Supplement: Document S1. Supplemental Experimental Procedures and Figures S1–S7 [file mmc1.pdf]

Developmental Cell, Volume *38*

Supplemental Information

aPKC Inhibition by Par3 CR3 Flanking Regions Controls Substrate Access and Underpins Apical-Junctional Polarization

Erika V. Soriano, Marina E. Ivanova, Georgina Fletcher, Philippe Riou, Philip P. Knowles, Karin Barnouin, Andrew Purkiss, Brenda Kostelecky, Peter Saiu, Mark Linch, Ahmed Elbediwy, Svend Kjær, Nicola O'Reilly, Ambrosius P. Snijders, Peter J. Parker, Barry J. Thompson, and Neil Q. McDonald

Figure S1 related to Figure 1

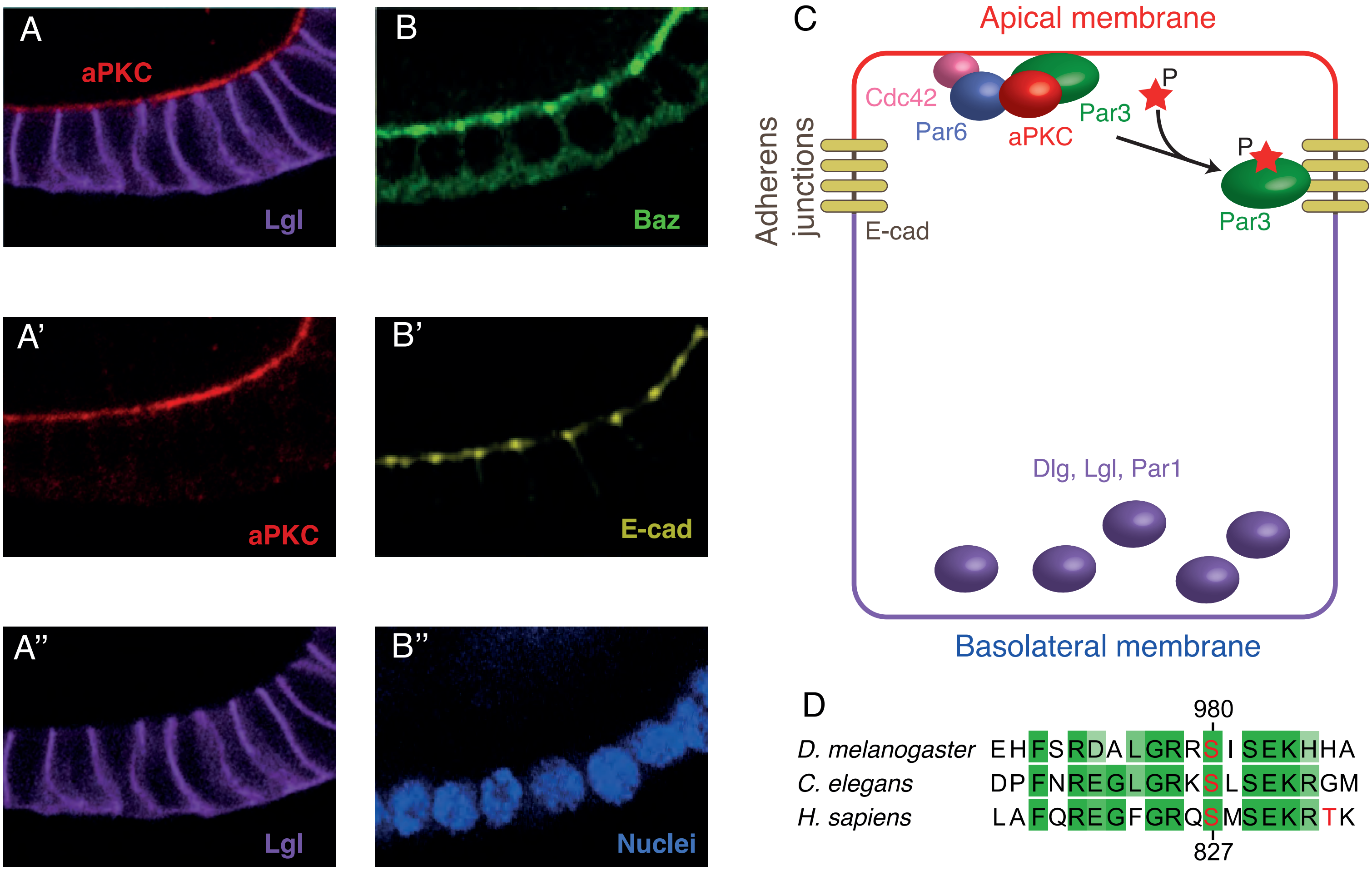

Apical and junctional localization of Par3/Baz in Drosophila follicular epithelium.
(A) and **(B)** Imaging of Drosophila follicular epithelial cells showing the localisation of various polarity determining proteins to distinct apical, junctional or basolateral locations. White arrows indicate the adherens junctions, staining positive for Baz. Grey arrows indicate the pool of apical membrane localised Baz **(C)** Model for Par3/Baz junctional polarization proposed by Morais de Sa and colleagues relating pools of Baz found at each location depending on their aPKC-site phosphorylation status (Morais-de-Sa et al., 2010). **(D)** Sequence alignment of Par3/Baz CR3 regions from three species highlighting the conserved human S827 phosphorylation site.

Figure S2 related to Figure 1

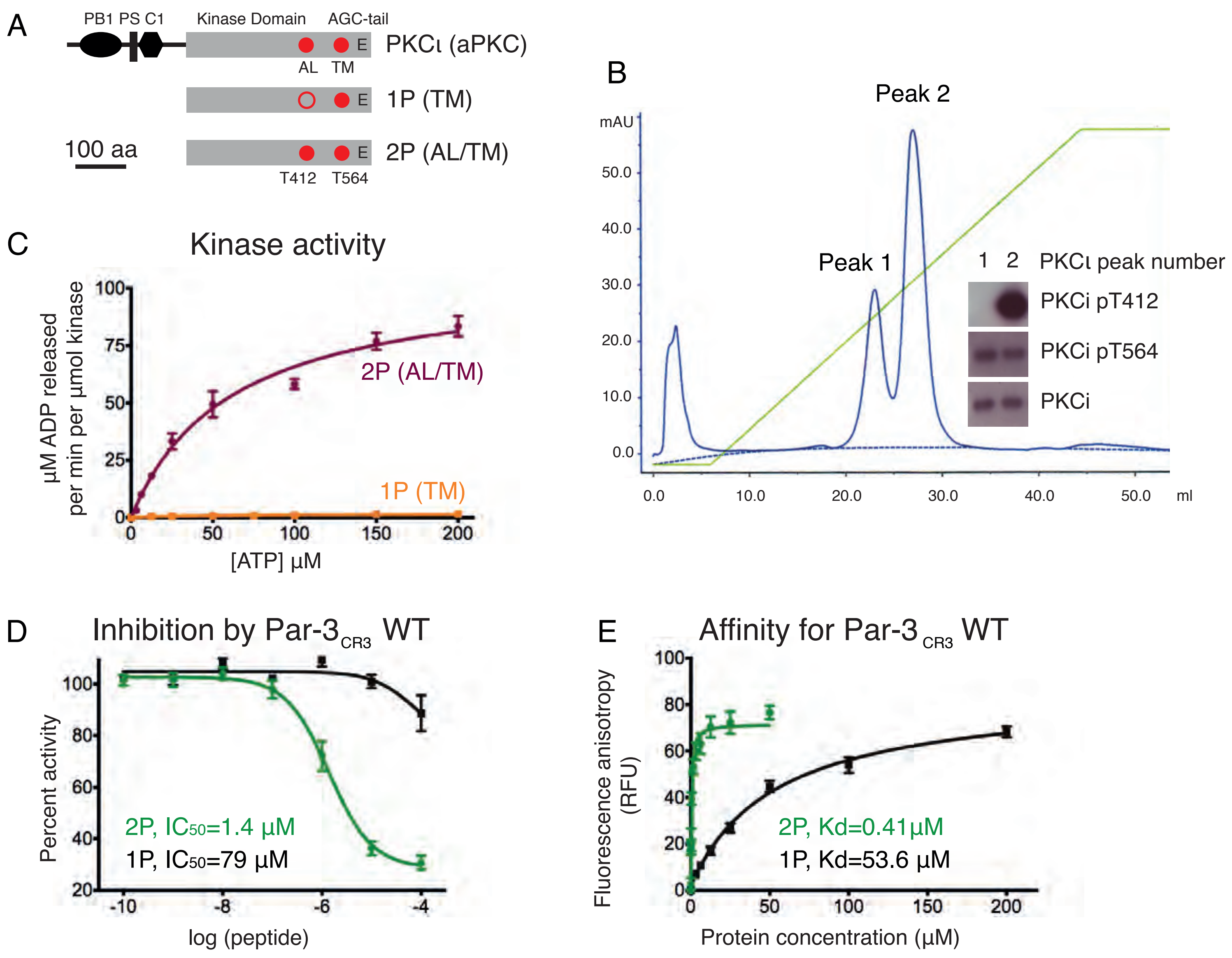

Purification, characterisation and inhibition of primed (2P) and partially primed (1P) recombinant forms of PKCιKD.

(A) Domain structure of PKCι and the location of the priming phosphorylation sites in the kinase domain. AL (activation loop) contains the PDK1 phospho-site T412 and TM (turn motif) contains the mTOR phosphorylation site T564. **(B)** Purification of recombinant human PKCι kinase domain (PKCιKD) by ion exchange chromatography indicates two discrete species of PKCιKD ; a primed PKCιKD-2P and a partially primed PKCιKD-1P form. Western blot shows the phosphorylation state of PKCιKD in each peak. **(C)** In vitro kinase assay comparing the catalytic activity of the primed PKCιKD-2P and a partially primed PKCι-1P form. **(D)** In vitro kinase assay IC50 curves for Par3CR3 reveals strong inhibition of the primed PKCιKD-2P and very weak inhibition of partially primed PKCιKD-1P forms **(E)** High affinity of fluorescein-labelled Par3CR3 for the primed PKCιKD-2P but low affinity for partially primed PKCιKD-1P as measured by fluorescence anisotropy. Potent PKCιKD-2P inhibition correlates with a high affinity PKCιKD-2P binding.

Figure S3 related to Figure 2 and 3

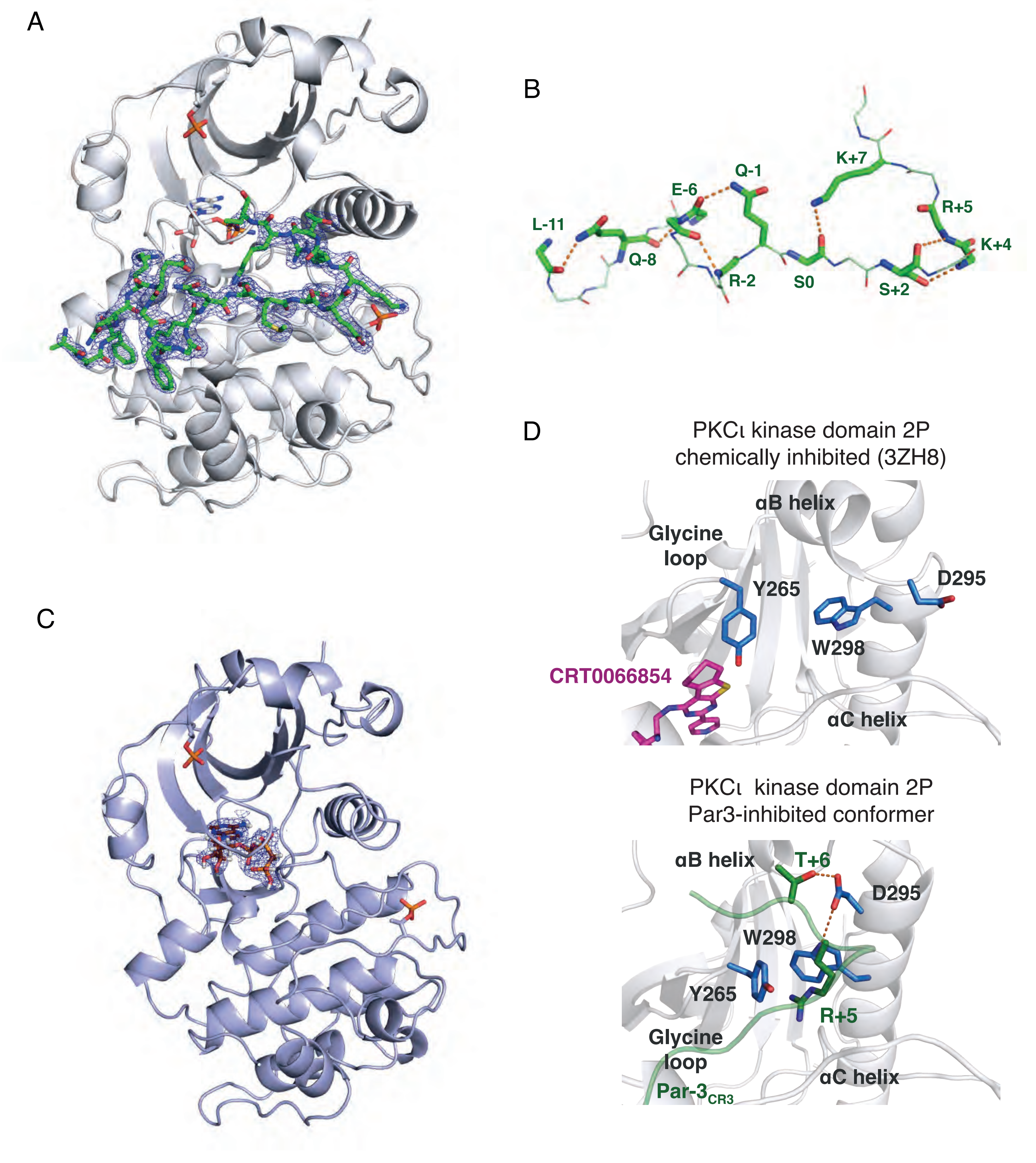

Electron density for Par3CR3 bound to PKCιKD, its internal hydrogen-bonds and electron density for ATP nucleotide bound to PKCιKD.

(A) Electron density for Par3CR3 peptide superposed onto the structure of Par3CR3 bound to PKCιKD-2P shown with the SIGMAA-weighted 2Fo-Fc electron density omit map (sigma = 1.0). **(B)** Internal hydrogen bonds formed in the Par3CR3 peptide. **(C)** Electron density for AMPPCP superposed onto the structure of AMPPCP bound to PKCιKD-2P shown with the SIGMAA-weighted 2Fo-Fc electron density omit map (sigma = 1.0). **(D)** Close-up of R+5 pocket in the previously solved inhibitor-bound PKCιKD-2P structure (3ZH8) compared to an equivalent view of the Par3 CR3-inhibited structure.

Figure S4 related to Figure 4

A

PKCε PS	ERMRPRKRQGAVRRRV
PKCε PSS	ERMRPRKRQGSVRRRV
FXR short	FKRQGSVRRRV
Nishikawa *et al.*	FXRXXSψψ
PKCα PS	DVANRFARKGALRQKN
PKCι PS	GEDKSIYRRGALRRWR

B

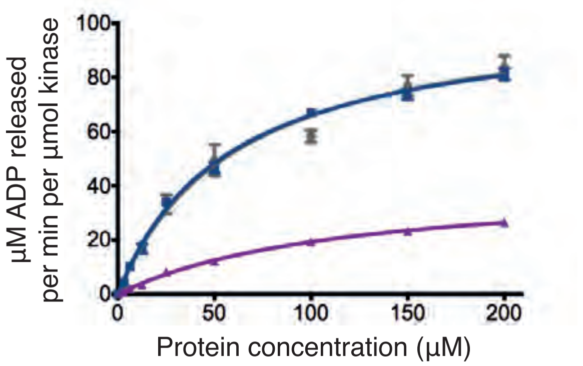

C

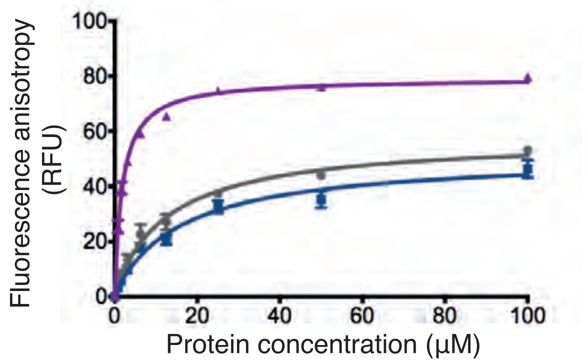

D

	Sequence	k_{cat}^{app} ($^{-1}$ min)	k_{m}^{app} (μM)	k_d (μM)
ε_{pss}	ERMRPRKRQGSVRRRVHQVN	106.1±6	61.2±9	13.3±2.1
ε_{pss} -5F*	ERMRPFKRQGSVRRRVHQVN	42±14	118.2±14	1.8±0.1
ε_{pss} +5F	ERMRPRKRQGSVRRRFHQVN	103.7±2.6	57.1±3.9	16.4±3.5

E

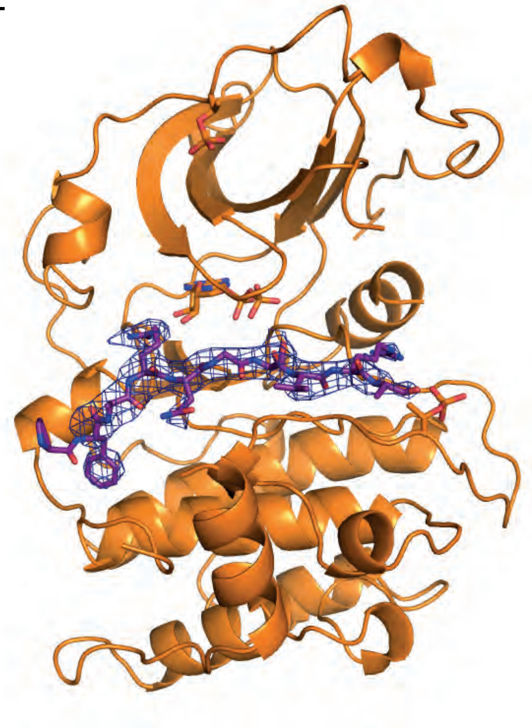

F

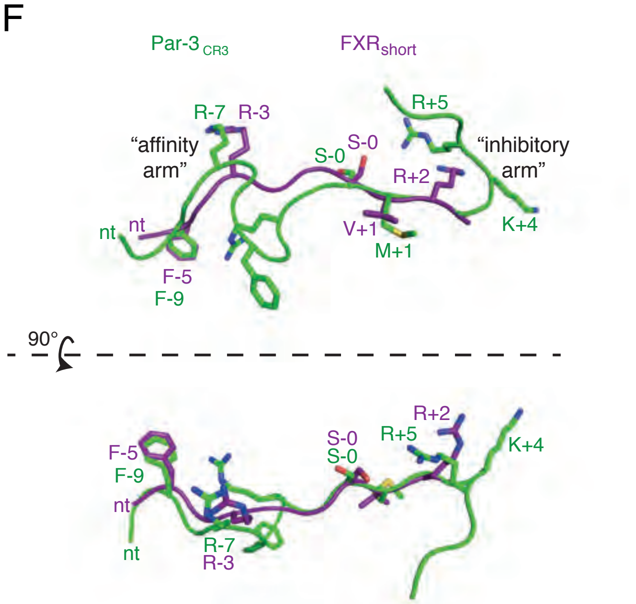

Characterisation and crystallisation of an FXR-short peptide derived from the PKCε pseudo-substrate motif and resembling an aPKC substrate consensus defined by Nishikawa et al.

(A) Alignment of selected PKC pseudo-substrate sequences with the PKCε pseudo-substrate (PS) and an alanine to serine mutation (PSS), the FXRshort peptide and the consensus aPKC motif first identified by Nishikawa et al. (Nishikawa et al., 1997). The serine phosphorylation site mutation introduced is shown in red and the equivalent alanine residue in pseudo-substrate motifs of PKC isozymes is orange. **(B)** In vitro kinase assay comparing the catalytic activity of PKCιKD-2P towards wild type and mutant PKCεPSS peptides. **(C)** Affinity of fluorescein-labelled εPSS wild type and mutant peptides for PKCιKD-2P measured by fluorescence anisotropy. **(D)** Summary table of kcat, Kd and Km constants between wild type or various εPSS mutants and PKCιKD-2P. Introducing a F-5 residue but not a F+5 residue enhances peptide affinity for PKCιKD-2P and but reduces substrate turnover. This allowed capture of the FXRshort peptide bound to PKC ιKD-2P **(E)** Electron density for FXRshort peptide superposed onto the FXRshort peptide-PKCιKD-2P structure shown with the SIGMAA-weighted 2Fo-Fc electron density omit map (sigma = 1.0). Mn ions and AlF_3 were omitted for clarity. **(F)** Orthogonal views of a structural superposition of the Par3CR3/PKCιKD-2P complex with the FXRshort /PKCιKD-2P omitting the aPKC kinase domain for clarity. The FXR motif (F-9/X/R-7)from Par3CR3 superposes precisely onto a structurally equivalent FXR motif (F-5/X/R-3) from the FXRshort peptide.

Figure S5 related to Figure 5

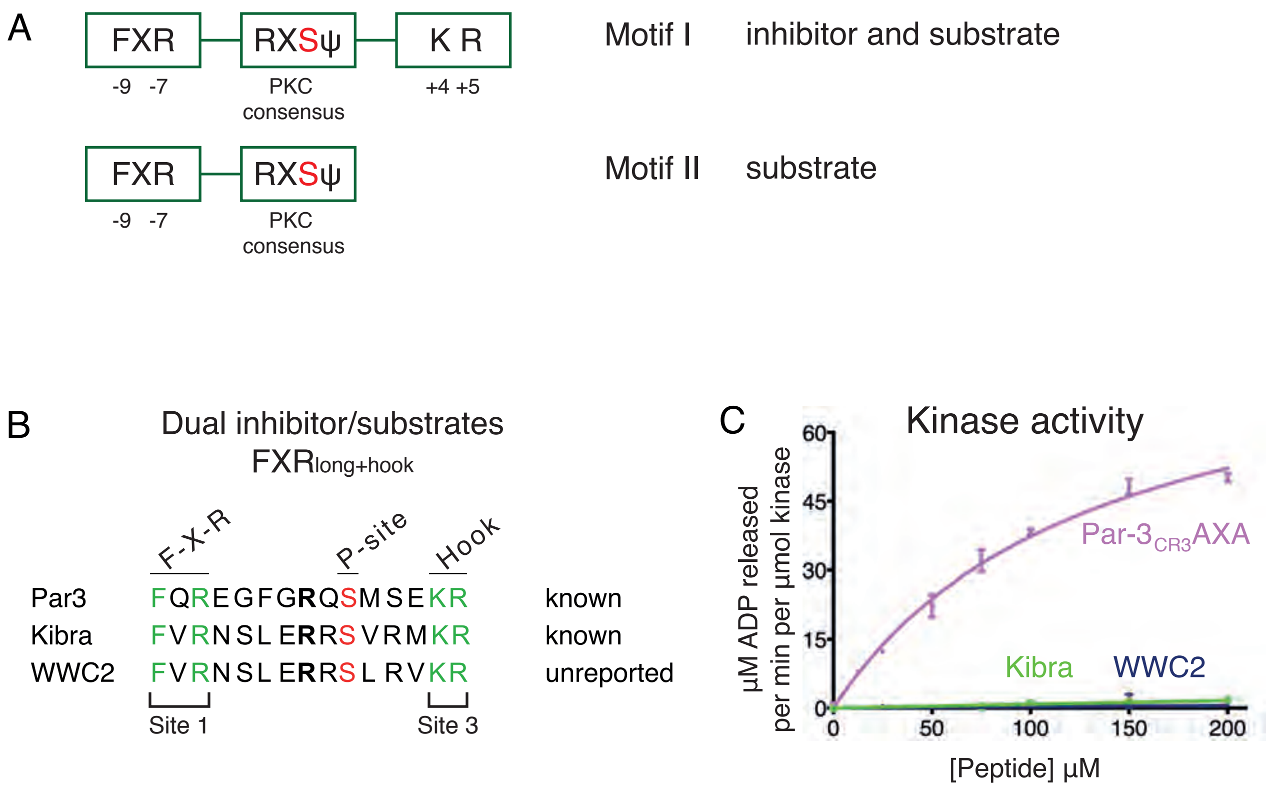

Presence of K-R inhibitory motif within an aPKC substrate defines a dual inhibitor/substrate function in vitro.

(A) Schematic representation of two groups of aPKC substrates distinguished by the presence or absence of the inhibitory arm. **(B)** Sequence alignment of human Par3, Kibra and WWC2. **(C)** In vitro kinase assay showing that both Kibra and WWC2 inhibit PKCιKD-2P. A Par3CR3 with an A-X-A mutation was used as a positive control (pink) and a Par3CR3 peptide as a negative control (not shown).

Figure S6 related to Figure 5

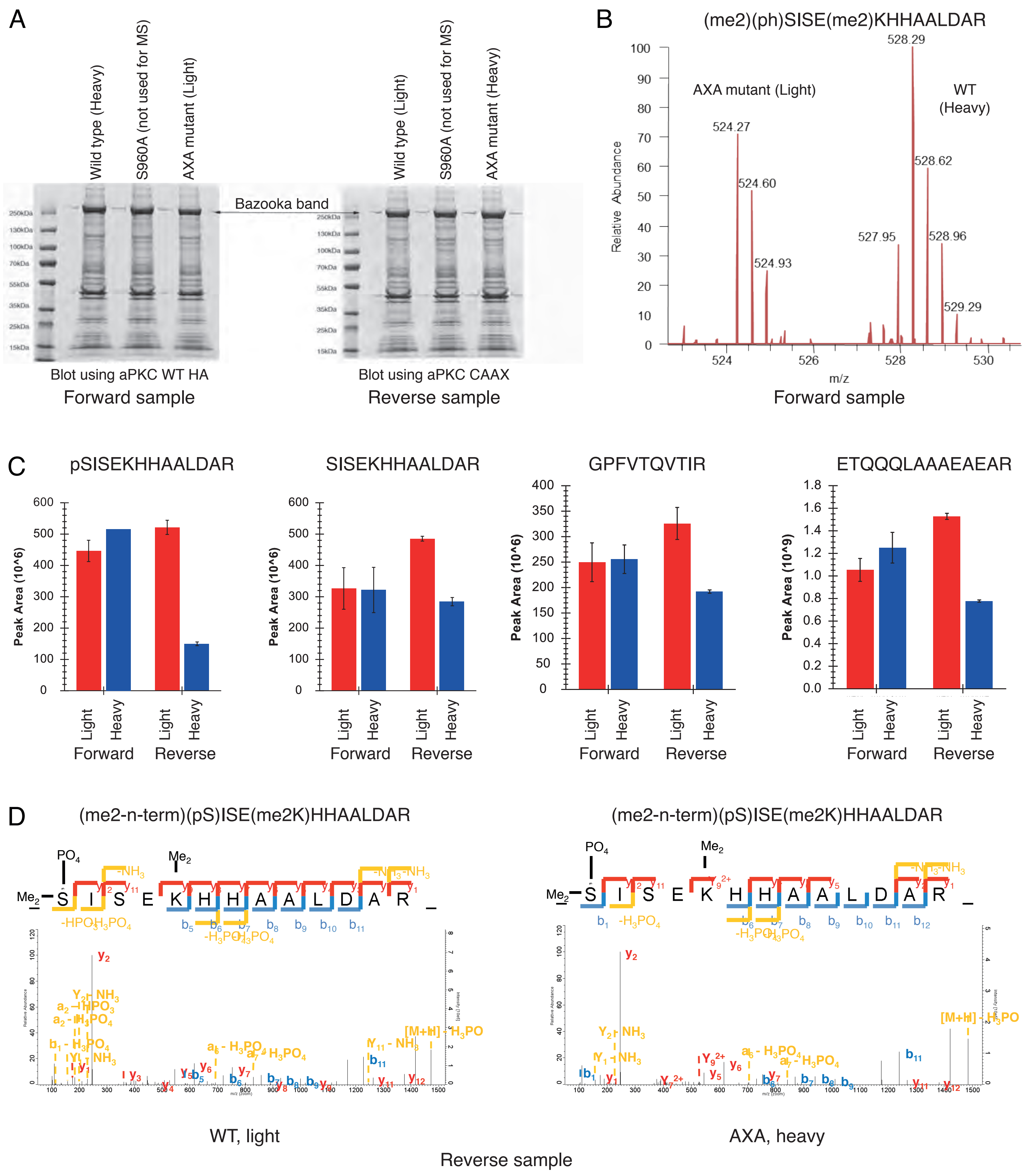

Wild type Bazooka and A-X-A mutant are phosphorylated at S980 in cell extracts
(A) Purification and labelling scheme of GFP-tagged Bazooka. Both samples were dimethylated but in the forward sample wild type Bazooka was heavy labelled whereas in the reverse sample the A-X-A mutant was heavy labelled. **(B)** Mass spectrum of forward sample displaying the heavy and light peaks, separated by four m/z units. **(C)** Skyline calculated peak areas for light (red) and heavy (blue) Bazooka peptides. Error bars indicate standard errors over triplicate LC-MS injections. Two non-phosphorylated peptides were used as controls **(D)** Annotation of MS/MS spectra for light and heavy peptides using MaxQuant viewer, significant identifications obtained with both MaxQuant/Andromeda and Mascot (data not shown).

Figure S7 related to Figure 6

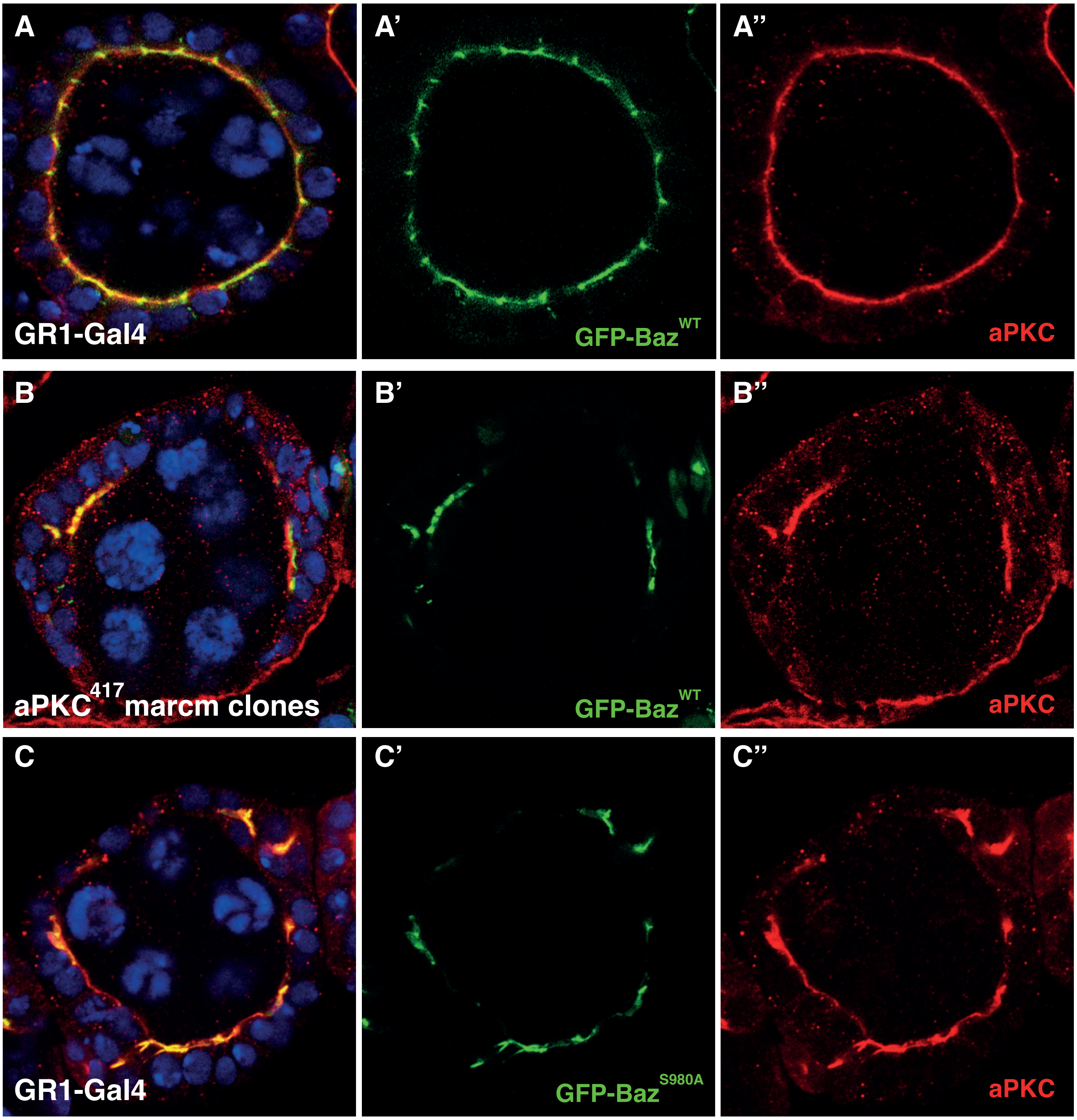

Bazooka co-localises with aPKC in the absence of kinase activity.
(A) Drosophila egg chambers expressing wild-type Bazooka-GFP in wild-type follicle cells. Note the segregation of aPKC and Baz-GFP, with Baz-GFP also localising to adherens junctions. **(B)** Expression of Bazooka-GFP in kinase dead aPKC mutant cells (aPKC417) leads to either co-localisation with aPKC or complete loss of the apical domain. **(C)** Expression of S980A Bazooka-GFP in wild-type cells leads to co-localisation with aPKC at the apical domain.

Supplemental Experimental Procedures

Protein construct design, expression, and purification

The cDNA encoding human PKC iota kinase domain (PKCι$_{KD}$) comprising residues 248-596 (GenBank accession number NM_002740.5) was amplified and inserted into a pBacPAK-His3 vector (Clonetech) using BamHI and XhoI restriction sites. The vector was modified to have a glutathione S-transferase (GST) followed by a 3C protease cleavage site upstream of the kinase domain. A C586S mutation was also introduced in the kinase domain using QuikChange (Strategene) using standard protocols.

Viruses used for infection were obtained under standard protocol (Oxford Expression Technology UK). PKCι virus was used to infect Hi5 or SF21 cells with a multiplicity of infection of 2 with cells were grown in shaker flasks in SFIII media (Life Technologies) and 10 μg/ml gentamycin (Life Technologies). Cells were harvested 72 hours post infection and resuspended in lysis buffer (20 mM HEPES pH 7.4 (Sigma), 150 mM NaCl (Sigma), 10 mM Benzamidine (Sigma), 0.2 mM AEBSF (Melford Laboratories), 1 mM EDTA (Sigma), and 1 mM DTT (Melford Laboratories)). All purification steps were done at 4 °C or on ice. Cells were lysed by sonication and spun down at 30,000*g* for 30 minutes. PKCι$_{KD}$ was purified using glutathione sepharose 4B beads (Amersham Biosciences), followed by removal of the GST affinity tag with GST-3C protease (PreScission Protease, Amersham Bioscience) and ion-exchange chromatography (Hi-Trap Q column, GE Healthcare). The major peak from ion-exchange is not phosphorylated at the activation loop T412, but is phosphorylated at the turn motif site T564 (PKCι$_{KD}$-1P) and used as a control in the kinetic and binding assays. To produce the primed kinase domain (PKCι$_{KD}$-2P) phosphorylated at both T412 and T564, Hi5 cells were co-infected with PDK-1 virus at a multiplicity of infection of 1. All other expression and purification procedures remain the same. Both proteins were dialyzed into the final buffer (20 mM Tris pH 7.5, 50 mM NaCl, 5% glycerol, 1 mM DTT) and concentrated before use.

In vitro kinetic assays for PKCι$_{KD}$-2P

The ADP Quest kit (DiscoveRx) was used to determine the $k_{cat\ app}$ and $K_{m\ app}$ values for ATP against the PKCι_{KD}-1P and PKCι_{KD}-2P. The assay uses a coupled reaction to convert ADP to a product that has a fluorescence excitation at 530 nm and emission at 590 nm. Several synthetic peptide substrates were used including a model peptide (ERMRPRQGSVRRRV) derived from a PKCε pseudo-substrate sequence with an alanine-serine change. We found this peptide to be an efficient substrate peptide for aPKC isozymes. Peptides from human Par3$_{CR3}$ (residues 816-835) and F-X-R$_{short}$ peptide (ERMFPRQGSVRRRV) related to the Nishikawa and co-workers consensus were also used in the study (Nishikawa et al., 1997). For the $k_{cat\ app}$ and $K_{m\ app}$ reactions, the ATP concentration ranged from 0-200 μM, while the synthetic peptide substrates were kept constant at 200 μM to avoid product inhibition. The reactions were measured every two minutes for 30 minutes in a 384-well plate using a Safire^2 plate reader (Tecan). The kinetic constants were determined by fitting the data to Michaelis–Menten equation using the graphics program Prism (GraphPad Prism, version 5.0d, GraphPad Software). To determine the IC_{50} of various Par3$_{CR3}$ peptides, the Par3$_{CR3}$ peptide concentration was varied from 10 nM to 0.1 mM, with the model peptide and ATP concentration kept at a constant 20 μM each. The reactions were then scanned after 30 minutes and the IC_{50} was determined using a non-linear fit in the graphics program Prism.

Fluorescence anisotropy binding assays

Fluorescence anisotropy assays were performed to determine the K_d for each peptide following a previously described protocol (Guettler et al., 2008). Binding assays were performed in 20 mM Tris pH 7.5, 50 mM NaCl, 5% glycerol and 0.5 mM TCEP. The reaction mixtures contained fluorescein labelled peptides at a constant 50 nM concentration, while the protein concentration ranged from 0-200 μM. The 20 μl reactions were carried out in 384-well plate and measured after 30 minutes on the Safire^2 plate reader (Tecan) with the excitation at 470 nm and emission at 525 nm. The anisotropy values were normalized and the K_d was determined using a nonlinear regression with the graphics program Prism and an equation previously described (Heyduk and Lee, 1990).

Structure determination of nucleotide-bound PKCι_{KD}-2P with Par3$_{CR3}$

$PKC\iota_{KD}$-2P was incubated with a 3 molar excess of AMPPNP, magnesium sulphate and a 3 molar excess of human Par3 peptide (residues 816-841) for an hour on ice. Crystallization was performed using hanging drops method with 1:1 ratio of protein to precipitant at 20 °C. Crystals grew in 2-3 weeks using 32% Peg 2000 MME and 0.08 M KSCN and were cryo-cooled using 20% glycerol. These crystals belonged to $P3_121$ space group. Data were collected at ESRF ID-29. Data were processed in XDS (Kabsch, 2010b) Xscale (Kabsch, 2010a), with molecular replacement performed using Phaser (McCoy et al., 2007) using a previous PKCι-2P structure as a search model (PDB code: 3A8W) with the activation loop and other flexible loops removed from the search model. These trigonal crystals diffracted to Bragg spacings of 2.0 Å with one molecule in the asymmetric unit. Initial rigid body refinement in Phenix (Adams et al., 2010) was followed by model building in COOT (Emsley and Cowtan, 2004). Further refinement was carried out in Phenix before the AMP-PNP molecule was modelled into the density. No density was observed for the γ-phosphate of AMPPNP consistent with the AMPPNP being hydrolysed to AMPPN and inorganic phosphate under these crystallisation conditions. $Par3_{CR3}$ peptide residues 816 to 835 were well ordered with good density, whereas residues 836-841 could not be modelled, presumed to be disordered. Two Mg ions were evident in the structure, one bridged by the α and β-phosphates of AMPPN and a second site bridged $T833^{Par3}$ and $D295^{PKC\iota}$ (Figure 2G).

Structure determination of nucleotide-bound $PKC\iota_{KD}$-2P

$PKC\iota_{KD}$-2P was incubated with a 3 molar excess of AMPPCP for an hour on ice. Crystallization was performed using hanging drops method with 1:1 ratio of protein to precipitant at 20 °C. Crystals grew in 2-3 days using 25% Morpheus precipitant mix 4 (Molecular dimensions), 10% Carboxylic acids (Molecular dimensions), Buffer system 1 pH=6.3 (Molecular dimensions) and were cryo-cooled without additional cryoprotectant. These crystals belonged to $P2_12_12_1$ space group. Data were collected at Diamond I24 and were processed as above. Molecular replacement was performed using Phaser (McCoy et al., 2007) using a previous PKCι-2P structure as a search model (PDB code: 3A8W) with the activation loop and other flexible loops removed from the search model. These crystals diffracted to Bragg spacings of 1.8 Å with one molecule in the asymmetric unit. The data was solved and refined using Phaser and

Phenix as described above. Further refinement was carried out in Phenix before the AMPPCP molecule was modelled into the density.

Structure determination of nucleotide-bound PKCι$_{KD}$-2Pwith a FXR$_{short}$ substrate peptide

PKCι$_{KD}$-2P was incubated with a 3 molar excess of ADP, manganese sulphate, 12 molar excess of AlF_3 (a known transition-state analogue for phospho-transfer) and a 3 molar excess of the FXR$_{short}$ peptide substrate (ERMRPFKRQGSVRRRV) for an hour on ice. Crystallization was performed using hanging drop method with 1:1 ratio of protein to precipitant at 20 °C. The protein at crystallized in 32% Peg 2000 MME and 0.08 M KSCN and was frozen in cryo-protectant containing 32% Peg 2000 MME, 0.08 M KSCN, and 20% glycerol. Data were collected at Diamond IO4. Data were processed using D*Trek (Pflugrath, 1999), and scaled with Scala and Pointless (Collaborative Computational Project-Number 4, 1994). The data was solved and refined using Phaser and Phenix as described above.

Cell culture and transfection

HCT-116 cells grown in McCoy's 5A medium containing 10% bovine foetal calf serum and penicillin/streptomycin (Invitrogen) were transfected (10 μg portion of DNA or 5 μg + 5 μg portions of DNA for co-transfections) using Fugene HD (Roche) according to the manufacturer's instructions. The cells were then grown in normal medium for 36 h.

Antibodies

The following antibodies were used for immunoblotting: mouse monoclonal anti-PKCι (recognition for human PKCι), mouse monoclonal anti-Myc (9E10), rabbit polyclonal anti-Par3 (Millipore), rabbit polyclonal anti-GFP antibody (Santa Cruz). Anti-phospho-S827 Par3 antibody was raised in-house using CQREGFGRQSMSEKRTKQ as an antigen, lacking the F-X-R site of Par3$_{CR3}$. For Western blots using the phospho-specific pPAR3 Ab, 100ng/ml of the de-phospho peptide to compete out during the primary Ab incubation.

Plasmids and mutagenesis

pEGFP-PKCi-WT, pEGFP-PKCi-DD/AA (D339A/D382A); pK-myc-Par3-WT (addgene, plasmid 19388), pK-myc-Par3-AXA (F818A/R820A) and pK-myc-Par3-A (S827A) included human PKCι and Par3 cDNAs. Mutagenesis of PKCι and Par3 was performed using the QuikChange Site-Directed Mutagenesis Kit (Stratagene) according to the manufacturer's instructions. The nucleotide changes were verified by in-house DNA sequencing.

Immunoprecipitation

After 36 h, transfected HCT-116 cells were lysed in lysis buffer LB (1% Triton X-100, 20 mM Tris–HCl, pH 8, 130 mM NaCl, 1 mM dithiothreitol (DTT), 10 mM sodium fluoride, complete EDTA-free protease inhibitor cocktail (Roche), phosphatase inhibitor cocktails (set II+IV, Calbiochem)). After centrifugation (13,000*g*, 4 °C, 10 min), soluble proteins were pre-cleared then incubated with anti-GFP magnetic beads (GFP-TRAP; Chromotek) or anti-Myc agarose beads (sigma) for 90 min or 2 h respectively at 4 °C. Beads were washed 5 times with lysis buffer (containing 260 mM NaCl), then the bound proteins were eluted in Laemmli sample buffer, resolved by SDS–PAGE and analysed by immunoblotting.

Immunoblotting

For immunoblotting, proteins from samples in Laemmli sample buffer were separated using precast NuPAGE 4-12% Bis-Tris gels (Invitrogen), transferred to nitrocellulose membranes (PROTRAN, Whatman), and incubated with antibodies diluted in Tris-buffered saline containing 5% non-fat milk and 0.1% Tween-20 for immunodetection.

Dimethyl labelling and quantification of Bazooka wild type and mutant S980 phosphorylation in S2 cell extracts.

After SDS-PAGE, in-gel stable isotope dimethyl labelling was performed according to published protocols (Boersema et al., 2009). The heavy reaction was performed using $^{13}CD_2O$ formaldehyde creating a mass difference of 6 Daltons per primary amine group between heavy and light dimehtylated peptides. After extensive washing of gel pieces, the in-gel dimethylated proteins were then subjected to overnight in-gel trypsin digestion at 37°C. The following day peptides were extracted and subjected to another round of reductive dimethylation reactions aimed at methylating peptide n-

termini. Peptide mixtures were acidified and prepared for LC-MS analysis using an Ulimate3000 HPLC coupled to a Q-Exactive mass spectrometer (Thermofisher). A targeted scan was performed for the S980 containing peptides and this was alternated with top10 data-dependent acquisition scan. Mascot generated DAT files were converted to Skyline compatible biblio.spec libraries and heavy and light peak areas were extracted by Skyline software version 2.5.0.6079 (MacLean et al., 2010).

Drosophila genetics and oligonucleotides

Expression of *UAS*-driven transgenes in follicle cells was achieved with the GR1.Gal4 line. *UAS.GFP-Baz* lines were constructed by mutagenising the full-length Baz cDNA in pDONR, followed by transfer to the pPGW (pUASP-EGFP-Gateway) vector for transgenesis (Bestgene, Inc). The *UAS.GFP-BazS980E* line was a gift from F. Pichaud. The following primers were used for mutagenesis:

Wild-type *Drosophila* Bazooka:

S L E T N S G V E H **F** S **R** D A L G R R **S**
AGCTTGGAGACAAACTCGGGCGTGGAGCAT**TTC**TCG**CGC**GATGCTTTGGGACGACGC**AG**
C
I S E K H H A A L D A R E T G T Y Q R N
ATCTCTGAGAAGCACCATGCGGCGCTGGATGCCCGCGAAACTGGCACCTATCAGCGGAAT

The FXR mutant:

S L E T N S G V E H **A** S **A** D A L G R R **S**
AGCTTGGAGACAAACTCGGGCGTGGAGCAT**GCC**TCG**GCC**GATGCTTTGGGACGACGC**AG**
C
I S E K H H A A L D A R E T G T Y Q R N
ATCTCTGAGAAGCACCATGCGGCGCTGGATGCCCGCGAAACTGGCACCTATCAGCGGAAT

5' CTCGGGCGTGGAGCAT**GCC**TCG**GCC**GATGCTTTGGGACGA 3' sense
5' TCGTCCCAAAGCATCGGCCGAGGCATGCTCCACGCCCGAG 3' anti-sense

The S980A mutant:

S L E T N S G V E H **F** S **R** D A L G R R **A**
AGCTTGGAGACAAACTCGGGCGTGGAGCAT**TTC**TCG**CGC**GATGCTTTGGGACGACGC**GC**
C
I S E K H H A A L D A R E T G T Y Q R N
ATCTCTGAGAAGCACCATGCGGCGCTGGATGCCCGCGAAACTGGCACCTATCAGCGGAAT

5' GCTTTGGGACGACGC**GCC**ATCTCTGAGAAGCAC 3' sense
5' GTGCTTCTCAGAGATGGCGCGTCGTCCCAAAGC 3' anti-sense

The FXR / S980A mutant:

S L E T N S G V E H **A** S **A** D A L G R R **A**
AGCTTGGAGACAAACTCGGGCGTGGAGCAT**GCC**TCG**GCC**GATGCTTTGGGACGACGC**GC**
C
I S E K H H A A L D A R E T G T Y Q R N
ATCTCTGAGAAGCACCATGCGGCGCTGGATGCCCGCGAAACTGGCACCTATCAGCGGAAT

CGGGCGTGGAGCAT**GCC**TCG**GCC**GATGCTTTGGGACGACGC**GCC**ATCTCTGAGAAGCAC sense
GTGCTTCTCAGAGATGGCGCGTCGTCCCAAAGCATCGGCCGAGGCATGCTCCACGCCCG anti-s

Wild-type *Drosophila* Bazooka:
I S E **K H** H A A L D
5' ATCTCTGAG**AAGCA**CCATGCGGCGCTGGAT 3'

The KH mutant:
I S E **A A** H A A L D
5' GACGCAGCATCTCTGAGGCGGCCCATGCGGCGCTGGATG 3' sense
3' CATCCAGCGCCGCATGGGCCGCCTCAGAGATGCTGCGTC 3' anti-sense

Drosophila antibodies and immunohistochemistry

Ovaries were dissected in PBS, fixed for 20 mins in 4% paraformaldehyde in PBS, washed for 30 minutes in PBS/0.1% Triton X-100 (PBST) and blocked for 15 minutes in 5% normal goat serum/PBST (PBST/NGS). Primary antibodies were diluted in PBST/NGS and samples were incubated overnight at 4 °C. Either optical cross-sections through the middle of egg chambers or apical sections of the follicular epithelium are shown.

Primary antibodies used were: rabbit anti-PKCζ (C-20) (1:250, Santa Cruz), mouse anti-E-cad (1:100 DSHB), mouse anti-Dlg (1:250, DSHB), rabbit anti-Bazooka (1:250, A.Wodarz). Secondary antibodies (all from Molecular Probes, Life) were used at 1:500 for 2 hours prior to multiple washes in PBST and staining with DAPI at 1 μg/ml for 10-30 mins prior to mounting on slides in Vectashield (Vector labs). Images were taken with a Leica SP5 confocal and processed with Adobe Photoshop.

Supplementary References

Adams, P.D., Afonine, P.V., Bunkoczi, G., Chen, V.B., Davis, I.W., Echols, N., Headd, J.J., Hung, L.W., Kapral, G.J., Grosse-Kunstleve, R.W.*, et al.* (2010). PHENIX: a comprehensive Python-based system for macromolecular structure solution. Acta Crystallogr D Biol Crystallogr *66*, 213-221.

Collaborative Computational Project-Number 4 (1994). The CCP-4 suite: programs for protein crystallography. Acta Crystallogr D *50*, 760-763.

Emsley, P., and Cowtan, K. (2004). Coot: model-building tools for molecular graphics. Acta Crystallogr D *60*, 2126-2132.

Guettler, S., Vartiainen, M.K., Miralles, F., Larijani, B., and Treisman, R. (2008). RPEL motifs link the serum response factor cofactor MAL but not myocardin to Rho signaling via actin binding. Mol Cell Biol *28*, 732-742.

Heyduk, T., and Lee, J.C. (1990). Application of fluorescence energy transfer and polarization to monitor Escherichia coli cAMP receptor protein and lac promoter interaction. Proc Natl Acad Sci U S A *87*, 1744-1748.

Kabsch, W. (2010a). Integration, scaling, space-group assignment and post-refinement. Acta Crystallogr D Biol Crystallogr *66*, 133-144.

Kabsch, W. (2010b). XDS. Acta Crystallogr D Biol Crystallogr *66*, 125-132.

MacLean, B., Tomazela, D.M., Shulman, N., Chambers, M., Finney, G.L., Frewen, B., Kern, R., Tabb, D.L., Liebler, D.C., and MacCoss, M.J. (2010). Skyline: an open source document editor for creating and analyzing targeted proteomics experiments. Bioinformatics *26*, 966-968.

McCoy, A.J., Grosse-Kunstleve, R.W., Adams, P.D., Winn, M.D., Storoni, L.C., and Read, R.J. (2007). Phaser crystallographic software. J Appl Crystallogr *40*, 658-674.

Morais-de-Sa, E., Mirouse, V., and St Johnston, D. (2010). aPKC phosphorylation of Bazooka defines the apical/lateral border in Drosophila epithelial cells. Cell *141*, 509-523.

Nishikawa, K., Toker, A., Johannes, F.J., Songyang, Z., and Cantley, L.C. (1997). Determination of the specific substrate sequence motifs of protein kinase C isozymes. J Biol Chem *272*, 952-960.

Pflugrath, J.W. (1999). The finer things in X-ray diffraction data collection. Acta Crystallogr D Biol Crystallogr *55*, 1718-1725.
