# Supplementary material for: aPKC Inhibition by Par3 CR3 Flanking Regions Controls Substrate Access and Underpins Apical-Junctional Polarization
Source: Dev Cell. 2016 Aug 22;38(4):384–98. doi: 10.1016/j.devcel.2016.07.018 (PMC4998004; doi:10.1016/j.devcel.2016.07.018)
Supplement: Document S2. Article plus Supplemental Information [file mmc2.pdf]

Article

Developmental Cell

# aPKC Inhibition by Par3 CR3 Flanking Regions Controls Substrate Access and Underpins Apical-Junctional Polarization

## Graphical Abstract

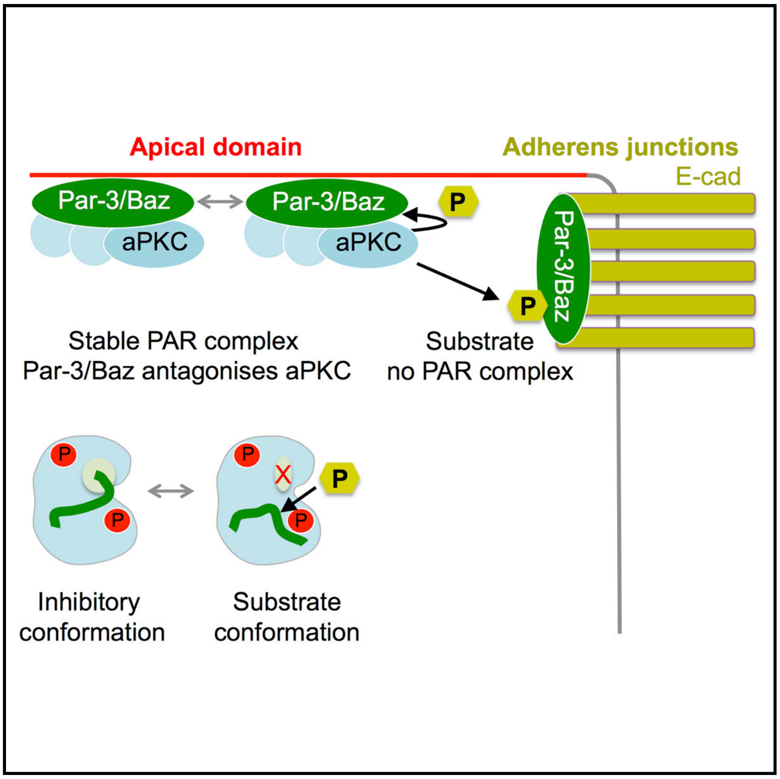


## Authors

Erika V. Soriano, Marina E. Ivanova, Georgina Fletcher, ..., Peter J. Parker, Barry J. Thompson, Neil Q. McDonald

## Correspondence

barry.thompson@crick.ac.uk (B.J.T.), neil.mcdonald@crick.ac.uk (N.Q.M.)

## In Brief

Par3 is required for aPKC membrane recruitment, yet it polarizes to adherens junctions upon phosphorylation. Soriano et al. show that Par3 antagonizes active aPKC kinase by separating crucial N-lobe contacts. Disrupting high-affinity Par3 contacts switches it to an efficient aPKC substrate and polarizes Par3/Bazooka from apical domains to adherens junctions.

## Highlights

- Sequences flanking the Par3 CR3 consensus PKC site cooperate to inhibit aPKC
- A Par3 CR3 inhibitory arm disrupts aPKC P-loop/$\alpha$B/$\alpha$C contacts and $\alpha$C-helix position
- Mutating either CR3 arm switches Par3 into an efficient aPKC substrate in vitro
- Equivalent Bazooka substitutions alter its apical localization to AJs in vivo

## Accession Numbers

5LI1
5LIH
5LI9

Soriano et al., 2016, Developmental Cell *38*, 384–398
August 22, 2016 © 2016 The Author(s). Published by Elsevier Inc.
http://dx.doi.org/10.1016/j.devcel.2016.07.018

CellPress

Developmental Cell
Article

# aPKC Inhibition by Par3 CR3 Flanking Regions Controls Substrate Access and Underpins Apical-Junctional Polarization

Erika V. Soriano,[1] Marina E. Ivanova,[1] Georgina Fletcher,[2] Philippe Riou,[3] Philip P. Knowles,[1] Karin Barnouin,[4] Andrew Purkiss,[1] Brenda Kostelecky,[1] Peter Saiu,[1] Mark Linch,[3] Ahmed Elbediwy,[2] Svend Kjær,[6] Nicola O'Reilly,[5] Ambrosius P. Snijders,[4] Peter J. Parker,[3,7] Barry J. Thompson,[2,*] and Neil Q. McDonald[1,8,9,*]
[1]Structural Biology
[2]Epithelial Biology
[3]Protein Phosphorylation Laboratories
[4]Protein Analysis
[5]Peptide Chemistry
[6]Protein Purification Facilities
The Francis Crick Institute, 44 Lincoln's Inn Fields, London WC2A 3LY, UK
[7]Division of Cancer Studies, King's College London, London SE1 1UL, UK
[8]Institute of Structural and Molecular Biology, School of Biological Science, Birkbeck College, Malet Street, London WC1E 7HX, UK
[9]Lead Contact
*Correspondence: barry.thompson@crick.ac.uk (B.J.T.), neil.mcdonald@crick.ac.uk (N.Q.M.)
http://dx.doi.org/10.1016/j.devcel.2016.07.018

## SUMMARY

**Atypical protein kinase C (aPKC) is a key apical-basal polarity determinant and Par complex component. It is recruited by Par3/Baz (Bazooka in *Drosophila*) into epithelial apical domains through high-affinity interaction. Paradoxically, aPKC also phosphorylates Par3/Baz, provoking its relocalization to adherens junctions (AJs). We show that Par3 conserved region 3 (CR3) forms a tight inhibitory complex with a primed aPKC kinase domain, blocking substrate access. A CR3 motif flanking its PKC consensus site disrupts the aPKC kinase N lobe, separating P-loop/αB/αC contacts. A second CR3 motif provides a high-affinity anchor. Mutation of either motif switches CR3 to an efficient in vitro substrate by exposing its phospho-acceptor site. In vivo, mutation of either CR3 motif alters Par3/Baz localization from apical to AJs. Our results reveal how Par3/Baz CR3 can antagonize aPKC in stable apical Par complexes and suggests that modulation of CR3 inhibitory arms or opposing aPKC pockets would perturb the interaction, promoting Par3/Baz phosphorylation.**

## INTRODUCTION

Epithelial tissues are composed of sheets of polarized cells that are connected by adherens junctions (AJs) (Laprise and Tepass, 2011; St Johnston and Ahringer, 2010; Suzuki and Ohno, 2006). The plasma membrane of epithelial cells is segregated into apical and basolateral domains, with a prominent belt of AJs located at the interface of these two domains (Figures S1A and S1B). The atypical protein kinase C (aPKC in *Drosophila* or PKCι/PKCζ isozymes in mammals), its binding partner Par6, and the small guanosine triphosphatase Cdc42 are three essential determinants of apical membrane identity in both *Drosophila* and mammals (Fletcher et al., 2012; Harris and Tepass, 2008; Hutterer et al., 2004; Izumi et al., 1998; Joberty et al., 2000; Lin et al., 2000; Wodarz et al., 2000). The aPKC-Par6-Cdc42 assembly can form a larger stable complex with Par3/Baz (Bazooka [Baz] in *Drosophila*) (known as the Par complex) at the apical membrane (Izumi et al., 1998; Joberty et al., 2000; Lin et al., 2000; Wodarz et al., 2000). Association of Par3 with the basolateral membrane is prevented by phosphorylation of its lipid-binding domain by the basolateral kinase Par1 (Benton and St Johnston, 2003b).

Importantly, a distinct pool of Par3/Baz can also segregate away from apical aPKC-Par6-Cdc42 and localize to AJs (Morais-de-Sa et al., 2010; Walther and Pichaud, 2010). The role of Par3/Baz at AJs is thought to be essential as it involves defining the position of AJs during the establishment of epithelial polarity (Wang et al., 2012b) and possibly also remodeling of AJs as tissues undergo morphogenetic change (Walther and Pichaud, 2010). The regulation of this switch of Par3/Baz subcellular localization from the apical membrane to AJs has been shown in *Drosophila* to be dependent on aPKC phosphorylating Par3/Baz on serine 980 in vivo (Morais-de-Sa et al., 2010; Walther and Pichaud, 2010). This site is within a consensus PKC phosphorylation R-X-S-Ψ motif and is equivalent to serine 827 of human Par3 (Figure S1D), both sites map to Par3/Baz conserved region 3 (CR3), a site of regulated protein interaction (Nagai-Tamai et al., 2002). However, how Par3/Baz switches from being a stable binding partner of aPKC in the Par complex to being a substrate of aPKC that segregates away from the Par complex remains unclear. In mammalian cells, a similar conundrum exists whereby Par3 is critical for the recruitment of PKCι to the apical membrane and is known to be an in vivo substrate of PKCι, but loss of Par3 in transformed epithelial cells can lead to PKCι activation

 © 2016 The Author(s). Published by Elsevier Inc.
This is an open access article under the CC BY license (http://creativecommons.org/licenses/by/4.0/).

and can result in breast tumorigenesis and metastasis (McCaffrey and Macara, 2009; McCaffrey et al., 2012).

One complication in understanding the role of Par3/Baz in *Drosophila* epithelia is the presence of another key apical determinant, Crumbs (Crb) (Tepass, 1996). Like Par3/Baz, Crb can localize apically in a complex with Stardust (Sdt) (Bilder et al., 2003; Roh et al., 2003; Tanentzapf and Tepass, 2003; Tepass, 1996) and aPKC-Par6-Cdc42 (called the Crb complex) (Fletcher et al., 2012; Harris and Tepass, 2008; Morais-de-Sa et al., 2010). Par3/Baz and Crb-Sdt can therefore act in a semi-redundant fashion to specify the apical domain in *Drosophila*, such that either Par3/Baz or Crb-Sdt is usually sufficient to maintain polarity in *Drosophila* (Fletcher et al., 2012; Tanentzapf and Tepass, 2003). Similarly, Willin, a FERM-domain protein, has been implicated in another Par3-independent apical domain recruitment mechanism for Par6-aPKC (Ishiuchi and Takeichi, 2011). The presence of Crb has been shown to promote Par3/Baz localization to AJs (Morais-de-Sa et al., 2010; Walther and Pichaud, 2010). However, in the absence of Crb, some Par3/Baz can still be phosphorylated by aPKC on S980 so that it localizes to AJs (Morais-de-Sa et al., 2010). These findings indicate that individual Par3/Baz molecules can localize either apically or junctionally without requiring any input from Crb. Thus, the paradoxical dual role of Par3/Baz as either a Par complex component or an aPKC substrate appears to be an emergent property of these molecules themselves, although it is still uncertain how this property arises.

aPKC isoforms PKCι and PKCζ have regulatory regions distinct from those of other PKC isozymes, but share a conserved catalytic protein kinase domain (Parker and Murray-Rust, 2004). They are not responsive to diacylglycerol and have less well-defined activators (Limatola et al., 1994). Like many protein kinases, activation of aPKC requires activation-loop phosphorylation and an αC-helix conformation compatible with Lys-Glu salt-bridge formation to bind ATP and serve to align residues within the R spine (Kornev et al., 2008). Functionally validated aPKC substrates include Par3, LLGL2, ROCK1, and MARK2, and the Hippo pathway component Kibra (Betschinger et al., 2005; Buther et al., 2004; Hurov et al., 2004; Ishiuchi and Takeichi, 2011). Sequences flanking the phospho-acceptor site in each aPKC substrate are rich in basic residues consistent with basophilic AGC kinase consensus sites derived from short peptide substrates (4–14 residues) (https://www.kinexus.ca). In these contexts aPKC phosphorylation inactivates substrates with basophilic membrane-binding motifs with embedded phosphorylation sites such that they are displaced from membranes (Bailey and Prehoda, 2015).

Here, we describe how Par3 CR3 recognizes and inhibits a nucleotide-occupied primed PKCι. Two Par3 CR3 motifs flanking its PKC consensus site engage pockets within the PKCι kinase domain, one of which disrupts crucial N-lobe contacts required for catalytic activity. A second contact used by both aPKC inhibitors and substrates provides a high-affinity anchor point through a Phe-X-Arg motif. Together, both motifs cooperate to block aPKC substrate access and prevent phospho-transfer to Par3 CR3. Mutation of either motif switches Par3 from an inhibitor to an efficient substrate in vitro and redistributes equivalent Bazooka mutants to AJs in vivo. These data are consistent with high-affinity inhibitory interactions between Par3/Baz and aPKC preventing Par3/Baz phosphorylation and thereby promoting stable complex formation and apical localization. Modulation of the CR3 inhibitory arm by phosphorylation or engagement of the aPKC pocket by partner proteins would switch Par3/Baz to a more transient type of interaction, consequently enabling efficient phosphorylation of Par3/Baz by aPKC and subsequent relocalization to AJs.

## RESULTS

### The Par3 CR3 Region Inhibits Nucleotide-Bound Primed PKCι Kinase Domain through Two Flanking Arm Contacts

The human Par3 conserved region 3 (CR3, covering residues 816–834, defined hereafter as $Par3_{CR3}$) is able to bind to PKCι (Nagai-Tamai et al., 2002) and contains a phospho-acceptor site (P site) at residue serine 827 known to be phosphorylated by PKCι (Figures 1A and 1B). To characterize its interaction with PKCι we purified a "primed" active form of the human PKC-iota kinase domain (referred to as $PKCι_{KD}$-2P) and a partially primed low-activity form (referred to as $PKCι_{KD}$-1P), referring to the status of the two "priming" phosphorylation sites at pT412 and pT564 (Figures 1A and S2A–S2C). We then probed how efficiently they were able to phosphorylate $Par3_{CR3.}$ Surprisingly, we found that $Par3_{CR3}$ strongly inhibited the catalytic activity of $PKCι_{KD}$-2P in vitro and could competitively block phosphorylation of a model substrate peptide, with an apparent 50% inhibitory concentration ($IC_{50}$) of 0.45 ± 0.18 μM. In contrast, peptides from other known aPKC substrates such as Par1 were efficiently phosphorylated and were unable to inhibit (Figures 1B–1D). Using a fluorescence anisotropy assay, we found that the $Par3_{CR3}$ binds to $PKCι_{KD}$-2P with submicromolar affinity ($K_D$ of 0.47 ± 0.09 μM), as does an S827A mutant ($K_D$ of 0.97 ± 0.07 μM) (Figure 1E). $PKCι_{KD}$-2P is a good surrogate for an activated Par complex containing Par6-PKCι-Cdc42 complex that exhibits high activity in vitro and is also potently inhibited by $Par3_{CR3}$ (data not shown). In contrast, $PKCι_{KD}$-1P was not inhibited to the same extent and had a much lower affinity for $Par3_{CR3}$ (compare Figures 1D, S2D, and S2E). We conclude that a high-affinity $Par3_{CR3}$ targets $PKCι_{KD}$-2P and inhibits its catalytic activity.

To understand how $Par3_{CR3}$ could inhibit $PKCι_{KD}$-2P, we determined the 2.0-Å crystal structure of a longer Par3 peptide (residues 816–841) bound to $PKCι_{KD}$-2P and Mg-AMPPNP (adenylyl imidodiphosphate) (Figures 2A and S3A; Table 1). The $Par3_{CR3}$ peptide is well ordered in this structure and contains seven intramolecular hydrogen bonds (Figure S3B). It engages $PKCι_{KD}$-2P by adopting a "staple"-shaped conformation with two arms that flank the $S827^{Par3}$ phospho-acceptor site. Each arm binds in close proximity to opposite ends of the nucleotide, suggesting that recognition of aPKC is driven by nucleotide occupancy. The relative orientation of N and C lobes indicates a "closed" rather than "open" conformation. $Par3_{CR3}$ contacts extend from a pocket beneath the ribose-binding pocket of PKCι (site 1), across the G helix (site 2) through to the activation loop, αB and αC helices of the $PKCι_{KD}$-2P N lobe (site 3) (Figures 2A and 2B). A total surface area of more than 1,305 $Å^2$ is buried within the complex, consistent with a high-affinity inhibitory interaction. The nucleotide cleft is occupied by an Mg-AMPPNP

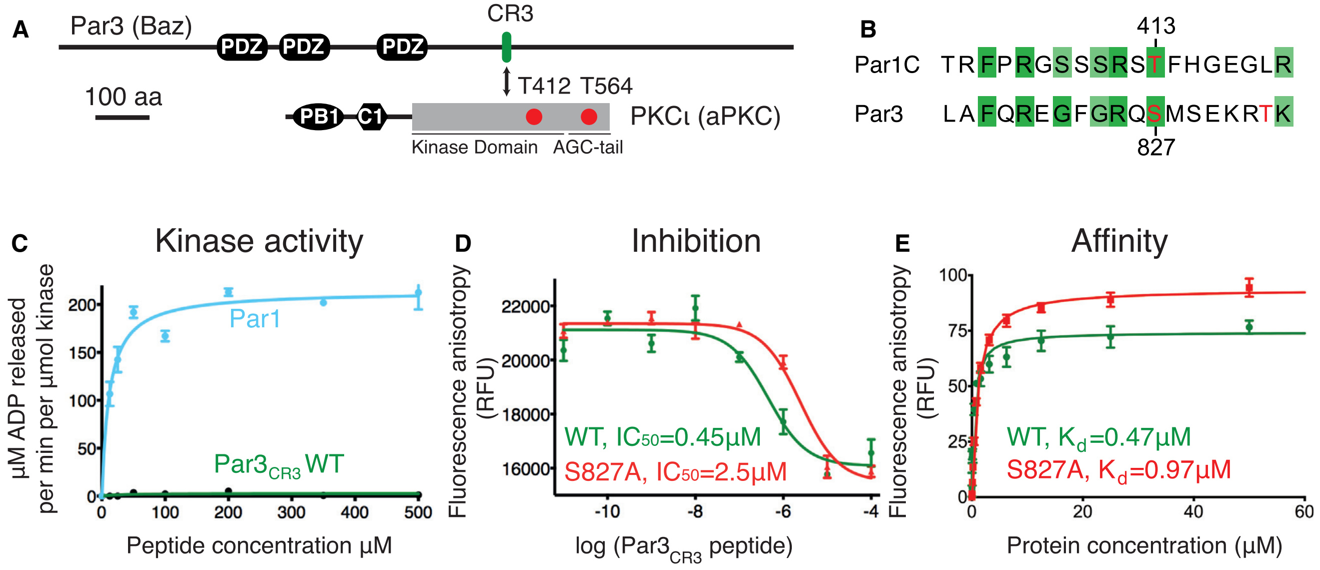


**Figure 1. Par3/Baz CR3-Mediated Inhibition of aPKC In Vitro**

(A) Domain structure of Par3 and PKCι and location of key phosphorylation sites in each. For more detail on aPKC and Par3/Baz subcellular localization and how aPKC phosphorylation of Par3/Baz switches Par3/Baz localization from the apical membrane to AJs, see Figure S1.

(B) Sequence alignment of human Par3 CR3 region with Par1 highlighting known phosphorylation sites (red).

(C) Par3$_{CR3}$ inhibits PKCι$_{KD}$-2P catalytic activity in an in vitro kinase assay, whereas a Par1-derived peptide is a substrate.

(D) The IC$_{50}$ curves for Par3$_{CR3}$.

(E) Affinity of fluorescein-labeled Par3$_{CR3}$ for PKCι$_{KD}$-2P measured by fluorescence anisotropy.

RFU, relative fluorescence units; WT, wild-type. Data are plotted as mean ± SEM. See also Figure S2 for purification and further characterization of PKCι$_{KD}$-2P.

nucleotide (Figure 2C). The conserved nucleotide-coordinating lysine (K283$^{PKC\iota}$) forms a salt bridge with the conserved αC-helix glutamate (E302$^{PKC\iota}$) side chain found in many active kinase conformers (Kornev et al., 2008). The terminal γ-phosphate of AMPPNP is not observed in the structure, consistent with AMPPNP being rapidly hydrolyzed under the crystallization conditions (see Experimental Procedures). A magnesium ion, equivalent to Mg2 of PKA, is present, bridging both the α and β phosphates of AMPPNP (Adams and Taylor, 1993; Zheng et al., 1993). The Mg1 ion is not present, as frequently found in ADP-complexed AGC kinase structures.

The amino-terminal part of Par3$_{CR3}$ binds to site 1 (PKCι$_{KD}$-2P kinase C lobe) through an F-X-R motif at positions −9 (F−9) and −7 (R−7) defined relative to the phospho-acceptor (P site) at serine 0 (equivalent to S827 of human Par3). F−9 lies deep within a hydrophobic cleft formed by M341$^{PKC\iota}$, M344$^{PKC\iota}$, and L381$^{PKC\iota}$ beneath the nucleotide pocket (Figure 2C). In addition, the side chain of R−7 forms a salt bridge to D339$^{PKC\iota}$, just beneath the ribose ring of the AMPPNP, while that of R−2 engages conserved residues Y419$^{PKC\iota}$ and E445$^{PKC\iota}$ (Figure 2C). As this motif does not appear to directly perturb aPKC catalytic residues, we refer to this element hereafter as the "affinity arm" of Par3$_{CR3}$ (Figure 2C).

From site 1, the Par3$_{CR3}$ backbone adopts two consecutive type II reverse turns with positive phi-main-chain angles at E−6 and G−3. This leads into site 2, positioned to contact the G helix through residue F−4 that displaces and disorders the aPKC-specific kinase insert (residues 455$^{PKC\iota}$ to 466$^{PKC\iota}$). The phospho-acceptor serine-0 hydroxyl hydrogen bonds to side chains of D378$^{PKC\iota}$, K380$^{PKC\iota}$, and T416$^{PKC\iota}$, preventing a catalytically competent orientation for nucleophilic attack on the ATP γ-phosphate. Glycine-rich loop residues S264$^{PKC\iota}$ and Y265$^{PKC\iota}$ side chains contact the CR3 main-chain atoms near the P site, as does the activation-loop main chain, to orient the Par3$_{CR3}$ peptide and position the M+1 side chain into the known P+1 AGC kinase hydrophobic pocket (Figure 2C) (Pearce et al., 2010).

Site 3 contains carboxy-terminal flanking residues to the P site stretching from S+2 to T+6. We define this portion of Par3$_{CR3}$ as the "inhibitory arm," as it directly perturbs an active PKCι$_{KD}$-2P N-lobe conformation (discussed later). Residue K+4 directly contacts pT412$^{PKC\iota}$ of the activation loop enhancing the recognition of mature, primed PKC ι$_{KD}$-2P, but importantly not a partially primed PKCι$_{KD}$-1P. Crucially, the R+5 side chain is buried within a hydrophobic pocket beneath the regulatory αC helix. The pocket is lined by side chains from Y265$^{PKC\iota}$ on the glycine loop and W298$^{PKC\iota}$ of the αC helix, each making π-stacking interactions with the guanidino group of R+5 (Figures 2C and 3A). Both aromatic side chains are unique to aPKC isozymes from *Drosophila* to mammals. Finally, T+6 (equivalent to T833$^{Par3}$, a known ROCK-driven phosphorylation site discussed later) lies adjacent to an acidic patch within the αB helix making side-chain and main-chain contacts to D295$^{PKC\iota}$ and a Mg ion (Figures 2C and 3A). Overall, the structure reveals that the Par3$_{CR3}$ clamp involves an "inhibitory arm" and an "anchoring arm," which together recognize and inhibit a nucleotide-bound PKCι$_{KD}$-2P conformer.

## Comparison of Par3$_{CR3}$-Inhibited PKCι Complex with an Active PKCι Conformer Reveals the Basis for Inhibition

To fully understand how Par3$_{CR3}$ inhibits PKCι and disrupts its activated state, we determined the structure of an "active"

**Table 1. Data Collection and Refinement Statistics**

| | PKCι$_{KD}$-2P/Par3 CR3 Peptide/ Mg-AMPPNP | PKCι$_{KD}$-2P/AMPPCP | PKCι$_{KD}$-2P/Mn-ADP/AlF$_3$/FXR-Short Peptide |
|---|---|---|---|
| Data Collection | | | |
| Space group | $P3_121$ | $P2_12_12_1$ | $P2_12_12_1$ |
| Cell Dimensions | | | |
| *a*, *b*, *c* (Å) | 82.0, 82.0, 90.8 | 61.1, 65.1, 87.4 | 79.0, 84.2, 111.8 |
| α, β, γ (°) | 90, 90, 120 | 90, 90, 90 | 90, 90, 90 |
| Resolution (Å) | 45.45–1.95 (2.06–1.95) | 52.23–1.79 (1.84–1.79) | 67.28–3.25 (3.43–3.25) |
| Completeness (%) | 99.6 (997.5) | 100 (100) | 99.6 (99.9) |
| Multiplicity | 8.2 (7.0) | 9.5 (9.6) | 3.8 (3.6) |
| $R_{meas}$ (%)$_{pim}$ | 9.0 (64.0) | 17.3 (200) | 20.0 (55.1) |
| $R_{p.i.m.}$ (%)$_{pim}$ | 3.1 (23.3) | 5.6 (63.4) | 9.9 (27.5) |
| $<I>/<\sigma I>$ | 15.0 (3.0) | 8.2 (1.4) | 6.3 (2.5) |
| Total no. of observations | 216,286 (25,839) | 323,684 (23,802) | 46,484 (6,806) |
| Total no. unique | 26,242 (3,689) | 33,998 (2,474) | 1,200 (1,753) |
| Structure Refinement | | | |
| $Z_a$ | 1 | 1 | 2 |
| Reflections | 25,607 | 33,825 | 12,145 |
| $R_{work}$ (%) | 15.0 | 18.8 | 25.66 |
| $R_{free}$ (%) | 21.7 | 23.1 | 28.36 |
| No. of protein atoms | A = 2,719 | A = 2,701 | A = 2,527, B = 2,489, F = 100, G = 76 |
| No. of ligand atoms | B = 154, D = 66 | B = 48 | C = 27, D = 27, other = 27 |
| No. of solvent atoms | C = 2, E = 170, F = 14 | C = 21, D = 244, E = 5, F = 8, G = 18, I = 8 | E = 25 |
| Mean *B* Factor | | | |
| Protein | A = 26.1 | A = 23.7 | (A, B, F, G) = 44.00 |
| Ligand | B = 35.0, D = 29.5 | B = 28.0 | all (non-water) = 41.7 |
| Solvent | C = 45.6, E = 38.1, F = 45.5 | C = 61.3, D = 35.7, E = 65.3, F = 58.4, G = 46.1, I = 52.9 | E = 32.0 |
| RMSD bonds (Å), angles (°) | 0.008, 1.100 | 0.004, 0.765 | 0.003, 0.733 |
| Ramachandran Plot (%) | | | |
| Favored | 98.0 | 97.6 | 94.5 |
| Allowed | 2.0 | 2.1 | 5.4 |
| Outliers | 0.0 | 0.3 | 0.15 |
| | where<br>A = protein<br>B = peptide<br>C = $K^+$ ion<br>D = AMPPNP<br>E = water<br>F = glycerol | where<br>A = protein<br>B = AMPPCP<br>C = formate<br>D = water<br>E = imidazole<br>F = MPD<br>G = PEG<br>I = acetate | where<br>A, B = protein<br>F, G = peptide<br>C, D = ADP<br>E = water |

RMSD, root-mean-square deviation; PEG, polyethylene glycol; MPD, 2-methyl-2,4-pentanediol.

conformer of PKCι for comparison (Figure 3B). Previous structures of PKCι kinase domain (PDB: 3A8W and 4DC2) (Takimura et al., 2010; Wang et al., 2012a) exhibited either a disordered or displaced αB-αC loop (Figures 3C and 3D). We captured an active mature PKCι conformation bound to the ATP analog 5′-(β,γ-adenylyl methylene)diphosphonate (AMPPCP) at 1.8 Å (Figure S3C and Table 1). This analog was resistant to hydrolysis compared with AMPPNP. The structure has an ordered αB-αC loop and reveals side-chain contacts between Y265$^{PKCι}$ of the P loop and D295$^{PKCι}$ of the αB-αC loop. This interaction stabilizes Y265$^{PKCι}$ side-chain stacking with a rotamer of W298$^{PKCι}$ from the αC helix (Figure 3B). Other PKC isoform structures have a phenylalanine and cysteine, respectively, at these positions (Grodsky et al., 2006; Leonard et al., 2011; Xu et al., 2004). Structural comparisons suggest that Par3$_{CR3}$ inhibitory arm not only separates P-loop contacts with αB-αC loop/αC helix but also

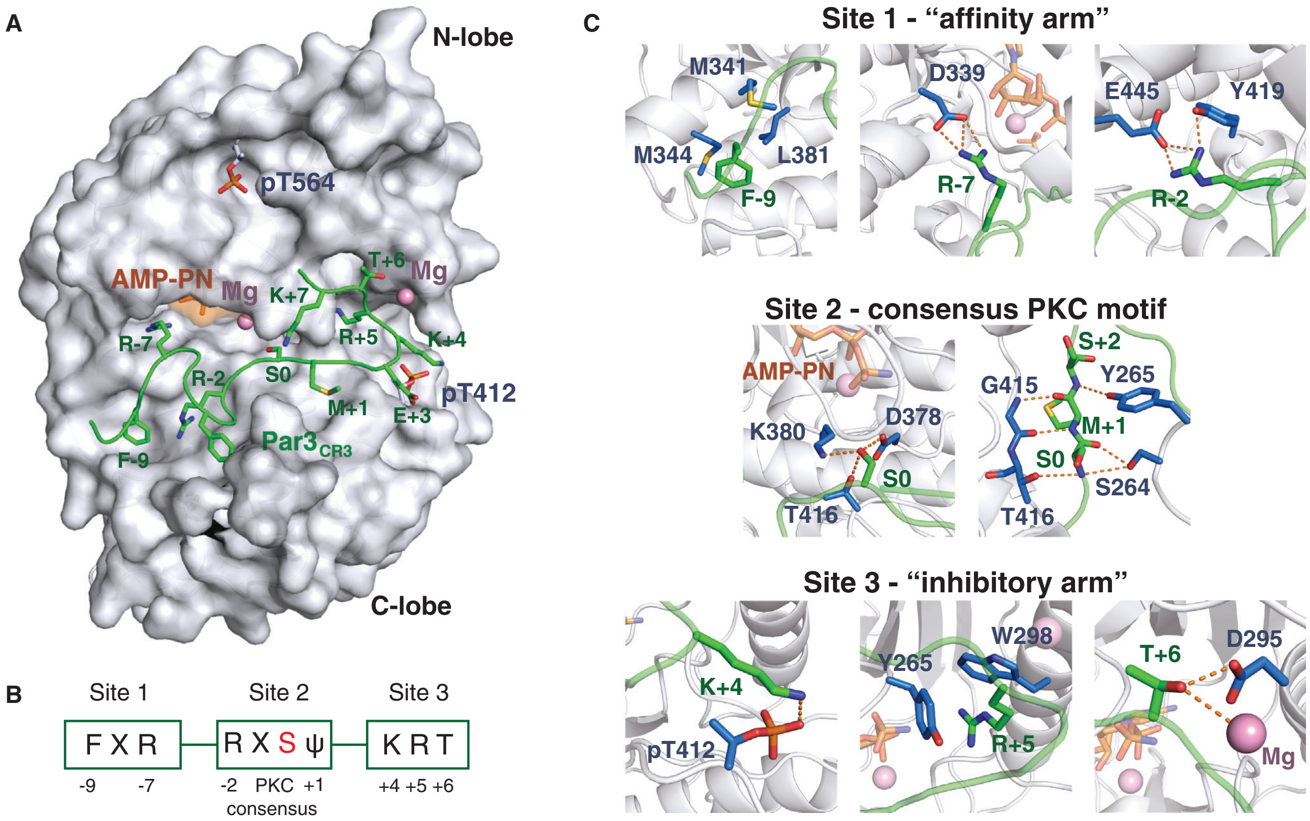


**Figure 2. Structural Basis for Par3/Baz CR3-Mediated Inhibition of aPKC**

(A) Overall structure of PKCι$_{\mathrm{KD}}$-2P (gray surface) bound to Par3$_{\mathrm{CR3}}$ (green stick) with Mg-AMP-PNP shown as an orange surface and priming sites at T564 and T412 indicated.

(B) Schematic representation of three sites of contacts between Par3$_{\mathrm{CR3}}$ and PKCι$_{\mathrm{KD}}$-2P. The known phospho-acceptor site at serine 0 is shown in red.

(C) Close up of the contacts between Par3$_{\mathrm{CR3}}$ peptide (green) and PKCι$_{\mathrm{KD}}$-2P (residues making contacts shown in blue). Hydrogen bonds between side chains or main-chain atoms are shown as dashed red lines. Pink spheres represent magnesium ions.

See also Figures S3A and S3B for refined Par3$_{\mathrm{CR3}}$ electron density and intramolecular hydrogen bonds within Par3$_{\mathrm{CR3}}$.

hijacks Y265$^{\mathrm{PKC}\iota}$ and S264$^{\mathrm{PKC}\iota}$ side chains to directly form hydrogen bonds with CR3 main-chain atoms. Comparing the Par3$_{\mathrm{CR3}}$ inhibitory complex with 1ATP (PKA bound to Mg-ATP and PKI peptide) suggests that the R+5 side-chain guanidine group lies close to the Mg2 ion of an active kinase conformation (Adams and Taylor, 1993; Zheng et al., 1993), indicating another layer of Par3$_{\mathrm{CR3}}$ disruption of an active PKCι conformation. Furthermore, T+6 (equivalent to T833$^{\mathrm{Par3}}$), which makes direct contact with D295$^{\mathrm{PKC}\iota}$, is a phospho-acceptor site targeted by the ROCK kinase, leading to a disruption of PKCι interaction with Par3 (Nakayama et al., 2008). This would predict, based on our structural comparison, that modulation of the "inhibitory arm" of Par3$_{\mathrm{CR3}}$ by ROCK kinase phosphorylation, or inaccessibility of the pocket to which it binds, could influence whether Par3 can inhibit PKCι or engages it as a substrate.

## A Shared High-Affinity Anchor Motif Used by aPKC Substrates and Inhibitors

Our Par3$_{\mathrm{CR3}}$-PKCι$_{\mathrm{KD}}$-2P inhibitory complex differs significantly from a previous structure of an ATP-binding deficient and partially primed PKCι K283R mutant (PKCι$_{\mathrm{KD}}$-1P, PDB: 4DC2) bound to Par3$_{\mathrm{CR3}}$ (Wang et al., 2012a). In the absence of nucleotide, Par3$_{\mathrm{CR3}}$ residues +3 to +7 were disordered and, therefore, the CR3 region is lacking inhibitory site 3 contacts (Wang et al., 2012a). Consistent with this, Par3$_{\mathrm{CR3}}$ is unable to potently inhibit the partially primed PKCι$_{\mathrm{KD}}$-1P or bind with high affinity (Figures S2D, S2E, and S4B). We present evidence that our Par3$_{\mathrm{CR3}}$-PKCι$_{\mathrm{KD}}$-2P structure represents a Par3$_{\mathrm{CR3}}$-mediated inhibitory complex of mature PKCι. However, the structure reported by Wang et al. (2012a) most likely resembles a weaker and transient Par3-PKCι interaction relevant to a protein kinase-substrate interaction (Figure 4).

To explore and capture a substrate peptide bound to PKCι$_{\mathrm{KD}}$-2P, we used an artificial substrate (FKRQGSVRRR, referred to hereafter as F-X-R$_{\mathrm{short}}$ peptide) (Figures S4A–S4E). This efficient PKCι substrate closely resembles the aPKC consensus motif identified from screening randomly oriented peptide libraries by Cantley and co-workers (Nishikawa et al., 1997). We therefore determined a crystal structure for F-X-R$_{\mathrm{short}}$ bound to PKCι$_{\mathrm{KD}}$-2P in the presence of Mn-ADP and $AlF_3$, a transition-state analog (Figure S4E). Manganese ions corresponding to Mg1 and Mg2 ions are present in the structure, and the $AlF_3$ is positioned as expected to mimic the transition state for the $\gamma$-phosphate. Surprisingly, the structure revealed that the

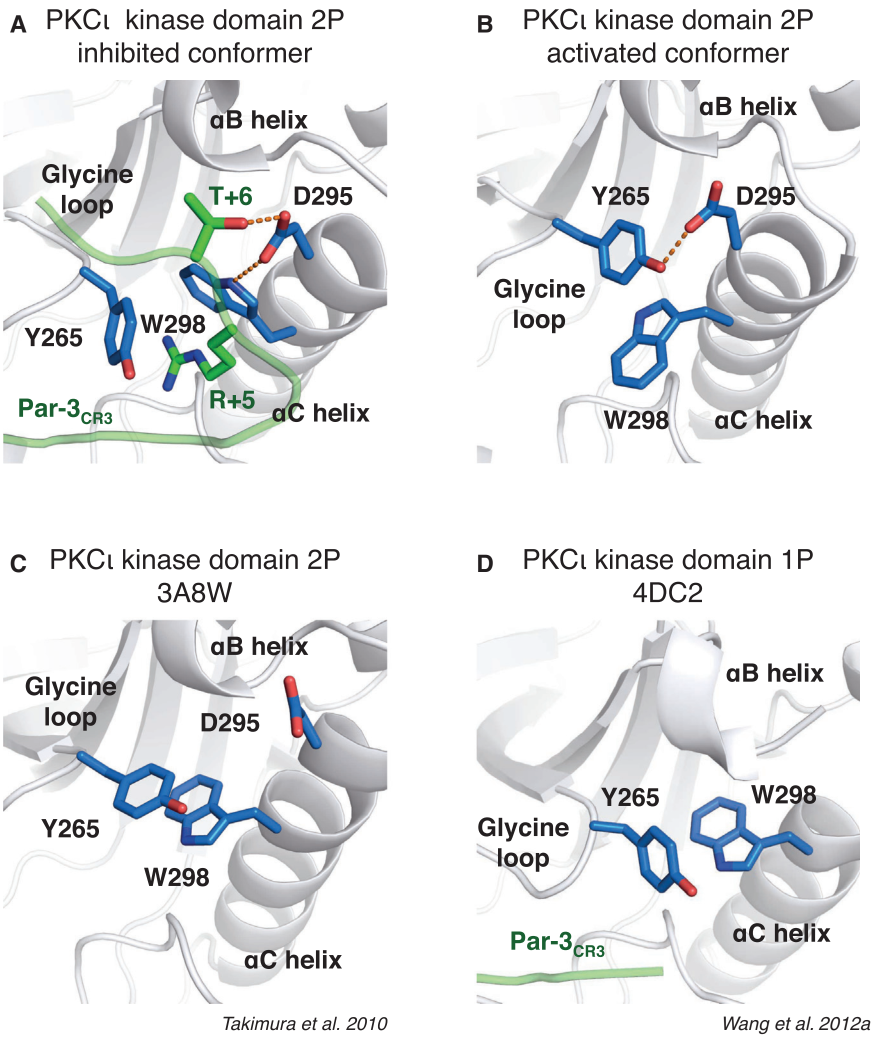


**Figure 3. Close-Up View of the Par3 CR3 Inhibitory Arm Pocket Bound to PKCι$_{KD}$-2P with Other PKCι$_{KD}$ Structures**

(A) Close up of the inhibitory R+5 hook of Par3$_{CR3}$ clamped by side chains Y265$^{PKCι}$ (P loop) and W298$^{PKCι}$ (αC helix) of PKCι$_{KD}$-2P (gray cartoon, major interaction residues are shown as blue sticks). Key structural features of the PKCι$_{KD}$-2P are labeled. Hydrogen bonds between key side chains are shown as dashed red lines.

(B) Close up of R+5 pocket in the active conformation of AMPPCP-bound PKCι$_{KD}$-2P structure. Hydrogen bonds between key side chains are shown as dashed red lines.

(C) Close up of R+5 pocket in the previously solved ATP-bound PKCι$_{KD}$-2P structure (PDB: 3A8W) (Takimura et al., 2010).

(D) Close up of PKCι$_{KD}$-1P K283R mutant within its ATP cleft (PDB: 4DC2) (Wang et al., 2012a).

See also Figure S3C for refined nucleotide analog electron density and Figure S3D for a comparison with a chemical inhibitor-induced PKCι$_{KD}$-2P conformer.

F-X-R motif at F−5 and R−3 engages precisely the same site 1 residue contacts (M341$^{PKCι}$, M344$^{PKCι}$ and L381$^{PKCι}$, and D339$^{PKCι}$) as the F−9 and R−7 contacts used by Par3$_{CR3}$, despite their different position in the primary sequence (Figure S4F). Moreover, the R−3 side chain directly makes a hydrogen bond with the ribose hydroxyl, perhaps sensing nucleotide occupancy. We also observe an R+2 side-chain bridging contact between phospho-T412$^{PKCι}$ and G398$^{PKCι}$ main-chain carbonyl, making two key hydrogen bonds with these groups. We note that many aPKC substrates have an R+2 side chain, suggesting that direct contact with a phosphorylated activation loop may reflect a common interaction made by aPKC substrates.

From the Par3$_{CR3}$ structure, it is evident that the F-X-R$_{short}$ peptide does not inhibit PKCι because it lacks a C-terminal inhibitory motif (Figures 4 and S5). Consistent with this, the Par1 peptide characterized as a good aPKC substrate also has an F-X-R anchor and a validated aPKC phosphorylation site, but lacks an obvious inhibitory motif (Figure 1C) (Hurov et al., 2004). In contrast, a Kibra-derived peptide (residues 919–978) containing a validated aPKC phosphorylation site has both an F-X-R motif anchor and a K-R inhibitory motif. As such it is able to potently inhibit PKCι in vitro (Figures S5A–S5C), consistent with reports of Kibra inhibiting aPKC kinase activity in epithelial cells (Yoshihama et al., 2011). Indeed a related peptide from WWC2 protein, a poorly characterized Kibra homolog, also inhibits PKCι in vitro (Figures S5A–S5C). Taken together, these data indicate that an F-X-R motif anchor amino-terminal to an aPKC phosphorylation site can be found in both aPKC substrates and inhibitors at variable lengths in their primary sequence from the phospho-acceptor site. Furthermore, the C-terminal inhibitory arm bearing a K-R-T motif is unique to aPKC protein inhibitors such as Par3 and Kibra, and can be predictive of an inhibitory function (WWC2).

## Manipulating Par3 CR3 Flanking Arms In Vitro Switches Par3 from an Inhibitor to an Efficient PKCι Substrate

Our results suggested that Par3 CR3 arms flanking the consensus PKC phosphorylation site cooperate to inhibit PKCι. To probe the individual contributions of each arm, we characterized Par3$_{CR3}$ substitutions at critical contact residues in the affinity arm and the inhibitory arm for their impact on Par3$_{CR3}$ affinity for PKCι and ability inhibit kinase activity. Two mutants were prepared: first, substitution of F-Q-R to A-Q-A in the site 1 affinity arm, referred to as A-X-A hereafter; and second, substitution of K-R-T to A-A-T of the site 3 inhibitory arm, referred to as A-A-T. Consistent with our crystal structure, either A-X-A or A-A-T mutation within Par3$_{CR3}$ markedly reduce the CR3-binding affinity for PKCι$_{KD}$-2P, but without abolishing the interaction entirely (Figures 5A and 5B). A phospho-S827$^{Par3}$ peptide representing the PKCι reaction product bound poorly, with affinity two orders of magnitude lower than in Par3$_{CR3}$ (Figures 5A and 5B).

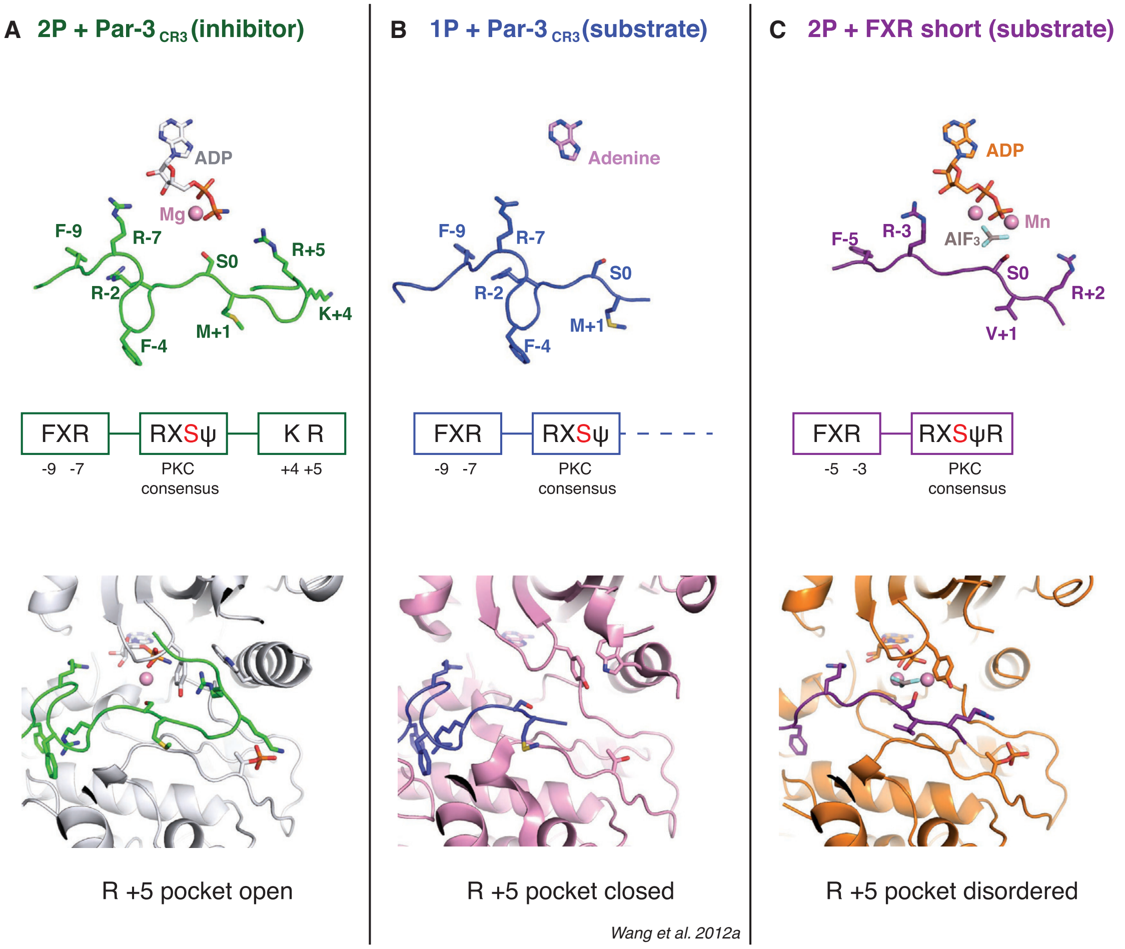


**Figure 4. Structural Comparison of aPKC-Substrate and aPKC-Inhibitor Interactions**

(A) Top panel: cartoon depiction of the $Par3_{CR3}$ (green) bound to $PKC\iota_{KD}$-2P (omitted for clarity) highlighting the position of inhibitory −7 and +5 arginine residues flanking the nucleotide (gray sticks). Middle panel: schematic representation of the interaction sites of $Par3_{CR3}$ with $PKC\iota_{KD}$-2P. Bottom panel: close up of the R+5 pocket occupied by $Par3_{CR3}$ and the Y265 and W298 clamp residues of $PKC\iota_{KD}$-2P (gray). The known phospho-acceptor site at serine 0 is shown in red for all panels.

(B) Top panel: cartoon depiction of the $Par3_{CR3}$ (blue) bound to $PKC\iota_{KD}$-1P K283R mutant (omitted for clarity) from Wang et al. (2012a) lacking inhibitory site 3 contacts, possibly reflecting a lower-affinity substrate-type interaction. Middle panel: schematic representation of the interaction sites of $Par3_{CR3}$ with $PKC\iota_{KD}$-1P K283R mutant. Bottom panel: close up of the "closed" R+5 pocket in which the clamp residues Y265 and W298 make direct contact ($PKC\iota_{KD}$-1P shown in pink).

(C) Top panel: cartoon depiction of the $FXR_{short}$ (purple) bound to $PKC\iota_{KD}$-2P, Mn-ADP, and $AlF_3$. This artificial substrate lacks inhibitory site 3 contacts but shares site 1 FXR motif. Middle panel: schematic representation of the interaction sites of $FXR_{short}$ with $PKC\iota_{KD}$-2P. Bottom panel: close up of the ordered portion of the R+5 pocket including the glycine loop and Y265 but not the disordered W298 from the $\alpha$C helix ($PKC\iota_{KD}$-1P shown in orange).

See also Figures S4A–S4D for the design and characterization of $FXR_{short}$ peptide. See Figures S4E and S4F for refined $FXR_{short}$ peptide electron density and a superposition of the $Par3_{CR3}$ and $FXR_{short}$ peptides bound to $PKC\iota_{KD}$-2P.

Surprisingly, the in vitro kinase assay demonstrated that either an A-X-A or A-A-T mutation gave a substantial increase in $Par3_{CR3}$ phosphorylation by $PKC\iota_{KD}$-2P, greatly enhancing the apparent $k_{cat}$ values (Figures 5A and 5C). The large effects observed for each mutant (57-fold for A-X-A $Par3_{CR3}$ versus 18-fold A-A-T $Par3_{CR3}$) suggest that these substitutions uncouple the ability of $Par3_{CR3}$ to inhibit $PKC\iota_{KD}$-2P, resulting in access to the PKC consensus site at $S827^{Par3}$ and efficient phosphorylation by $PKC\iota_{KD}$-2P. The magnitude of the increased $k_{cat}$ values allowed the measurement of a $K_M$ for the A-X-A $Par3_{CR3}$ substrate ($K_M$ of 39 $\mu$M), which was not possible for wild-type $Par3_{CR3}$. Combining both site 1 and site 3 mutations (A-X-A + A-A-T) within $Par3_{CR3}$ generated a very poor substrate that was not detectably phosphorylated and had no measurable interaction with $PKC\iota_{KD}$-2P (data not shown). These data indicate that while mutating either the anchoring arm or inhibitory arm switches $Par3_{CR3}$ to an efficient aPKC substrate, the remaining arm must contribute sufficient binding affinity (both are basophilic) as mutating both arms generates a $Par3_{CR3}$ that is neither a substrate nor an inhibitor. These striking results are also consistent with the notion that tight inhibitory binding of $Par3_{CR3}$ to $PKC\iota_{KD}$-2P must prevent its phosphorylation while weaker binding without the inhibitory interactions exposes its PKC site, switching it to a highly efficient in vitro PKCι substrate.

A

| | $k_{cat}^{app}$ ($^{-1}$ min) | $K_m^{app}$ (µM) | $K_d$ (µM) |
|---|---|---|---|
| Par-$3_{CR3}$WT | 1.9±0.3 | 40.5±19 | 0.73±0.18 |
| Par-$3_{CR3}$AXA | 108±5.4 | 39.0±6.1 | 228±52 |
| Par-$3_{CR3}$AAT | 34±3.6 | 50.3±15 | 424±68 |
| Par-$3_{CR3}$S-A | 1.0±0.1 | 10.5±5.0 | 0.69±0.5 |
| Par-$3_{CR3}$pS827 | - | - | 114.6±11 |
| Par-$3_{CR3}$AXA-AAT | n.d. | n.d. | n.d. |

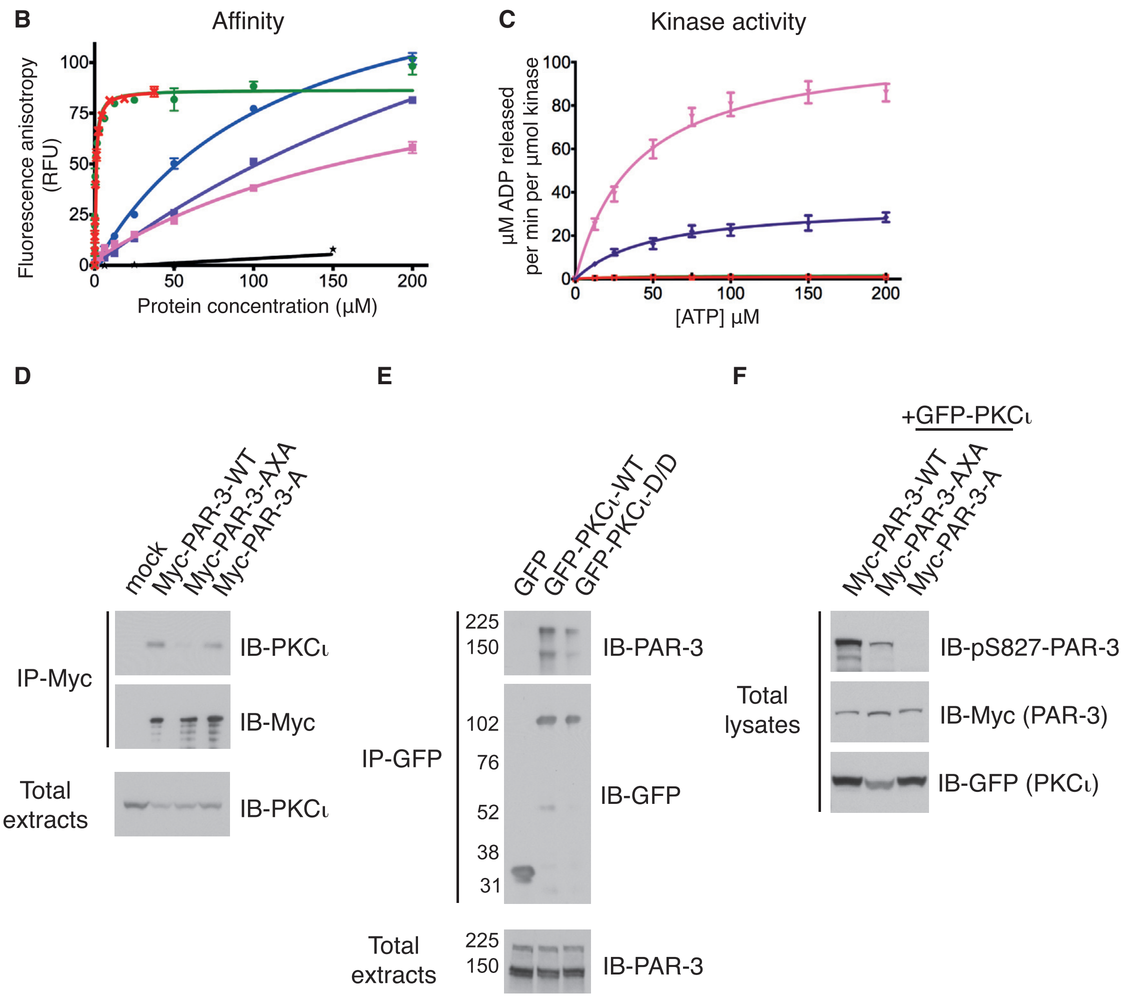


**Figure 5. Reducing the Par$3_{CR3}$ Affinity for PKC$\iota_{KD}$-2P Promotes Efficient CR3 Phosphorylation In Vitro**

(A) Summary table of $k_{cat}$, $K_D$, and $K_M$ constants between various Par$3_{CR3}$ mutants and PKC$\iota_{KD}$-2P. Data are presented as mean ± SEM. n.d., not determined.

(B) Binding curves for Par$3_{CR3}$ and various Par$3_{CR3}$ mutants determined by fluorescence polarization (color coded as in A).

(C) PKC$\iota_{KD}$-2P catalytic activity kinetic rate constants for Par$3_{CR3}$ and various Par$3_{CR3}$ mutants (color coded as in A). For further details of other inhibitory peptides similar to Par$3_{CR3}$, see Figure S5.

(D) Co-immunoprecipitation (IP) of full-length Myc-Par3 or mutants (Par3-A-X-A and Par3-A (S827A)) and GFP-PKCι from HCT-116 cells shows that the F-X-R to A-X-A mutation dramatically reduces the interaction.

(E) Co-immunoprecipitation of GFP-PKCι or GFP-PKCι-D/D with Myc-Par3 also severely impairs the interaction. GFP-PKCι-D/D is a mutant replacing residues D330/D373 that interact with the F-X-R motif by alanine.

(F) Immunoblot (IB) using a phospho-S827-specific antibody indicates that Par3 and Par3 A-X-A mutant (but not Par3-A) are phosphorylated in HCT-116 cells. For details showing evidence of phosphorylation of A-X-A Baz mutant, see Figure S6.

To validate some aspects of these results, using full-length PKCι and Par3 in cells we undertook co-immunoprecipitation experiments of differentially tagged full-length forms of PKCι and Par3 expressed in transiently transfected HCT-116 cells. Endogenous PKCι was efficiently immunoprecipitated through exogenous wild-type Par3, while a full-length human Par3 bearing the site 1 A-X-A mutation showed substantially reduced interaction with PKCι (Figure 5D), consistent with in vitro data for the isolated CR3 domain. Note that endogenous PKCι retains binding to the non-phosphorylatable Par3-S827A (Myc-PAR-3-A) similarly to the wild-type but is unable to be turned over and remains tightly associated with PKCι. Reciprocal co-immunoprecipitation of overexpressed exogenous wild-type GFP-PKCι efficiently pulled down endogenous Par3, whereas mutating residues $D339^{PKC\iota}/D382^{PKC\iota}$ (GFP-PKCι-D/D) that directly contact the R−7 side chain of Par3 also markedly reduced the Par3-PKCι interaction (Figure 5E). Arm contacts identified from the crystal structure are therefore necessary for Par3 interaction with PKCι. We developed a specific phospho-Par3 antibody to probe whether mutation of the A-X-A arm abolished interaction with PKCι completely as well as PKCι-mediated phosphorylation. While the A-X-A is less phosphorylated compared with wild-type (Figure 5F), we noted a large increase in ubiquitinylated Par3 (under conditions of proteasome inhibition), a likely consequence of Par3 phosphorylation in non-polarized cells (data not shown). Taken together, immunocomplex recovery from HCT-116 cells confirmed that (1) the contacts observed structurally indeed influence interaction in cells as predicted, and (2) "weakening" the strength of the aPKCι-Par3 interaction through site-specific mutation prevents Par3 inhibition, leading instead to Par3 phosphorylation.

## Apical-Junctional Polarization of Par3/Baz In Vivo Is a Consequence of Switching between Inhibitory and Substrate-Binding Modes

If the affinity of the Par3/Baz-aPKC interaction essentially determines the localization of Par3/Baz in epithelial cells, then the $Par3_{CR3}$ substitutions within each arm (A-X-A or A-A-T mutants) characterized in vitro should affect the localization of Par3/Baz in vivo. To test this prediction, we mutated the CR3 region of full-length GFP-tagged *Drosophila* Baz in the F-X-R motif to A-X-A or the K-H-T motif to A-A-T and examined their apical domain or AJ localization in vivo. In the follicular epithelium, GFP-tagged wild-type Baz (GFP-Baz) co-localizes with aPKC at the apical membrane and also localizes to AJs (Figure 6A). Phospho-Baz is known to localize to AJs (Morais-de-Sa et al., 2010), and a GFP-tagged phosphomimic version of Baz (GFP-Baz S980E) expectedly fails to co-localize with aPKC at the apical membrane but instead localizes to AJs (Figure 6B) (Morais-de-Sa et al., 2010; Walther and Pichaud, 2010). Both the GFP-Baz A-X-A and A-A-T mutant localize similarly to the phospho-mimetic (Figures 6C, 6D, and 6I), consistent with the view that lowering affinity (as observed in cells; Figure 5B) and/or removing inhibitory elements from CR3 induces phosphorylation of Baz (as observed in vitro; Figure 5) and therefore results in its localization to AJs rather than stable Par complex formation at the apical membrane.

It was important to distinguish between whether the relocalization of the Baz A-X-A or A-A-T mutants was due to exposure of the S980 site and phosphorylation as shown in vitro for $Par3_{CR3}$, or simply due to a lack of interaction with aPKC. A combined A-X-A + A-A-T site mutation in vitro showed a complete loss of interaction of CR3 with PKCι and no phosphorylation of serine 827 (Figure 5A). An equivalent GFP-Baz A-X-A + A-A-T mutant also localized to AJs (Figures 6H, 6H′, and 6M). Interestingly, this combined mutant showed distinct intracellular puncta in which the Baz-GFP mutant no longer overlapped with aPKC, suggesting that both proteins are mutually exclusive on the same membrane (Figure 6N).

We then sought to verify whether the A-X-A Baz mutant was indeed phosphorylated in *Drosophila* cells. Available phospho-antibodies against Par3 S827 and Baz S980 were previously raised against an epitope that included the F-X-R motif and therefore could not detect the Baz A-X-A mutant or its phosphorylation status (data not shown). Efforts to raise a Baz phospho-antibody against S980 peptides excluding the F-X-R motif were not successful. Therefore, we verified that the A-X-A Baz mutant was phosphorylated in *Drosophila* cells, by preparing transfected S2 cell extracts containing wild-type or A-X-A mutant Baz and probed $S980^{Baz}$ phosphorylation status using dimethyl labeling and mass spectrometry. Previous efforts to identify the $Baz_{CR3}$ phospho-site in wild-type and A-X-A mutant contexts using trypsin digest were unsuccessful due to cleavage at R979 and K984, yielding very short peptides. Therefore, $Baz_{CR3}$ samples were first treated by in-gel reductive dimethylation, to generate the $Baz_{CR3}$ peptide spanning the sequence (phospho)SISE(me2K)HHAALDAR. The dimethylation reaction modifies lysine ε-amino groups, thereby greatly reducing the ability of trypsin to cleave after lysines. This allowed capture of the phospho-$Baz_{CR3}$ peptide, facilitated quantification of chromatographic peak areas, and identified phospho-peptides. The forward sample reaction used heavy ($CD_2O$ with wild-type Baz mutant) or light ($CH_2O$ with A-X-A Baz) reagents, resulting in a mass difference of 12 Da and an *m/z* difference of 4 for the triply charged target peptide (Figures S6A and S6B). The reverse sample used heavy ($CD_2O$ with A-X-A Baz mutant) or light ($CH_2O$ with wild-type Baz) reagents, and two control peptides were also used to assess any differences in peptide recovery from the SDS-PAGE gel (Figures S6A and S6C). Recovery was poorer for all heavy-labeled reverse samples including both controls, although the data clearly showed that both wild-type and A-X-A Baz proteins were phosphorylated at $S980^{Baz}$ (Figures S6C and S6D). Taken together, our data suggest that the A-X-A mutant can be phosphorylated by aPKC in vitro in HCT-116 and S2 cells. Moreover, it can be distinguished from the A-X-A + A-A-T combined mutant that is no longer a substrate for aPKC and fails to interact with it both in vitro and in vivo.

We next tested the idea that phosphorylation of Par3/Baz controls its localization simply by feeding back to block its binding to aPKC (as observed for phospho-$Par3_{CR3}$ in vitro; Figure 5). A GFP-tagged phospho-mutant form of Baz (GFP-Baz S980A) is known to fail to localize to junctions and instead co-localizes perfectly with aPKC (Figures 6E, 6J, and S7) (Morais-de-Sa et al., 2010; Walther and Pichaud, 2010). We find that expression of this construct also disrupts cellular morphology, consistent with previously reported data (Morais-de-Sa et al., 2010). If the localization and morphology phenotypes of GFP-Baz S980A are caused by tight inhibitory binding to aPKC, it should be

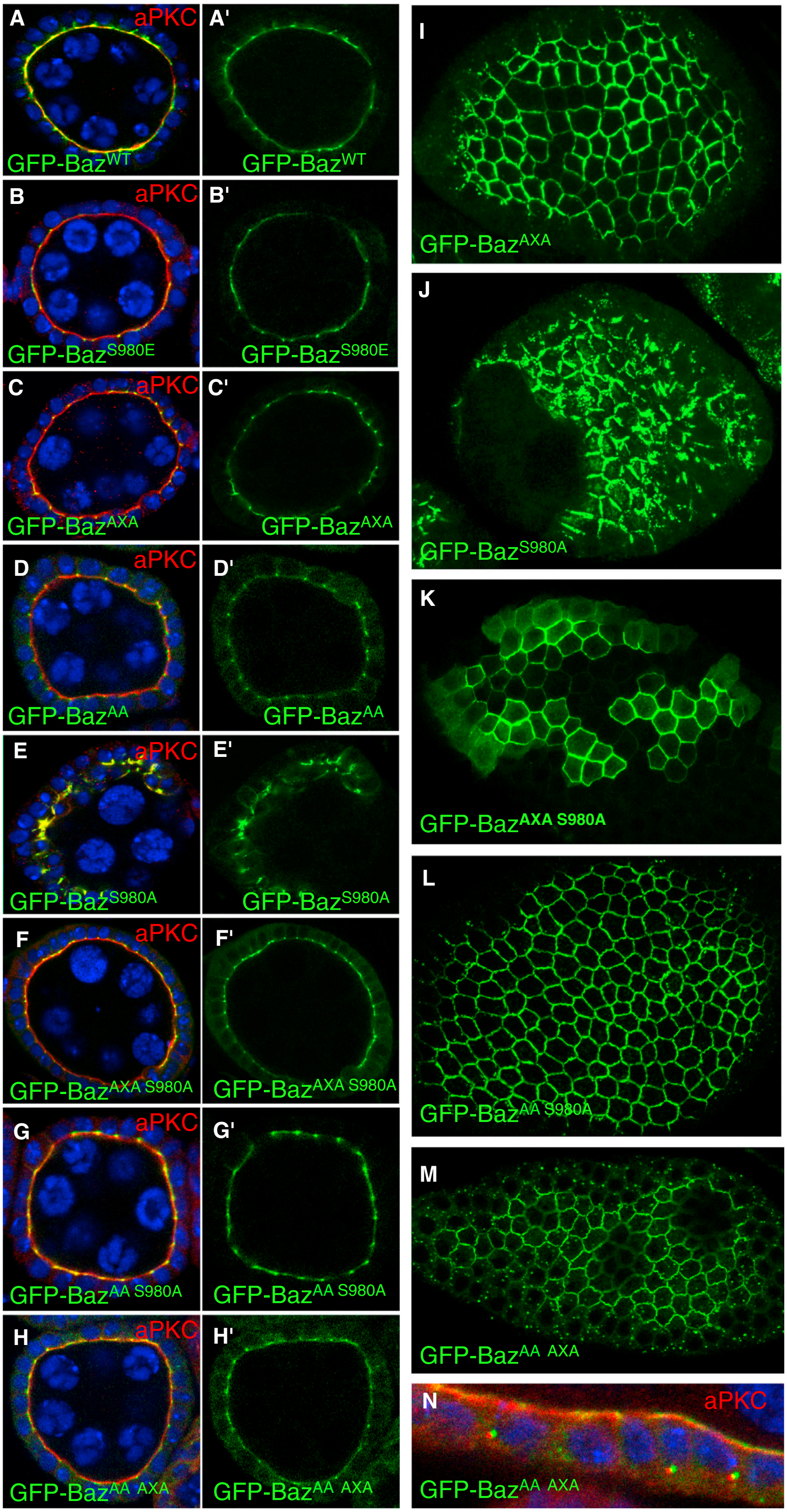


**Figure 6. Switching Par3/Baz from Apical to Junctional In Vivo**

(A) GFP-tagged Par3/Baz (green) localizes to the apical domain (marked by aPKC in red) and also to AJs.

(B) Phosphomimic GFP-tagged Par3/Baz S980E (green) is largely excluded from the apical domain (marked by aPKC in red) and localizes to AJs.

(C) Low-affinity GFP-tagged Par3/Baz F-X-R to A-X-A mutant (green) is largely excluded from the apical domain (marked by aPKC in red) and localizes to AJs.

(D) Low-affinity GFP-tagged Par3/Baz K-H to A-A mutant (green) is largely excluded from the apical domain (marked by aPKC in red) and localizes to AJs.

(E) Phospho-mutant GFP-tagged Par3/Baz S980A mutant (green) co-localizes apically with aPKC (red) and also partially disrupts cell polarity, consistent with its inhibitory function. See also Figure S7 for evidence that Baz co-localizes with aPKC in the absence of kinase activity.

(F) Phospho-mutant GFP-tagged Par3/Baz S980A that also carries the F-X-R to A-X-A mutation (green) fails to co-localize with aPKC (red) and instead localizes to AJs.

(G) Phospho-mutant GFP-tagged Par3/Baz S980A that also carries the K-H to A-A mutation (green) fails to co-localize with aPKC (red) and instead localizes to AJs.

(H) The double mutant K-H to A-A and F-X-R to A-X-A localizes primarily to AJs.

(I) Apical section of GFP-BazAXA expressing follicle cell epithelium, showing junctional localization.

(J–M) Apical section of GFP-BazS980A (J) expressing follicle cell epithelium, showing mislocalization to the apical surface. Apical sections of (K) GFP-BazAXA S980A-, (L) GFP-BazAA S980A-, and (M) GFP-BazAA AXA-expressing follicle cell epithelium, showing restoration of junctional localization.

(N) Non-overlapping punctate localization of GFP-BazAXA AA (green) with aPKC (red).

DAPI staining is shown in blue in (A)–(H) and (N). GFP-tagged Par3/Baz is shown in (A′)–(H′).

CellPress

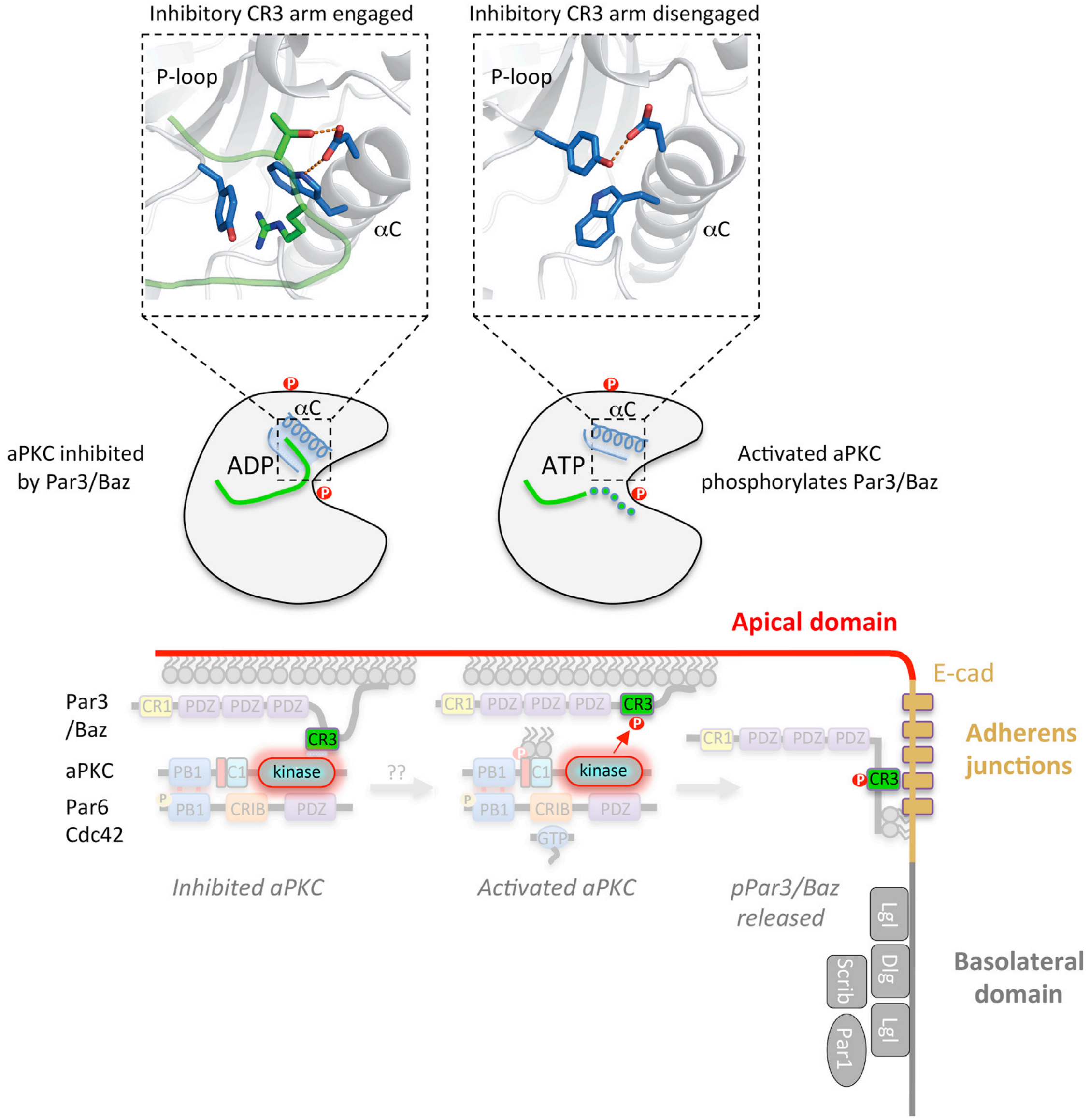


**Figure 7. Proposed Model for Par3/Baz Antagonism and Polarization**

We propose two states for PKC-Par3/Baz interaction driven by aPKC kinase domain and Par3/Baz CR3 region. A high-affinity interaction is inhibitory and requires both arms flanking the PKC consensus motif of the CR3 region. By engaging pockets within both the N and C lobes of aPKC kinase domain, Par3/Baz phosphorylation is prevented but Par6-aPKC is recruited to the apical membrane. In an activated state, aPKC is resistant to CR3 antagonism due either to an inaccessible αC helix pocket or a CR3 interaction being destabilized by phosphorylation of T833$^{Par3}$ or by aPKC lacking a PDK1-driven T412$^{PKC\iota}$ phosphorylation. Either of these possibilities could result in Par3/Baz binding as a substrate exposing its PKC consensus site R-X-S-Ψ to phospho-transfer. Phosphorylated Par3/Baz is then excluded from the Par complex and thus from the apical membrane domain, and relocalizes to AJs.

possible to reverse these phenotypes by introducing either the F-X-R or K-H-T site mutation to lower the affinity of this interaction. Accordingly, we find that GFP-Baz A-X-A or A-A-T S980A double mutants fail to co-localize with aPKC and instead localize to AJs and do not show polarity defects (Figures 6F–6L). These results strongly support the view that phosphorylation of Par3/Baz controls its localization through lowering its binding affinity for aPKC, because the phenotypic consequence of loss of phosphorylation can be reversed by mutations that reduce affinity. Consistent with our in vitro data, we propose that access to the phosphorylation site within Par3/Baz is in turn controlled by modulation of the high-affinity and inhibitory arms of the CR3 region.

## DISCUSSION

Our results reveal the molecular basis for Par3 antagonism of aPKC through high-affinity inhibitory CR3 arm interactions that span both N and C lobes of the PKCι kinase domain. Our structural and biochemical data provide a model that supports a mechanism explaining apical-junctional polarization of Par3/Baz in epithelial cells (Figure 7). Previous work has shown that

apical localization of Par3/Baz depends on it being part of the Par complex, where Par3/Baz is not phosphorylated by aPKC, while junctional localization of Par3/Baz occurs when it is phosphorylated by aPKC (Morais-de-Sa et al., 2010; Walther and Pichaud, 2010). Formation of the Par complex with Par3/Baz is known to be crucial for apical membrane recruitment of aPKC-Par6 (Gao et al., 2002).

Here we provide an explanation for why Par3/Baz is not phosphorylated while engaged within the Par complex even though it can be phosphorylated when it separates from the Par complex. Our crystal structure of the Par3 CR3–PKCι kinase domain interaction reveals the basis for high-affinity $Par3_{CR3}$ contacts through the coordinated action of two short motifs flanking the PKC consensus motif R-X-S827-$\Psi$. Together these motifs cooperate to inhibit aPKC, one bringing high affinity and the other enabling inhibitory contacts. The observation that the same N-lobe pocket is closed in structures of partially primed $PKC\iota_{KD}$-1P with a peptide resembling an aPKC-substrate interaction supports a second mode of engagement of Par3. Access to this N-lobe pocket may dictate whether aPKC-interacting proteins with an R+5 hook can inhibit aPKC or are phosphorylated as substrates. The location of the pocket adjacent to the αC helix and the aPKC activation loop suggests a potential mechanism to regulate the decision to engage and phosphorylate or to be sensitized to Par3 CR3 inhibition in the case of a fully primed active aPKC conformer.

The precise mechanism determining this switch requires further study and is beyond the scope of these investigations. Potential regulatory influences could include the availability of Par3 inhibitory arms, competition with other aPKC substrates, the presence of binding partners adjacent to the aPKC αC helix, post-translational modifications of the $Par3_{CR3}$ region (such as $T833^{Par3}$ phosphorylation by the ROCK kinase), or even regulation of the aPKC activation loop (by PDK1 or by dephosphorylation by an unknown A-loop phosphatase). One or more of these factors, depending on the physiopathological status of the epithelium, could affect the Par3-binding mode. We do note that Kibra (and its homolog WWC2) contains an F-X-R motif and a K-R-T hook flanking its known aPKC phosphorylation site (between residues 911 and 978), suggesting that it too could act as dual-action inhibitor/substrate, consistent with biochemical data (Figure S5) (Yoshihama et al., 2011). Similarly, many known aPKC substrates have an adjacent F-X-R motif, for example, Par1, Par2, and ROCK kinase, suggesting that the F-X-R may provide specificity and an affinity boost to these validated aPKC substrates (Hurov et al., 2004; Ishiuchi and Takeichi, 2011; Motegi et al., 2011; Suzuki et al., 2004).

Our evidence indicates that engineered lower-affinity interactions between the Par3/Baz CR3 domain and the aPKC kinase domain result in Par3/Baz CR3 phosphorylation (Figures 5A–5C). Mutation of either the F-X-R or K-H-T site that our structure shows are important for a high-affinity inhibitory interaction leads to a relocalization of Par3/Baz away from the apical domain (where the Par complex resides) to AJs, similar to a phosphomimetic S980E mutant in Par3/Baz (Figures 6A–6D). Combining both mutations further lowers the affinity, leading to a form of Par3/Baz unable to engage aPKC that cannot be phosphorylated by it. Such a mutant Par3/Baz also relocalizes to AJs. Thus, Par3/Baz that fails to form a stable inhibitory Par complex will localize to AJs either through aPKC-mediated phosphorylation or through a loss of interaction.

Why does phosphorylation of Par3/Baz cause its localization to AJs? Our findings show that phospho-Par3/Baz dramatically reduces its affinity for aPKC and thus the phosphorylation event precludes it from joining the Par complex. Phosphomimic S980E Par3/Baz is known to localize to AJs, just like the affinity-lowering A-X-A or A-A-T mutants of Par3/Baz (Figure 6). Furthermore, the behavior of phospho-resistant mutant S980A Par3/Baz (which only localizes with aPKC) can be reversed in A-X-A S980A or A-A-T S980A double mutant Par3/Baz (which only localizes to AJs) (Figure 6). Previous studies have proposed that the AJ localization of Par3/Baz results from exclusion from the apical domain upon aPKC phosphorylation combined with exclusion from the basolateral domain upon Par1 phosphorylation (Tepass, 2012). Taken together, our data stimulate a model in which aPKC-driven phosphorylation of Par3/Baz can be recapitulated simply by weakening the Par3/Baz interaction affinity by manipulating the sequences flanking the consensus PKC phosphorylation site (Figure 7).

Our findings implicate both Par3/Baz and Kibra as aPKC inhibitors that are also known substrates. An analogous situation arises for LGL, a known inhibitor of aPKC that is also phosphorylated at multiple serine sites (Bailey and Prehoda, 2015). There are also precedents for protein kinase dual-action inhibitor/substrates. The protein kinase A (PKA) regulatory subunit RIIβ has an RRXS motif that is phosphorylated by PKA, leading to its stable association with and inhibition of PKA (Zhang et al., 2015). In this context the modification functions as part of a single-turnover phosphoryl transfer reaction. For Par3 and other F-X-R-containing proteins, a different role is likely whereby phosphorylation by aPKC provokes Par3/Baz dissociation, as shown in vitro using CR3 peptides and in vivo using phospho-mimetics. Another example is the cyclin-dependent kinase inhibitors p21/p27/KIP, which are able to both inhibit cyclin-dependent kinases as well as being efficient substrates (Russo et al., 1996).

Our findings suggest that aPKC is inhibited by Par3/Baz within the Par complex, yet it is known that the Par complex contains active aPKC kinase and can phosphorylate many substrates (such as Lgl and Par1 in *Drosophila* epithelia and *Caenorhabditis elegans* zygotes, and Miranda in *Drosophila* neuroblasts). One possible explanation for this open issue is that discrete functional states of the Par complex (Par6-aPKC-Cdc42-Par3) may exist. Par3-dependent recruitment of aPKC to apical membranes may evoke a higher-order oligomer consistent with known Par3 CR1-dependent oligomers (Benton and St Johnston, 2003a). Conversely, phosphorylation of $T833^{Par3}$ by ROCK kinase or a lack of $T412^{PKC\iota}$ phosphorylation by PDK1 would generate functionally distinct forms of the Par complex, unable to be inhibited by Par3 CR3 as discussed earlier. Equally, association of partner proteins close to the aPKC αC helix could also block the formation of an R+5 pocket and prevent CR3-mediated inhibition. Therefore, further experiments are required to characterize precisely which polarity signal(s) provoke Par3 phosphorylation by overcoming CR3 antagonism.

In conclusion, our findings provide a molecular basis for Par3-mediated antagonism of aPKC that affects apical versus junctional polarization of Par3/Baz in epithelia.

## EXPERIMENTAL PROCEDURES

### Protein Construct Design, Expression, and Purification

Mammalian plasmids pEGFP-PKCι-WT, pEGFP-PKCι-DD/AA (D339A/D382A), pK-myc-Par3-WT (Addgene, plasmid 19388), pK-myc-Par3-AXA (F818A/R820A), and pK-myc-Par3-A (S827A) included human PKCι and Par3 cDNAs. Mutagenesis of PKCι and Par3 was performed using QuikChange (Stratagene). Recombinant human PKC-iota kinase domain (PKC$\iota_{KD}$) was prepared using a baculovirus encoding residues 248–596 (GenBank: NM_002740.5), fused to a glutathione S-transferase (GST) tag as described previously (Kjaer et al., 2013). See Supplemental Experimental Procedures for a detailed description. In brief, the protein was expressed in Hi5 cells by co-infection with the above virus and a PDK-1 virus using standard protocols (Oxford Expression Technology). The GST tag was used for affinity purification and removed by 3C protease cleavage using standard protocols. Two distinct phospho-species PKC$\iota_{KD}$-2P and PKC$\iota_{KD}$-1P were separated by ion-exchange chromatography (Hi-Trap Q column, GE Healthcare).

### Enzymatic Assay and Fluorescence Anisotropy Binding Assay

The ADP Quest kit (DiscoveRx) was used to determine the $k_{cat\ app}$ and $K_{M\ app}$ values for ATP against the PKC$\iota_{KD}$-1P and PKC$\iota_{KD}$-2P using a series of synthetic peptide substrates as described by Kjaer et al. (2013). The reactions were measured every 2 min for 30 min in a 384-well plate using a Safire$^2$ plate reader (Tecan). The kinetic constants were determined by fitting the data to the Michaelis-Menten equation. Data are represented as mean ± SEM. Fluorescence anisotropy assays were performed to determine the $K_D$ for each peptide labeled with a fluorescein tag following standard protocols using a Safire$^2$ plate reader (Tecan). The anisotropy values were normalized and the $K_D$ was determined using non-linear regression. All experiments were performed in triplicate and for at least three independent protein preparations.

### Structure Determination of Nucleotide-Bound PKC$\iota_{KD}$-2P Complexes

PKC$\iota_{KD}$-2P was incubated with a 3-molar excess of nucleotide or analog with either $Mg^{2+}$ or $Mn^{2+}$ (see Table 1) and a 3-molar excess of peptide. Crystallization was performed using the hanging-drops method with 1:1 ratio of protein to precipitant at 20°C. X-ray data were collected at synchrotrons as specified by Table 1 and data were processed using either XDS (Kabsch, 2010b) and Xscale (Kabsch, 2010a) or D*Trek (Pflugrath, 1999) and Scala/Pointless (Collaborative Computational Project-Number 4, 1994). Structures were determined by molecular replacement performed using Phaser (McCoy et al., 2007) using a previous PKC ι-2P structure as a search model (PDB: 3A8W). Refinement was carried out in Phenix (Adams et al., 2010) with cycles of model building in Coot (Emsley and Cowtan, 2004).

### Cell Culture and Transfection

HCT-116 cells grown in McCoy's 5A medium containing 10% bovine fetal calf serum and penicillin/streptomycin (Invitrogen) were transfected (10-μg portion of DNA or 5-μg + 5-μg portions of DNA for co-transfections) using Fugene HD (Roche) according to the manufacturer's instructions. The cells were then grown in normal medium for 36 hr.

### Antibodies, Immunoprecipitation, and Immunoblotting

The following antibodies were used for immunoblotting: mouse monoclonal anti-PKCι (recognition for human PKCι), mouse monoclonal anti-Myc (9 × $10^{10}$), rabbit polyclonal anti-Par3 (Millipore), and rabbit polyclonal anti-GFP antibody (Santa Cruz Biotechnology). Anti-phospho-S827 Par3 antibody was raised in-house using an antigen lacking the F-X-R site of Par3$_{CR3}$. Immunoprecipitation and immunoblotting was carried out as described in Supplemental Experimental Procedures.

### Dimethyl Labeling and Quantification of Bazooka Wild-Type and Mutant S980 Phosphorylation in S2 Cell Extracts

After SDS-PAGE, in-gel stable isotope dimethyl labeling was performed according to published protocols. The heavy reaction was performed using $^{13}CD_2O$ formaldehyde creating a mass difference of 6 Da per primary amine group between heavy and light dimethylated peptides. After extensive washing of gel pieces, the in-gel dimethylated proteins were then subjected to overnight in-gel trypsin digestion at 37°C. The following day peptides were extracted and subjected to another round of reductive dimethylation reactions aimed at methylating peptide N termini. Peptide mixtures were acidified and prepared for liquid chromatography-mass spectrometry analysis using an Ulimate3000 high-performance liquid chromatograph coupled to a Q-Exactive mass spectrometer (Thermo Fisher). A targeted scan was performed for the S980-containing peptides and this was alternated with a top-10 data-dependent acquisition scan. Mascot-generated DAT files were converted to Skyline-compatible biblio.spec libraries, and heavy and light peak areas were extracted by Skyline software version 2.5.0.6079 (MacLean et al., 2010).

### *Drosophila* Genetics and Oligonucleotides

Expression of *UAS*-driven transgenes in follicle cells was achieved with the GR1.Gal4 line. *UAS.GFP-Baz* lines were constructed by mutagenizing the full-length Baz cDNA in pDONR, followed by transfer to the pPGW (pUASP-EGFP-Gateway) vector for transgenesis (Bestgene). The *UAS.GFP-BazS980E* line was a gift from F. Pichaud. Primers used for mutagenesis are described in Supplemental Experimental Procedures.

### *Drosophila* Antibodies and Immunohistochemistry

Ovaries were dissected in PBS, fixed for 20 min in 4% paraformaldehyde in PBS, washed for 30 min in PBS/0.1% Triton X-100 (PBST), and blocked for 15 min in 5% normal goat serum/PBST (PBST/NGS). Primary antibodies were diluted in PBST/NGS and samples were incubated overnight at 4°C. Either optical cross-sections through the middle of egg chambers or apical sections of the follicular epithelium are shown. Primary antibodies used are described in Supplemental Experimental Procedures.

## ACCESSION NUMBERS

Coordinates and structure factors for PKC$\iota_{KD}$-2P-Par3$_{CR3}$, PKC$\iota_{KD}$-2P-FXR$_{short}$ peptide, and nucleotide-bound PKC$\iota_{KD}$-2P have been deposited in the PDB with the accession numbers PDB: 5LI1, 5LIH, and 5LI9, respectively.

## SUPPLEMENTAL INFORMATION

Supplemental Information includes Supplemental Experimental Procedures and seven figures and can be found with this article online at http://dx.doi.org/10.1016/j.devcel.2016.07.018.

## AUTHOR CONTRIBUTIONS

E.V.S. performed all stages of the Mg-AMPPNP-Par3$_{CR3}$-PKC$\iota_{KD}$-2P peptide structure determination and refinement. A.P. refined the Mn-ADP-PKC$\iota_{KD}$-FXR$_{short}$ peptide structure. M.I. purified, crystallized, and refined the AMPPCP-complexed PKC$\iota_{KD}$-2P structure. E.V.S. and M.I. carried out kinetic assays and fluorescence anisotropy measurements. P.K. and S.K. assisted with protein production and virus preparation. A.E. prepared S2 cell extracts containing wild-type and mutant Baz protein. K.B. and B.S. performed the dimethyl-labeling and mass spectrometry analyses. B.K. prepared and characterized recombinant PKC$\iota_{KD}$ and PKC$\zeta_{KD}$ proteins. P.S. prepared constructs for full-length human Par3 and mutants. N.O'R. purified all peptides used in this study. P.R. conducted co-immunoprecipitations using full-length Par3 with full-length PKCι and raised the anti-phospho-Par3 antibody. M.L. prepared the PKCι D/D mutant. G.F. and B.J.T. carried out all the *Drosophila* experiments. M.I. prepared the figures. N.Q.M. and B.J.T. planned the project, designed experiments, and wrote the paper.

## ACKNOWLEDGMENTS

We thank members of the McDonald and Parker laboratories for helpful discussions and comments on the manuscript. We thank Nic Tapon for help with purifying Bazooka from S2 cells. This work was supported by the Francis Crick Institute which receives its core funding from Cancer Research UK (FC001115, FC001130, FC001180), the UK Medical Research Council (FC001115, FC001130, FC001180), and the Wellcome Trust (FC001115,

FC001130, FC001180). M.L. is supported by the National Institute for Health Research and UCL Hospitals Biomedical Research Center.

Received: April 30, 2013
Revised: May 24, 2016
Accepted: July 24, 2016
Published: August 22, 2016

## REFERENCES

Adams, J.A., and Taylor, S.S. (1993). Divalent metal ions influence catalysis and active-site accessibility in the cAMP-dependent protein kinase. Protein Sci. *2*, 2177–2186.

Adams, P.D., Afonine, P.V., Bunkoczi, G., Chen, V.B., Davis, I.W., Echols, N., Headd, J.J., Hung, L.W., Kapral, G.J., Grosse-Kunstleve, R.W., et al. (2010). PHENIX: a comprehensive Python-based system for macromolecular structure solution. Acta Crystallogr. D Biol. Crystallogr. *66*, 213–221.

Bailey, M.J., and Prehoda, K.E. (2015). Establishment of par-polarized cortical domains via phosphoregulated membrane motifs. Dev. Cell *35*, 199–210.

Benton, R., and St Johnston, D. (2003a). A conserved oligomerization domain in *Drosophila* Bazooka/PAR-3 is important for apical localization and epithelial polarity. Curr. Biol. *13*, 1330–1334.

Benton, R., and St Johnston, D. (2003b). *Drosophila* PAR-1 and 14-3-3 inhibit Bazooka/PAR-3 to establish complementary cortical domains in polarized cells. Cell *115*, 691–704.

Betschinger, J., Eisenhaber, F., and Knoblich, J.A. (2005). Phosphorylation-induced autoinhibition regulates the cytoskeletal protein Lethal (2) giant larvae. Curr. Biol. *15*, 276–282.

Bilder, D., Schober, M., and Perrimon, N. (2003). Integrated activity of PDZ protein complexes regulates epithelial polarity. Nat. Cell Biol. *5*, 53–58.

Buther, K., Plaas, C., Barnekow, A., and Kremerskothen, J. (2004). KIBRA is a novel substrate for protein kinase Czeta. Biochem. Biophys. Res. Commun. *317*, 703–707.

Collaborative Computational Project-Number 4 (1994). The CCP-4 suite: programs for protein crystallography. Acta Crystallogr. D Biol. Crystallogr. *50*, 760–763.

Emsley, P., and Cowtan, K. (2004). Coot: model-building tools for molecular graphics. Acta Crystallogr. D Biol. Crystallogr. *60*, 2126–2132.

Fletcher, G.C., Lucas, E.P., Brain, R., Tournier, A., and Thompson, B.J. (2012). Positive feedback and mutual antagonism combine to polarize crumbs in the *Drosophila* follicle cell epithelium. Curr. Biol. *22*, 1116–1122.

Gao, L., Joberty, G., and Macara, I.G. (2002). Assembly of epithelial tight junctions is negatively regulated by Par6. Curr. Biol. *12*, 221–225.

Grodsky, N., Li, Y., Bouzida, D., Love, R., Jensen, J., Nodes, B., Nonomiya, J., and Grant, S. (2006). Structure of the catalytic domain of human protein kinase C beta II complexed with a bisindolylmaleimide inhibitor. Biochemistry *45*, 13970–13981.

Harris, K.P., and Tepass, U. (2008). Cdc42 and Par proteins stabilize dynamic adherens junctions in the *Drosophila* neuroectoderm through regulation of apical endocytosis. J. Cell Biol. *183*, 1129–1143.

Hurov, J.B., Watkins, J.L., and Piwnica-Worms, H. (2004). Atypical PKC phosphorylates PAR-1 kinases to regulate localization and activity. Curr. Biol. *14*, 736–741.

Hutterer, A., Betschinger, J., Petronczki, M., and Knoblich, J.A. (2004). Sequential roles of Cdc42, Par-6, aPKC, and Lgl in the establishment of epithelial polarity during *Drosophila* embryogenesis. Dev. Cell *6*, 845–854.

Ishiuchi, T., and Takeichi, M. (2011). Willin and Par3 cooperatively regulate epithelial apical constriction through aPKC-mediated ROCK phosphorylation. Nat. Cell Biol. *13*, 860–866.

Izumi, Y., Hirose, T., Tamai, Y., Hirai, S., Nagashima, Y., Fujimoto, T., Tabuse, Y., Kemphues, K.J., and Ohno, S. (1998). An atypical PKC directly associates and colocalizes at the epithelial tight junction with ASIP, a mammalian homologue of *Caenorhabditis elegans* polarity protein PAR-3. J. Cell Biol. *143*, 95–106.

Joberty, G., Petersen, C., Gao, L., and Macara, I.G. (2000). The cell-polarity protein Par6 links Par3 and atypical protein kinase C to Cdc42. Nat. Cell Biol. *2*, 531–539.

Kabsch, W. (2010a). Integration, scaling, space-group assignment and post-refinement. Acta Crystallogr. D Biol. Crystallogr. *66*, 133–144.

Kabsch, W. (2010b). XDS. Acta Crystallogr. D Biol. Crystallogr. *66*, 125–132.

Kjaer, S., Linch, M., Purkiss, A., Kostelecky, B., Knowles, P.P., Rosse, C., Riou, P., Soudy, C., Kaye, S., Patel, B., et al. (2013). Adenosine-binding motif mimicry and cellular effects of a thieno[2,3-d]pyrimidine-based chemical inhibitor of atypical protein kinase C isoenzymes. Biochem. J. *451*, 329–342.

Kornev, A.P., Taylor, S.S., and Ten Eyck, L.F. (2008). A helix scaffold for the assembly of active protein kinases. Proc. Natl. Acad. Sci. USA *105*, 14377–14382.

Laprise, P., and Tepass, U. (2011). Novel insights into epithelial polarity proteins in *Drosophila*. Trends Cell Biol. *21*, 401–408.

Leonard, T.A., Rozycki, B., Saidi, L.F., Hummer, G., and Hurley, J.H. (2011). Crystal structure and allosteric activation of protein kinase C betaII. Cell *144*, 55–66.

Limatola, C., Schaap, D., Moolenaar, W.H., and van Blitterswijk, W.J. (1994). Phosphatidic acid activation of protein kinase C-zeta overexpressed in COS cells: comparison with other protein kinase C isotypes and other acidic lipids. Biochem. J. *304*, 1001–1008.

Lin, D., Edwards, A.S., Fawcett, J.P., Mbamalu, G., Scott, J.D., and Pawson, T. (2000). A mammalian PAR-3-PAR-6 complex implicated in Cdc42/Rac1 and aPKC signalling and cell polarity. Nat. Cell Biol. *2*, 540–547.

MacLean, B., Tomazela, D.M., Shulman, N., Chambers, M., Finney, G.L., Frewen, B., Kern, R., Tabb, D.L., Liebler, D.C., and MacCoss, M.J. (2010). Skyline: an open source document editor for creating and analyzing targeted proteomics experiments. Bioinformatics *26*, 966–968.

McCaffrey, L.M., and Macara, I.G. (2009). The Par3/aPKC interaction is essential for end bud remodeling and progenitor differentiation during mammary gland morphogenesis. Genes Dev. *23*, 1450–1460.

McCaffrey, L.M., Montalbano, J., Mihai, C., and Macara, I.G. (2012). Loss of the Par3 polarity protein promotes breast tumorigenesis and metastasis. Cancer Cell *22*, 601–614.

McCoy, A.J., Grosse-Kunstleve, R.W., Adams, P.D., Winn, M.D., Storoni, L.C., and Read, R.J. (2007). Phaser crystallographic software. J. Appl. Crystallogr. *40*, 658–674.

Morais-de-Sa, E., Mirouse, V., and St Johnston, D. (2010). aPKC phosphorylation of Bazooka defines the apical/lateral border in *Drosophila* epithelial cells. Cell *141*, 509–523.

Motegi, F., Zonies, S., Hao, Y., Cuenca, A.A., Griffin, E., and Seydoux, G. (2011). Microtubules induce self-organization of polarized PAR domains in *Caenorhabditis elegans* zygotes. Nat. Cell Biol. *13*, 1361–1367.

Nagai-Tamai, Y., Mizuno, K., Hirose, T., Suzuki, A., and Ohno, S. (2002). Regulated protein-protein interaction between aPKC and PAR-3 plays an essential role in the polarization of epithelial cells. Genes Cells *7*, 1161–1171.

Nakayama, M., Goto, T.M., Sugimoto, M., Nishimura, T., Shinagawa, T., Ohno, S., Amano, M., and Kaibuchi, K. (2008). Rho-kinase phosphorylates PAR-3 and disrupts PAR complex formation. Dev. Cell *14*, 205–215.

Nishikawa, K., Toker, A., Johannes, F.J., Songyang, Z., and Cantley, L.C. (1997). Determination of the specific substrate sequence motifs of protein kinase C isozymes. J. Biol. Chem. *272*, 952–960.

Parker, P.J., and Murray-Rust, J. (2004). PKC at a glance. J. Cell Sci. *117*, 131–132.

Pearce, L.R., Komander, D., and Alessi, D.R. (2010). The nuts and bolts of AGC protein kinases. Nat. Rev. Mol. Cell Biol. *11*, 9–22.

Pflugrath, J.W. (1999). The finer things in X-ray diffraction data collection. Acta Crystallogr. D Biol. Crystallogr. *55*, 1718–1725.

Roh, M.H., Fan, S., Liu, C.J., and Margolis, B. (2003). The Crumbs3-Pals1 complex participates in the establishment of polarity in mammalian epithelial cells. J. Cell Sci. *116*, 2895–2906.

Russo, A.A., Jeffrey, P.D., Patten, A.K., Massague, J., and Pavletich, N.P. (1996). Crystal structure of the p27Kip1 cyclin-dependent-kinase inhibitor bound to the cyclin A-Cdk2 complex. Nature *382*, 325–331.

St Johnston, D., and Ahringer, J. (2010). Cell polarity in eggs and epithelia: parallels and diversity. Cell *141*, 757–774.

Suzuki, A., and Ohno, S. (2006). The PAR-aPKC system: lessons in polarity. J. Cell Sci. *119*, 979–987.

Suzuki, A., Hirata, M., Kamimura, K., Maniwa, R., Yamanaka, T., Mizuno, K., Kishikawa, M., Hirose, H., Amano, Y., Izumi, N., et al. (2004). aPKC acts upstream of PAR-1b in both the establishment and maintenance of mammalian epithelial polarity. Curr. Biol. *14*, 1425–1435.

Takimura, T., Kamata, K., Fukasawa, K., Ohsawa, H., Komatani, H., Yoshizumi, T., Takahashi, I., Kotani, H., and Iwasawa, Y. (2010). Structures of the PKC-iota kinase domain in its ATP-bound and apo forms reveal defined structures of residues 533-551 in the C-terminal tail and their roles in ATP binding. Acta Crystallogr. D Biol. Crystallogr. *66*, 577–583.

Tanentzapf, G., and Tepass, U. (2003). Interactions between the crumbs, lethal giant larvae and bazooka pathways in epithelial polarization. Nat. Cell Biol. *5*, 46–52.

Tepass, U. (1996). Crumbs, a component of the apical membrane, is required for zonula adherens formation in primary epithelia of *Drosophila*. Dev. Biol. *177*, 217–225.

Tepass, U. (2012). The apical polarity protein network in *Drosophila* epithelial cells: regulation of polarity, junctions, morphogenesis, cell growth, and survival. Annu. Rev. Cell Dev Biol. *28*, 655–685.

Walther, R.F., and Pichaud, F. (2010). Crumbs/DaPKC-dependent apical exclusion of Bazooka promotes photoreceptor polarity remodeling. Curr. Biol. *20*, 1065–1074.

Wang, C., Shang, Y., Yu, J., and Zhang, M. (2012a). Substrate recognition mechanism of atypical protein kinase Cs revealed by the structure of PKCiota in complex with a substrate peptide from Par-3. Structure *20*, 791–801.

Wang, Y.C., Khan, Z., Kaschube, M., and Wieschaus, E.F. (2012b). Differential positioning of adherens junctions is associated with initiation of epithelial folding. Nature *484*, 390–393.

Wodarz, A., Ramrath, A., Grimm, A., and Knust, E. (2000). *Drosophila* atypical protein kinase C associates with Bazooka and controls polarity of epithelia and neuroblasts. J. Cell Biol. *150*, 1361–1374.

Xu, Z.B., Chaudhary, D., Olland, S., Wolfrom, S., Czerwinski, R., Malakian, K., Lin, L., Stahl, M.L., Joseph-McCarthy, D., Benander, C., et al. (2004). Catalytic domain crystal structure of protein kinase C-theta (PKCtheta). J. Biol. Chem. *279*, 50401–50409.

Yoshihama, Y., Sasaki, K., Horikoshi, Y., Suzuki, A., Ohtsuka, T., Hakuno, F., Takahashi, S., Ohno, S., and Chida, K. (2011). KIBRA suppresses apical exocytosis through inhibition of aPKC kinase activity in epithelial cells. Curr. Biol. *21*, 705–711.

Zhang, P., Knape, M.J., Ahuja, L.G., Keshwani, M.M., King, C.C., Sastri, M., Herberg, F.W., and Taylor, S.S. (2015). Single turnover autophosphorylation cycle of the PKA RIIbeta holoenzyme. PLoS Biol. *13*, e1002192.

Zheng, J., Knighton, D.R., ten Eyck, L.F., Karlsson, R., Xuong, N., Taylor, S.S., and Sowadski, J.M. (1993). Crystal structure of the catalytic subunit of cAMP-dependent protein kinase complexed with MgATP and peptide inhibitor. Biochemistry *32*, 2154–2161.

Developmental Cell, Volume *38*

## Supplemental Information

# aPKC Inhibition by Par3 CR3 Flanking Regions

# Controls Substrate Access and Underpins

# Apical-Junctional Polarization

**Erika V. Soriano, Marina E. Ivanova, Georgina Fletcher, Philippe Riou, Philip P. Knowles, Karin Barnouin, Andrew Purkiss, Brenda Kostelecky, Peter Saiu, Mark Linch, Ahmed Elbediwy, Svend Kjær, Nicola O'Reilly, Ambrosius P. Snijders, Peter J. Parker, Barry J. Thompson, and Neil Q. McDonald**

Figure S1 related to Figure 1

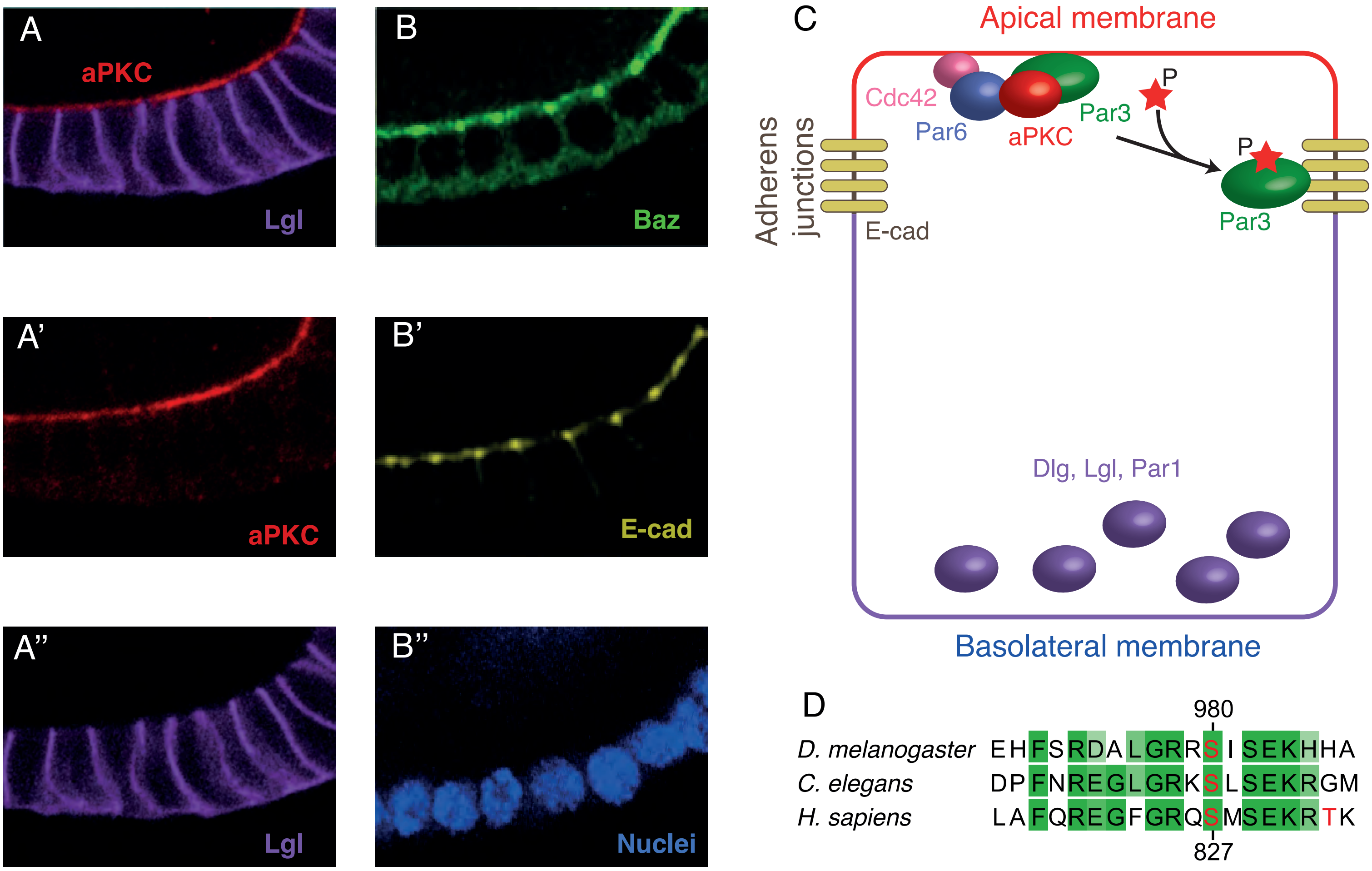


**Apical and junctional localization of Par3/Baz in Drosophila follicular epithelium.**
**(A)** and **(B)** Imaging of Drosophila follicular epithelial cells showing the localisation of various polarity determining proteins to distinct apical, junctional or basolateral locations. White arrows indicate the adherens junctions, staining positive for Baz. Grey arrows indicate the pool of apical membrane localised Baz **(C)** Model for Par3/Baz junctional polarization proposed by Morais de Sa and colleagues relating pools of Baz found at each location depending on their aPKC-site phosphorylation status (Morais-de-Sa et al., 2010). **(D)** Sequence alignment of Par3/Baz CR3 regions from three species highlighting the conserved human S827 phosphorylation site.

Figure S2 related to Figure 1

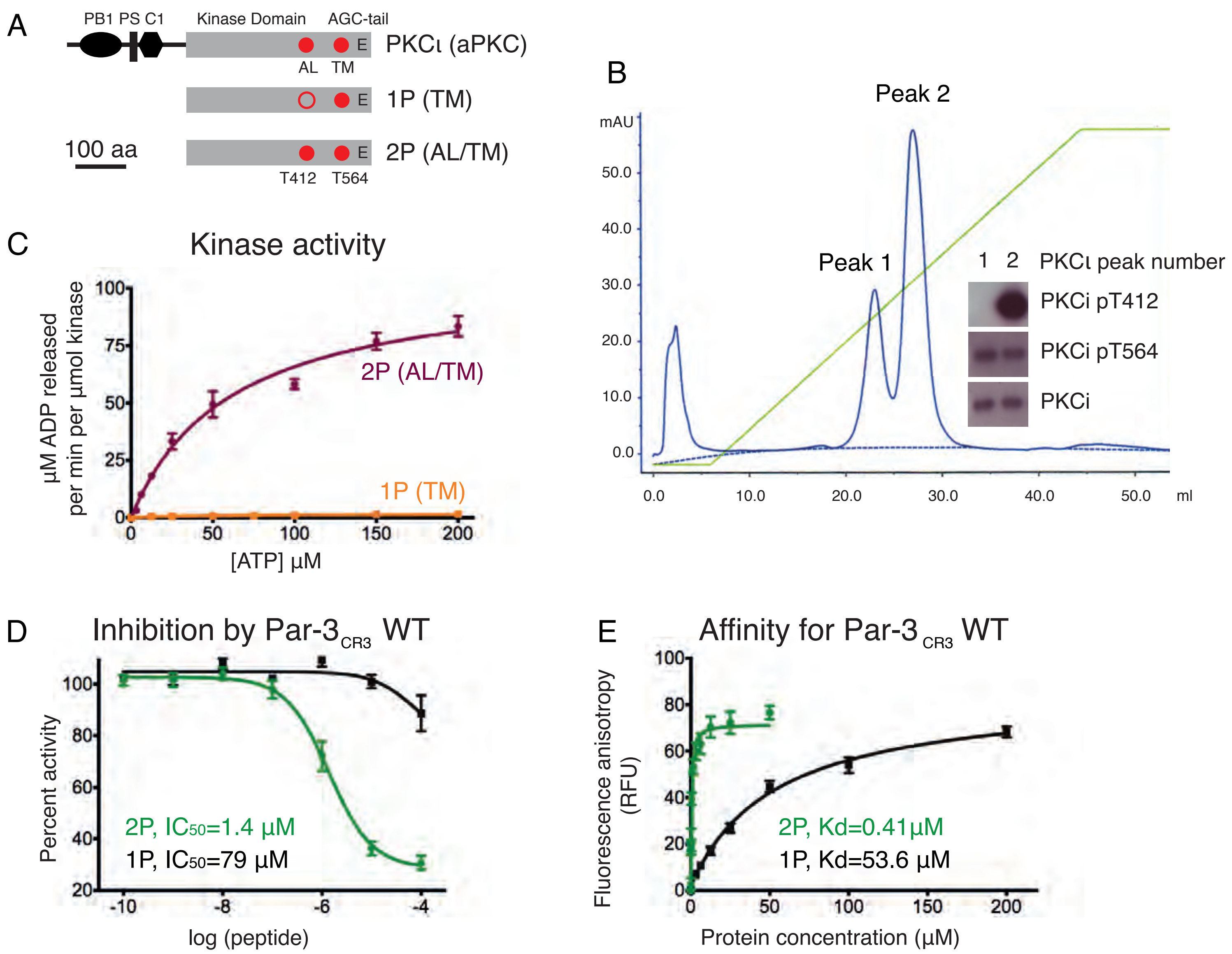


**Purification, characterisation and inhibition of primed (2P) and partially primed (1P) recombinant forms of PKCιKD.**

**(A)** Domain structure of PKCι and the location of the priming phosphorylation sites in the kinase domain. AL (activation loop) contains the PDK1 phospho-site T412 and TM (turn motif) contains the mTOR phosphorylation site T564. **(B)** Purification of recombinant human PKCι kinase domain (PKCιKD) by ion exchange chromatography indicates two discrete species of PKCιKD ; a primed PKCιKD-2P and a partially primed PKCιKD-1P form. Western blot shows the phosphorylation state of PKCιKD in each peak. **(C)** In vitro kinase assay comparing the catalytic activity of the primed PKCιKD-2P and a partially primed PKCι-1P form. **(D)** In vitro kinase assay IC50 curves for Par3CR3 reveals strong inhibition of the primed PKCιKD-2P and very weak inhibition of partially primed PKCιKD-1P forms **(E)** High affinity of fluorescein-labelled Par3CR3 for the primed PKCιKD-2P but low affinity for partially primed PKCιKD-1P as measured by fluorescence anisotropy. Potent PKCιKD-2P inhibition correlates with a high affinity PKCιKD-2P binding.

Figure S3 related to Figure 2 and 3

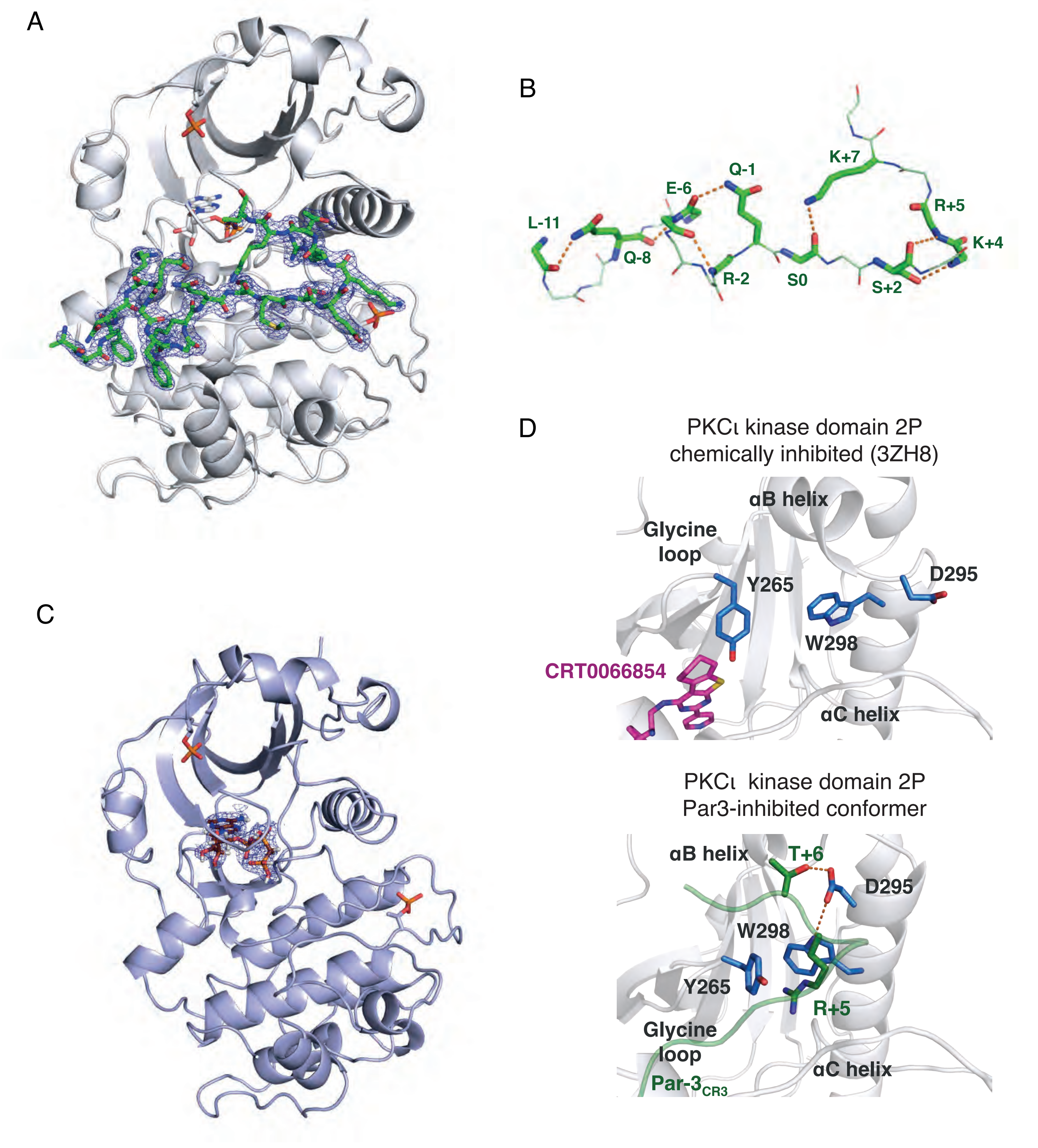


**Electron density for Par3CR3 bound to PKCιKD, its internal hydrogen-bonds and electron density for ATP nucleotide bound to PKCιKD.**
**(A)** Electron density for Par3CR3 peptide superposed onto the structure of Par3CR3 bound to PKCιKD-2P shown with the SIGMAA-weighted 2Fo-Fc electron density omit map (sigma = 1.0). **(B)** Internal hydrogen bonds formed in the Par3CR3 peptide. **(C)** Electron density for AMPPCP superposed onto the structure of AMPPCP bound to PKCιKD-2P shown with the SIGMAA-weighted 2Fo-Fc electron density omit map (sigma = 1.0). **(D)** Close-up of R+5 pocket in the previously solved inhibitor-bound PKCιKD-2P structure (3ZH8) compared to an equivalent view of the Par3 CR3-inhibited structure.

Figure S4 related to Figure 4

A

| | |
|---|---|
| PKCε PS | ERMRPRKRQGAVRRRV |
| PKCε PSS | ERMRPRKRQGSVRRRV |
| FXR short | FKRQGSVRRRV |
| Nishikawa *et al.* | FXRXXSψψ |
| PKCα PS | DVANRFARKGALRQKN |
| PKCι PS | GEDKSIYRRGALRRWR |

B Kinase activity

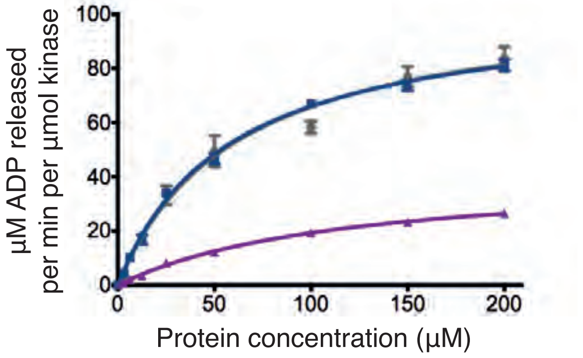


C Affinity

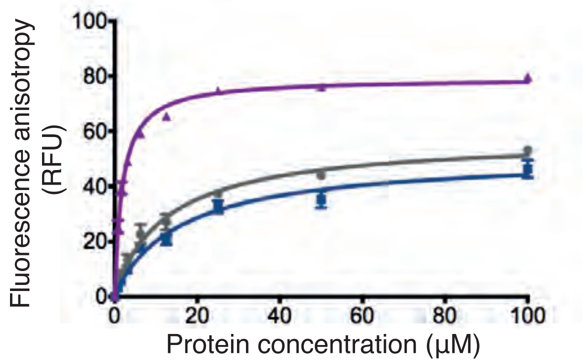


D

| | Sequence | $k_{cat}^{app}$ ($^{-1}$ min) | $k_m^{app}$ (μM) | $k_d$ (μM) |
|---|---|---|---|---|
| $\varepsilon_{pss}$ | ERMRPRKRQGSVRRRVHQVN | 106.1±6 | 61.2±9 | 13.3±2.1 |
| $\varepsilon_{pss}$ -5F* | ERMRPFKRQGSVRRRVHQVN | 42±14 | 118.2±14 | 1.8±0.1 |
| $\varepsilon_{pss}$ +5F | ERMRPRKRQGSVRRRFHQVN | 103.7±2.6 | 57.1±3.9 | 16.4±3.5 |

E

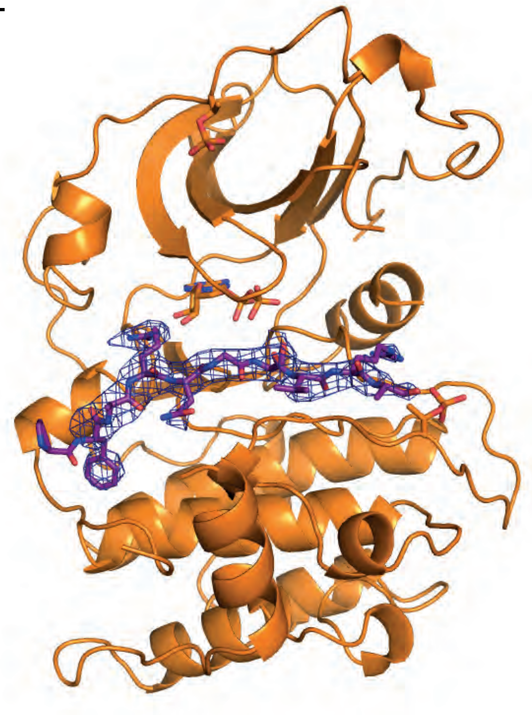

F

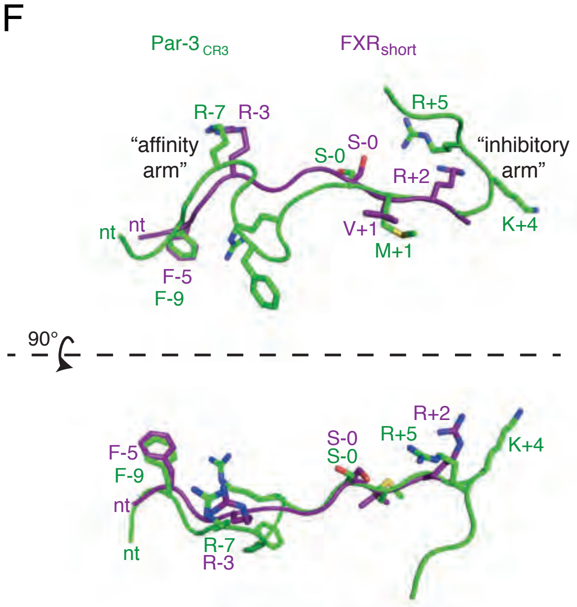

**Characterisation and crystallisation of an FXR-short peptide derived from the PKCε pseudo-substrate motif and resembling an aPKC substrate consensus defined by Nishikawa et al.**

**(A)** Alignment of selected PKC pseudo-substrate sequences with the PKCε pseudo-substrate (PS) and an alanine to serine mutation (PSS), the FXRshort peptide and the consensus aPKC motif first identified by Nishikawa et al. (Nishikawa et al., 1997). The serine phosphorylation site mutation introduced is shown in red and the equivalent alanine residue in pseudo-substrate motifs of PKC isozymes is orange. **(B)** In vitro kinase assay comparing the catalytic activity of PKCιKD-2P towards wild type and mutant PKCεPSS peptides. **(C)** Affinity of fluorescein-labelled εPSS wild type and mutant peptides for PKCιKD-2P measured by fluorescence anisotropy. **(D)** Summary table of kcat, Kd and Km constants between wild type or various εPSS mutants and PKCιKD-2P. Introducing a F-5 residue but not a F+5 residue enhances peptide affinity for PKCιKD-2P and but reduces substrate turnover. This allowed capture of the FXRshort peptide bound to PKC ιKD-2P **(E)** Electron density for FXRshort peptide superposed onto the FXRshort peptide-PKCιKD-2P structure shown with the SIGMAA-weighted 2Fo-Fc electron density omit map (sigma = 1.0). Mn ions and AlF3 were omitted for clarity. **(F)** Orthogonal views of a structural superposition of the Par3CR3/PKCιKD-2P complex with the FXRshort /PKCιKD-2P omitting the aPKC kinase domain for clarity. The FXR motif (F-9/X/R-7)from Par3CR3 superposes precisely onto a structurally equivalent FXR motif (F-5/X/R-3) from the FXRshort peptide.

Figure S5 related to Figure 5

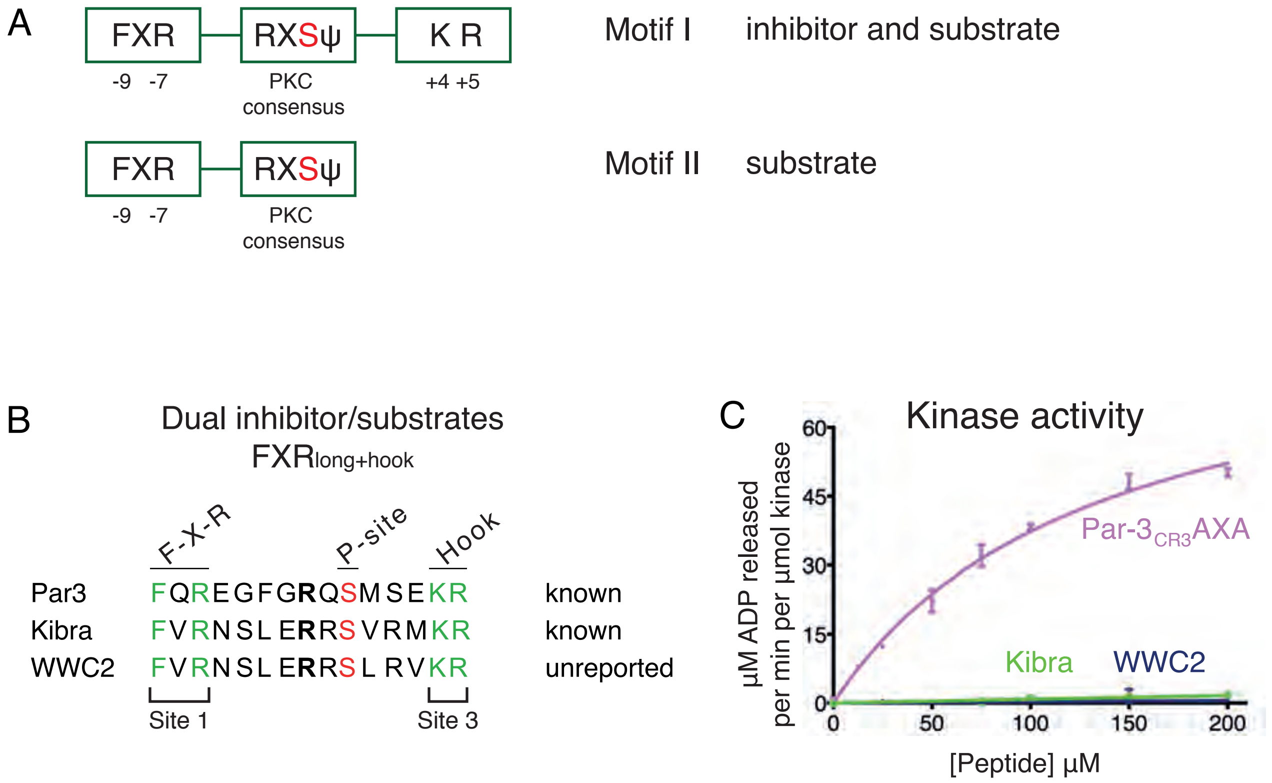


**Presence of K-R inhibitory motif within an aPKC substrate defines a dual inhibitor/substrate function in vitro.**

**(A)** Schematic representation of two groups of aPKC substrates distinguished by the presence or absence of the inhibitory arm. **(B)** Sequence alignment of human Par3, Kibra and WWC2. **(C)** In vitro kinase assay showing that both Kibra and WWC2 inhibit PKCιKD-2P. A Par3CR3 with an A-X-A mutation was used as a positive control (pink) and a Par3CR3 peptide as a negative control (not shown).

Figure S6 related to Figure 5

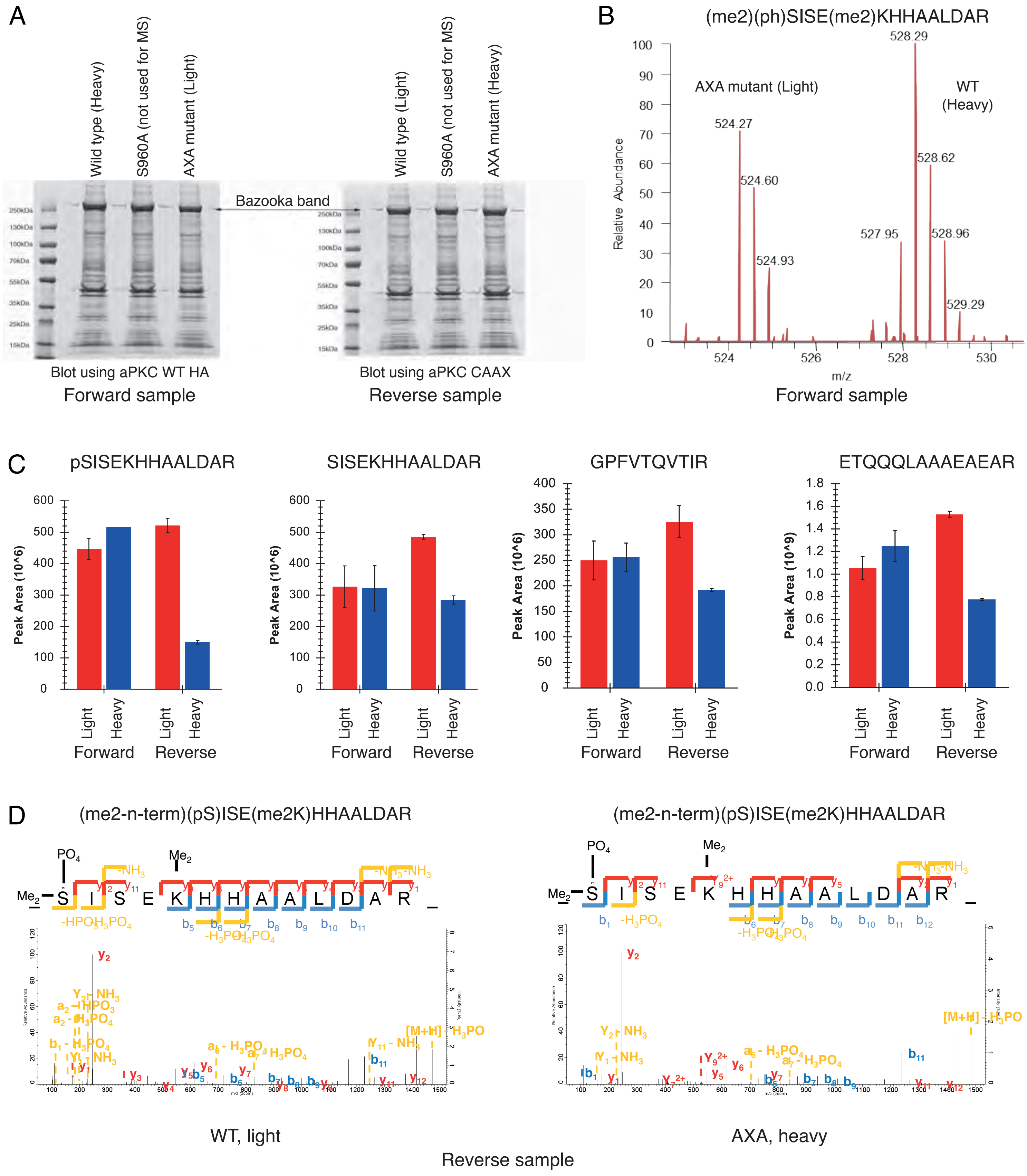


**Wild type Bazooka and A-X-A mutant are phosphorylated at S980 in cell extracts**
**(A)** Purification and labelling scheme of GFP-tagged Bazooka. Both samples were dimethylated but in the forward sample wild type Bazooka was heavy labelled whereas in the reverse sample the A-X-A mutant was heavy labelled. **(B)** Mass spectrum of forward sample displaying the heavy and light peaks, separated by four m/z units. **(C)** Skyline calculated peak areas for light (red) and heavy (blue) Bazooka peptides. Error bars indicate standard errors over triplicate LC-MS injections. Two non-phosphorylated peptides were used as controls **(D)** Annotation of MS/MS spectra for light and heavy peptides using MaxQuant viewer, significant identifications obtained with both MaxQuant/Andromeda and Mascot (data not shown).

Figure S7 related to Figure 6

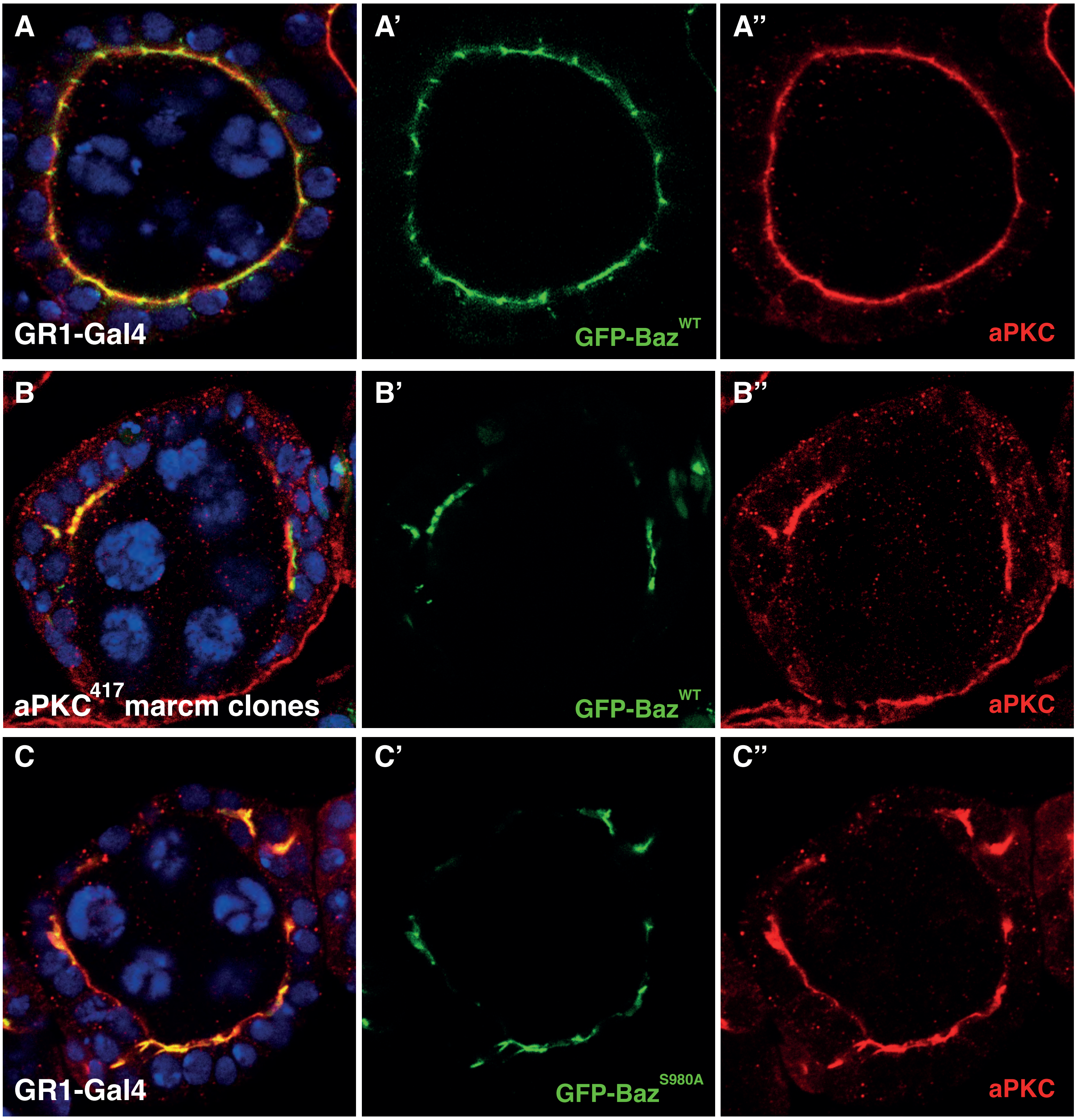


**Bazooka co-localises with aPKC in the absence of kinase activity.**
**(A)** Drosophila egg chambers expressing wild-type Bazooka-GFP in wild-type follicle cells. Note the segregation of aPKC and Baz-GFP, with Baz-GFP also localising to adherens junctions. **(B)** Expression of Bazooka-GFP in kinase dead aPKC mutant cells (aPKC417) leads to either co-localisation with aPKC or complete loss of the apical domain. **(C)** Expression of S980A Bazooka-GFP in wild-type cells leads to co-localisation with aPKC at the apical domain.

## Supplemental Experimental Procedures

### Protein construct design, expression, and purification

The cDNA encoding human PKC iota kinase domain (PKCι$_{KD}$) comprising residues 248-596 (GenBank accession number NM_002740.5) was amplified and inserted into a pBacPAK-His3 vector (Clonetech) using BamHI and XhoI restriction sites. The vector was modified to have a glutathione S-transferase (GST) followed by a 3C protease cleavage site upstream of the kinase domain. A C586S mutation was also introduced in the kinase domain using QuikChange (Strategene) using standard protocols.

Viruses used for infection were obtained under standard protocol (Oxford Expression Technology UK). PKCι virus was used to infect Hi5 or SF21 cells with a multiplicity of infection of 2 with cells were grown in shaker flasks in SFIII media (Life Technologies) and 10 μg/ml gentamycin (Life Technologies). Cells were harvested 72 hours post infection and resuspended in lysis buffer (20 mM HEPES pH 7.4 (Sigma), 150 mM NaCl (Sigma), 10 mM Benzamidine (Sigma), 0.2 mM AEBSF (Melford Laboratories), 1 mM EDTA (Sigma), and 1 mM DTT (Melford Laboratories)). All purification steps were done at 4 °C or on ice. Cells were lysed by sonication and spun down at 30,000*g* for 30 minutes. PKCι$_{KD}$ was purified using glutathione sepharose 4B beads (Amersham Biosciences), followed by removal of the GST affinity tag with GST-3C protease (PreScission Protease, Amersham Bioscience) and ion-exchange chromatography (Hi-Trap Q column, GE Healthcare). The major peak from ion-exchange is not phosphorylated at the activation loop T412, but is phosphorylated at the turn motif site T564 (PKCι$_{KD}$-1P) and used as a control in the kinetic and binding assays. To produce the primed kinase domain (PKCι$_{KD}$-2P) phosphorylated at both T412 and T564, Hi5 cells were co-infected with PDK-1 virus at a multiplicity of infection of 1. All other expression and purification procedures remain the same. Both proteins were dialyzed into the final buffer (20 mM Tris pH 7.5, 50 mM NaCl, 5% glycerol, 1 mM DTT) and concentrated before use.

### *In vitro* kinetic assays for PKCι$_{KD}$-2P

The ADP Quest kit (DiscoveRx) was used to determine the $k_{cat\ app}$ and $K_{m\ app}$ values for ATP against the PKCι$_{KD}$-1P and PKCι$_{KD}$-2P. The assay uses a coupled reaction to convert ADP to a product that has a fluorescence excitation at 530 nm and emission at 590 nm. Several synthetic peptide substrates were used including a model peptide (ERMRPRQGSVRRRV) derived from a PKCε pseudo-substrate sequence with an alanine-serine change. We found this peptide to be an efficient substrate peptide for aPKC isozymes. Peptides from human Par3$_{CR3}$ (residues 816-835) and F-X-R$_{short}$ peptide (ERMFPRQGSVRRRV) related to the Nishikawa and co-workers consensus were also used in the study (Nishikawa et al., 1997). For the $k_{cat\ app}$ and $K_{m\ app}$ reactions, the ATP concentration ranged from 0-200 μM, while the synthetic peptide substrates were kept constant at 200 μM to avoid product inhibition. The reactions were measured every two minutes for 30 minutes in a 384-well plate using a Safire$^2$ plate reader (Tecan). The kinetic constants were determined by fitting the data to Michaelis–Menten equation using the graphics program Prism (GraphPad Prism, version 5.0d, GraphPad Software). To determine the $IC_{50}$ of various Par3$_{CR3}$ peptides, the Par3$_{CR3}$ peptide concentration was varied from 10 nM to 0.1 mM, with the model peptide and ATP concentration kept at a constant 20 μM each. The reactions were then scanned after 30 minutes and the $IC_{50}$ was determined using a non-linear fit in the graphics program Prism.

**Fluorescence anisotropy binding assays**

Fluorescence anisotropy assays were performed to determine the $K_d$ for each peptide following a previously described protocol (Guettler et al., 2008). Binding assays were performed in 20 mM Tris pH 7.5, 50 mM NaCl, 5% glycerol and 0.5 mM TCEP. The reaction mixtures contained fluorescein labelled peptides at a constant 50 nM concentration, while the protein concentration ranged from 0-200 μM. The 20 μl reactions were carried out in 384-well plate and measured after 30 minutes on the Safire$^2$ plate reader (Tecan) with the excitation at 470 nm and emission at 525 nm. The anisotropy values were normalized and the $K_d$ was determined using a nonlinear regression with the graphics program Prism and an equation previously described (Heyduk and Lee, 1990).

**Structure determination of nucleotide-bound PKCι$_{KD}$-2P with Par3$_{CR3}$**

$PKC\iota_{KD}$-2P was incubated with a 3 molar excess of AMPPNP, magnesium sulphate and a 3 molar excess of human Par3 peptide (residues 816-841) for an hour on ice. Crystallization was performed using hanging drops method with 1:1 ratio of protein to precipitant at 20 °C. Crystals grew in 2-3 weeks using 32% Peg 2000 MME and 0.08 M KSCN and were cryo-cooled using 20% glycerol. These crystals belonged to $P3_121$ space group. Data were collected at ESRF ID-29. Data were processed in XDS (Kabsch, 2010b) Xscale (Kabsch, 2010a), with molecular replacement performed using Phaser (McCoy et al., 2007) using a previous PKCι-2P structure as a search model (PDB code: 3A8W) with the activation loop and other flexible loops removed from the search model. These trigonal crystals diffracted to Bragg spacings of 2.0 Å with one molecule in the asymmetric unit. Initial rigid body refinement in Phenix (Adams et al., 2010) was followed by model building in COOT (Emsley and Cowtan, 2004). Further refinement was carried out in Phenix before the AMP-PNP molecule was modelled into the density. No density was observed for the γ-phosphate of AMPPNP consistent with the AMPPNP being hydrolysed to AMPPN and inorganic phosphate under these crystallisation conditions. $Par3_{CR3}$ peptide residues 816 to 835 were well ordered with good density, whereas residues 836-841 could not be modelled, presumed to be disordered. Two Mg ions were evident in the structure, one bridged by the α and β-phosphates of AMPPN and a second site bridged $T833^{Par3}$ and $D295^{PKC\iota}$ (Figure 2G).

**Structure determination of nucleotide-bound $PKC\iota_{KD}$-2P**

$PKC\iota_{KD}$-2P was incubated with a 3 molar excess of AMPPCP for an hour on ice. Crystallization was performed using hanging drops method with 1:1 ratio of protein to precipitant at 20 °C. Crystals grew in 2-3 days using 25% Morpheus precipitant mix 4 (Molecular dimensions), 10% Carboxylic acids (Molecular dimensions), Buffer system 1 pH=6.3 (Molecular dimensions) and were cryo-cooled without additional cryoprotectant. These crystals belonged to $P2_12_12_1$ space group. Data were collected at Diamond I24 and were processed as above. Molecular replacement was performed using Phaser (McCoy et al., 2007) using a previous PKCι-2P structure as a search model (PDB code: 3A8W) with the activation loop and other flexible loops removed from the search model. These crystals diffracted to Bragg spacings of 1.8 Å with one molecule in the asymmetric unit. The data was solved and refined using Phaser and

Phenix as described above. Further refinement was carried out in Phenix before the AMPPCP molecule was modelled into the density.

**Structure determination of nucleotide-bound $PKC\iota_{KD}$-2Pwith a $FXR_{short}$ substrate peptide**

$PKC\iota_{KD}$-2P was incubated with a 3 molar excess of ADP, manganese sulphate, 12 molar excess of $AlF_3$ (a known transition-state analogue for phospho-transfer) and a 3 molar excess of the $FXR_{short}$ peptide substrate (ERMRPFKRQGSVRRRV) for an hour on ice. Crystallization was performed using hanging drop method with 1:1 ratio of protein to precipitant at 20 °C. The protein at crystallized in 32% Peg 2000 MME and 0.08 M KSCN and was frozen in cryo-protectant containing 32% Peg 2000 MME, 0.08 M KSCN, and 20% glycerol. Data were collected at Diamond IO4. Data were processed using D*Trek (Pflugrath, 1999), and scaled with Scala and Pointless (Collaborative Computational Project-Number 4, 1994). The data was solved and refined using Phaser and Phenix as described above.

**Cell culture and transfection**

HCT-116 cells grown in McCoy's 5A medium containing 10% bovine foetal calf serum and penicillin/streptomycin (Invitrogen) were transfected (10 μg portion of DNA or 5 μg + 5 μg portions of DNA for co-transfections) using Fugene HD (Roche) according to the manufacturer's instructions. The cells were then grown in normal medium for 36 h.

**Antibodies**

The following antibodies were used for immunoblotting: mouse monoclonal anti-PKCι (recognition for human PKCι), mouse monoclonal anti-Myc (9E10), rabbit polyclonal anti-Par3 (Millipore), rabbit polyclonal anti-GFP antibody (Santa Cruz). Anti-phospho-S827 Par3 antibody was raised in-house using CQREGFGRQSMSEKRTKQ as an antigen, lacking the F-X-R site of $Par3_{CR3}$. For Western blots using the phospho-specific pPAR3 Ab, 100ng/ml of the de-phospho peptide to compete out during the primary Ab incubation.

**Plasmids and mutagenesis**

pEGFP-PKCi-WT, pEGFP-PKCi-DD/AA (D339A/D382A); pK-myc-Par3-WT (addgene, plasmid 19388), pK-myc-Par3-AXA (F818A/R820A) and pK-myc-Par3-A (S827A) included human PKCι and Par3 cDNAs. Mutagenesis of PKCι and Par3 was performed using the QuikChange Site-Directed Mutagenesis Kit (Stratagene) according to the manufacturer's instructions. The nucleotide changes were verified by in-house DNA sequencing.

**Immunoprecipitation**

After 36 h, transfected HCT-116 cells were lysed in lysis buffer LB (1% Triton X-100, 20 mM Tris–HCl, pH 8, 130 mM NaCl, 1 mM dithiothreitol (DTT), 10 mM sodium fluoride, complete EDTA-free protease inhibitor cocktail (Roche), phosphatase inhibitor cocktails (set II+IV, Calbiochem)). After centrifugation (13,000*g*, 4 °C, 10 min), soluble proteins were pre-cleared then incubated with anti-GFP magnetic beads (GFP-TRAP; Chromotek) or anti-Myc agarose beads (sigma) for 90 min or 2 h respectively at 4 °C. Beads were washed 5 times with lysis buffer (containing 260 mM NaCl), then the bound proteins were eluted in Laemmli sample buffer, resolved by SDS–PAGE and analysed by immunoblotting.

**Immunoblotting**

For immunoblotting, proteins from samples in Laemmli sample buffer were separated using precast NuPAGE 4-12% Bis-Tris gels (Invitrogen), transferred to nitrocellulose membranes (PROTRAN, Whatman), and incubated with antibodies diluted in Tris-buffered saline containing 5% non-fat milk and 0.1% Tween-20 for immunodetection.

**Dimethyl labelling and quantification of Bazooka wild type and mutant S980 phosphorylation in S2 cell extracts.**

After SDS-PAGE, in-gel stable isotope dimethyl labelling was performed according to published protocols (Boersema et al., 2009). The heavy reaction was performed using $^{13}CD_2O$ formaldehyde creating a mass difference of 6 Daltons per primary amine group between heavy and light dimehtylated peptides. After extensive washing of gel pieces, the in-gel dimethylated proteins were then subjected to overnight in-gel trypsin digestion at 37$^{o}$C. The following day peptides were extracted and subjected to another round of reductive dimethylation reactions aimed at methylating peptide n-

termini. Peptide mixtures were acidified and prepared for LC-MS analysis using an Ulimate3000 HPLC coupled to a Q-Exactive mass spectrometer (Thermofisher). A targeted scan was performed for the S980 containing peptides and this was alternated with top10 data-dependent acquisition scan. Mascot generated DAT files were converted to Skyline compatible biblio.spec libraries and heavy and light peak areas were extracted by Skyline software version 2.5.0.6079 (MacLean et al., 2010).

### *Drosophila* genetics and oligonucleotides

Expression of *UAS*-driven transgenes in follicle cells was achieved with the GR1.Gal4 line. *UAS.GFP-Baz* lines were constructed by mutagenising the full-length Baz cDNA in pDONR, followed by transfer to the pPGW (pUASP-EGFP-Gateway) vector for transgenesis (Bestgene, Inc). The *UAS.GFP-BazS980E* line was a gift from F. Pichaud. The following primers were used for mutagenesis:

**Wild-type *Drosophila* Bazooka:**

S L E T N S G V E H **F** S **R** D A L G R R **S**
AGCTTGGAGACAAACTCGGGCGTGGAGCAT**TTC**TCG**CGC**GATGCTTTGGGACGACGC**AG**
**C**
I S E K H H A A L D A R E T G T Y Q R N
ATCTCTGAGAAGCACCATGCGGCGCTGGATGCCCGCGAAACTGGCACCTATCAGCGGAAT

**The FXR mutant:**

S L E T N S G V E H **A** S **A** D A L G R R **S**
AGCTTGGAGACAAACTCGGGCGTGGAGCAT**GCC**TCG**GCC**GATGCTTTGGGACGACGC**AG**
**C**
I S E K H H A A L D A R E T G T Y Q R N
ATCTCTGAGAAGCACCATGCGGCGCTGGATGCCCGCGAAACTGGCACCTATCAGCGGAAT

5’ CTCGGGCGTGGAGCAT**GCC**TCG**GCC**GATGCTTTGGGACGA 3’ sense
5’ TCGTCCCAAAGCATCGGCCGAGGCATGCTCCACGCCCGAG 3’ anti-sense

**The S980A mutant:**

S L E T N S G V E H **F** S **R** D A L G R R **A**
AGCTTGGAGACAAACTCGGGCGTGGAGCAT**TTC**TCG**CGC**GATGCTTTGGGACGACGC**GC**
**C**
I S E K H H A A L D A R E T G T Y Q R N
ATCTCTGAGAAGCACCATGCGGCGCTGGATGCCCGCGAAACTGGCACCTATCAGCGGAAT

5’ GCTTTGGGACGACGC**GCC**ATCTCTGAGAAGCAC 3’ sense
5’ GTGCTTCTCAGAGATGGCGCGTCGTCCCAAAGC 3’ anti-sense

**The FXR / S980A mutant:**

S L E T N S G V E H **A** S **A** D A L G R R **A**
AGCTTGGAGACAAACTCGGGCGTGGAGCAT**GCC**TCG**GCC**GATGCTTTGGGACGACGC**GC**
**C**
I S E K H H A A L D A R E T G T Y Q R N
ATCTCTGAGAAGCACCATGCGGCGCTGGATGCCCGCGAAACTGGCACCTATCAGCGGAAT

CGGGCGTGGAGCAT**GCC**TCG**GCC**GATGCTTTGGGACGACGC**GCC**ATCTCTGAGAAGCAC sense
GTGCTTCTCAGAGATGGCGCGTCGTCCCAAAGCATCGGCCGAGGCATGCTCCACGCCCG anti-s

**Wild-type *Drosophila* Bazooka:**
I S E **K H** H A A L D
5' ATCTCTGAG**AAGCA**CCATGCGGCGCTGGAT 3'

**The KH mutant:**
I S E **A A** H A A L D
5' GACGCAGCATCTCTGAGGCGGCCCATGCGGCGCTGGATG 3' sense
3' CATCCAGCGCCGCATGGGCCGCCTCAGAGATGCTGCGTC 3' anti-sense

### *Drosophila* antibodies and immunohistochemistry

Ovaries were dissected in PBS, fixed for 20 mins in 4% paraformaldehyde in PBS, washed for 30 minutes in PBS/0.1% Triton X-100 (PBST) and blocked for 15 minutes in 5% normal goat serum/PBST (PBST/NGS). Primary antibodies were diluted in PBST/NGS and samples were incubated overnight at 4 °C. Either optical cross-sections through the middle of egg chambers or apical sections of the follicular epithelium are shown.

Primary antibodies used were: rabbit anti-PKCζ (C-20) (1:250, Santa Cruz), mouse anti-E-cad (1:100 DSHB), mouse anti-Dlg (1:250, DSHB), rabbit anti-Bazooka (1:250, A.Wodarz). Secondary antibodies (all from Molecular Probes, Life) were used at 1:500 for 2 hours prior to multiple washes in PBST and staining with DAPI at 1 μg/ml for 10-30 mins prior to mounting on slides in Vectashield (Vector labs). Images were taken with a Leica SP5 confocal and processed with Adobe Photoshop.

**Supplementary References**


Adams, P.D., Afonine, P.V., Bunkoczi, G., Chen, V.B., Davis, I.W., Echols, N., Headd, J.J., Hung, L.W., Kapral, G.J., Grosse-Kunstleve, R.W., *et al.* (2010). PHENIX: a comprehensive Python-based system for macromolecular structure solution. Acta Crystallogr D Biol Crystallogr *66*, 213-221.

Collaborative Computational Project-Number 4 (1994). The CCP-4 suite: programs for protein crystallography. Acta Crystallogr D *50*, 760-763.

Emsley, P., and Cowtan, K. (2004). Coot: model-building tools for molecular graphics. Acta Crystallogr D *60*, 2126-2132.

Guettler, S., Vartiainen, M.K., Miralles, F., Larijani, B., and Treisman, R. (2008). RPEL motifs link the serum response factor cofactor MAL but not myocardin to Rho signaling via actin binding. Mol Cell Biol *28*, 732-742.

Heyduk, T., and Lee, J.C. (1990). Application of fluorescence energy transfer and polarization to monitor Escherichia coli cAMP receptor protein and lac promoter interaction. Proc Natl Acad Sci U S A *87*, 1744-1748.

Kabsch, W. (2010a). Integration, scaling, space-group assignment and post-refinement. Acta Crystallogr D Biol Crystallogr *66*, 133-144.

Kabsch, W. (2010b). XDS. Acta Crystallogr D Biol Crystallogr *66*, 125-132.

MacLean, B., Tomazela, D.M., Shulman, N., Chambers, M., Finney, G.L., Frewen, B., Kern, R., Tabb, D.L., Liebler, D.C., and MacCoss, M.J. (2010). Skyline: an open source document editor for creating and analyzing targeted proteomics experiments. Bioinformatics *26*, 966-968.

McCoy, A.J., Grosse-Kunstleve, R.W., Adams, P.D., Winn, M.D., Storoni, L.C., and Read, R.J. (2007). Phaser crystallographic software. J Appl Crystallogr *40*, 658-674.

Morais-de-Sa, E., Mirouse, V., and St Johnston, D. (2010). aPKC phosphorylation of Bazooka defines the apical/lateral border in Drosophila epithelial cells. Cell *141*, 509-523.

Nishikawa, K., Toker, A., Johannes, F.J., Songyang, Z., and Cantley, L.C. (1997). Determination of the specific substrate sequence motifs of protein kinase C isozymes. J Biol Chem *272*, 952-960.

Pflugrath, J.W. (1999). The finer things in X-ray diffraction data collection. Acta Crystallogr D Biol Crystallogr *55*, 1718-1725.
